# Bisecting βGlcNAc: *MA’AT* analysis of ^13^C-labeled oligosaccharides containing βGlcNAc-(1→4)-βMan *O*-glycosidic linkages

**DOI:** 10.1016/j.jbc.2025.111108

**Published:** 2025-12-23

**Authors:** Wenhui Zhang, Reagan J. Meredith, Jieye Lin, Mi-Kyung Yoon, Ian Carmichael, Anthony S. Serianni

**Affiliations:** 1 Department of Chemistry and Biochemistry, University of Notre Dame, Notre Dame, Indiana, USA; 2 Omicron Biochemicals, Inc., South Bend, Indiana, USA; 3 Texas Biomedical Research Institute, San Antonio, Texas, USA; 4 Department of Biological Chemistry, UCLA, Los Angeles, California, USA; 5 Radiation Laboratory, University of Notre Dame, Notre Dame, Indiana, USA

**Keywords:** carbohydrate structure, conformational change, glycobiology, nuclear magnetic resonance (NMR) spectroscopy, oligosaccharide, molecular dynamics, molecular modeling, *MA’AT* analysis, bisecting βGlcNAc, context effects

## Abstract

Biologically-relevant mannose- (Man) and *N*-acetyl-D-glucosamine- (GlcNAc) containing di-, tri-, tetra- and hexasaccharides containing βGlcNAc-(1→4)-βMan *O*-glycosidic linkages were selectively labeled with ^13^C and used in *MA’AT* analyses of context effects on linkage conformation and dynamics. Using βGlcNAc-(1→4)-βManOCH_3_ as the reference disaccharide, *MA’AT* analysis provided experiment-based probability distributions for the *phi* (*ϕ*) and *psi* (*ψ*) torsion angles that comprise its linkage, giving mean values and circular standard deviations, a measure of librational motion about the mean angle, for each angle devoid of context effects. *MA’AT* analyses of the larger oligosaccharides revealed how the βGlcNAc-(1→4)-βMan linkage behaves conformationally when embedded into larger structures. Context effects on *ϕ* were generally small (changes in mean values <6°), whereas those on *ψ* were substantial (changes in mean values up to 25°). Substantial reduction in librational averaging of *ψ* was observed in the highly-congested hexasaccharide. Similar effects were observed for αMan-(1→3)-βMan linkages, where context effects on *ϕ* were negligible but those on the mean values of *ψ* significant (∼14°). The experimental results were compared to those obtained by aqueous molecular dynamics simulation. The results demonstrate the ability of *MA’AT* analysis to detect and quantify changes in linkage conformational equilibria in solution brought about by structural context, and point to the relative rigidity of *ϕ* in response to structural crowding compared to *ψ*, the latter being more responsive to local environment. Embedding a bisecting βGlcNAc residue into biologically-relevant Man-containing oligosaccharides causes substantial change in both linkage conformational preference and librational averaging of linkage torsion angles.

*N*-Glycosylation is an important co-translational modification of proteins in eukaryotes and some prokaryotes (1, 2, 3). *N*-Glycans are covalently attached to protein *via* βGlcNAc-(1→N^γ^)-Asn linkages, and the resulting plasma-membrane-bound *N*-linked glycoproteins participate in a host of biological processes, serving as mediators of cell-cell recognition, bacterial and viral infection, inflammation, and other biological processes (4, 5). *N*-Glycan attachment to protein and some trimming of the initially-installed Glc_3_Man_9_GlcNAc_2_ 14-mer begins in the lumen of the endoplasmic reticulum, eventually leading to chain maturation in the Golgi *via* the sequential action of glycosidases, sugar nucleotides and/or glycosyltransferases (2). One of the latter enzymes, *N*-acetylglucosaminyltransferase III (GNT-III), transfers βGlcNAc residues from UDP-GlcNAc to the βMan residue of the Man_3_GlcNAc_2_ core pentasaccharide, producing a βGlcNAc-(1→4)-βMan linkage in bisecting hybrid or complex *N*-glycans such as **1** and **2** (Fig. 1) (6, 7, 8, 9). βGlcNAc-Bisected *N*-glycans have been implicated in several disease processes, including the regulation of tumor progression and migration, and Alzheimer’s disease (8, 10). βGlcNAc-Bisected *N*-glycans also serve as general suppressors of *N*-glycan terminal modification (11).

**Figure 1:**
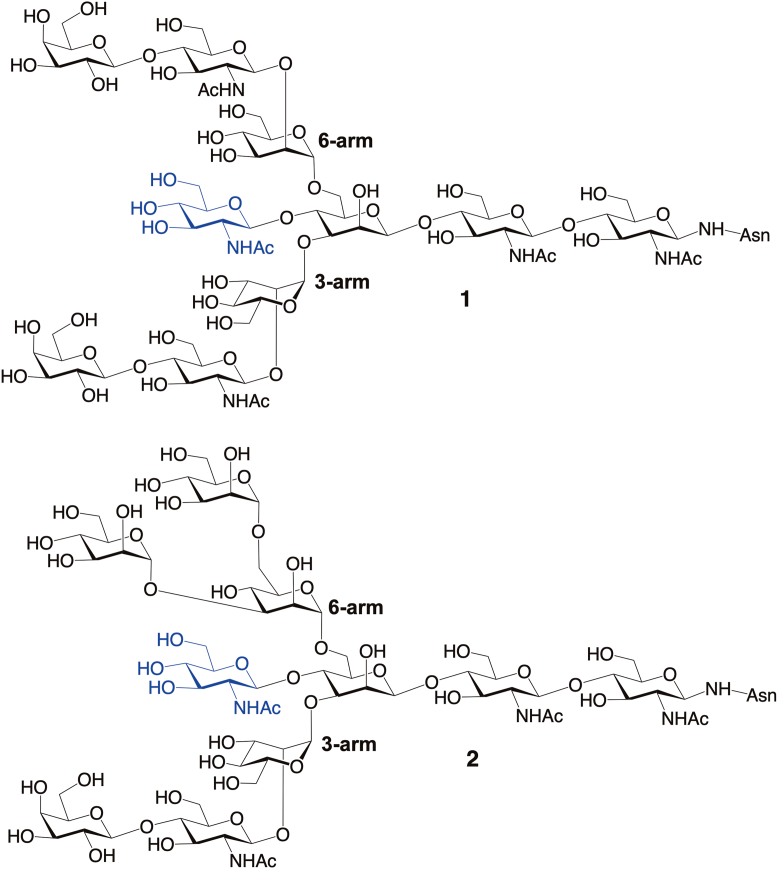
**Bisecting βGlcNAc residues in complex (1) and hybrid (2) *N*-glycans.** The bisecting residues are shown in *blue*. The 3- and 6-arms extending from the βMan core residue are identified.

The backbone three-dimensional structure of an *N*-glycan chain is largely determined by the conformational properties of its constituent *O*-glycosidic linkages. As observed in oligopeptides and oligonucleotides, oligosaccharides adopt more than one 3D structure, with individual conformers exhibiting different biological activities. For example, statistical analyses of crystal structures of lectin-glycan complexes suggest that bound glycan conformations reflect stable or metastable conformations in solution (12, 13). The addition of bisecting βGlcNAc residues to an *N*-glycan chain not only changes its primary structure but also may disturb the conformational equilibria of other glycosidic linkages, notably those comprising the proximal 3- and 6-arms (Fig. 1). New biological functions associated with bisected structures appear to correlate with changes in linkage conformational equilibria in solution (14, 15).

Structural studies of the effect of bisecting βGlcNAc on *N*-glycan solution conformation have been conducted by nuclear magnetic resonance (NMR) using ^1^H-^1^H nuclear overhauser effect (NOEs) and spin-coupling constants as experimental constraints (16, 17, 18, 19) and by MD simulation (20, 21). The experimental NMR studies have been largely qualitative, relying on a limited number of experimental parameters that requires a reliance on MD simulation and related computational methods to draw structural conclusions. Recently, a new experimental NMR method has been introduced, *MA’AT* analysis, that uses multiple, redundant NMR *J*-couplings and circular statisics to obtain probability distributions of *O*-glycosidic linkage torsion angles in solution (Fig. 2) (22, 23, 24, 25, 26). In methyl 2-acetamido-2-deoxy-β-D-glucopyranosyl-(1→4)-β-D-mannopyranoside (**3**), at least five *J*-couplings are sensitive to linkage torsion angle *ϕ* (defined as H1′–C1′–O1′–C4) and six are sensitive to linkage torsion angle *ψ* (defined as C1′–O1′–C4–H4). Some of these *J*-couplings are conventional and some are nonconventional, as defined in Fig. 2 (27, 28). The latter are ^1^*J* and ^2^*J* values that are not commonly used in conformational analyses of *O*-glycosidic linkages (28). Analogous trans-*O*-glycosidic *J*-couplings exist for the other *O*-glycosidic linkages in **1** and **2** involving two or three bonds (Scheme S5, Supporting Information).

**Figure 2:**
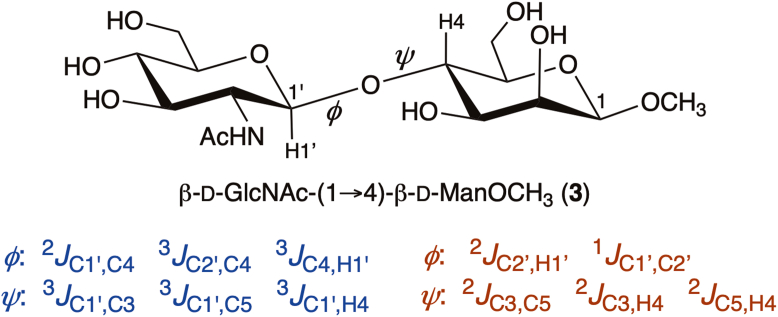
**Conventional and nonconventional trans-*O*-glycosidic *J*-couplings in β-D-GlcNAc-(1→4)-**β-**D-ManOCH_3_ (3) that depend on linkage torsion angles**ϕ**and**ψ. Conventional values are shown in *blue* and nonconventional values are shown in *red*. Conventional and nonconventional (^2^*J*_C2′,H1′_) values were used in *MA’AT* analyses of ϕ in **3** and in corresponding ϕ in **5**, **6** and **8**–**10**, but only three conventional values were used in *MA’AT* analyses of ψ in these compounds. C1′, C1, H1′ and H4 are identified in the residues of **3**.

This report describes *MA’AT* analyses of the *O*-glycosidic linkages in several oligosaccharide sub-fragments of **1** and **2** containing a bisecting βGlcNAc (Fig. 3). Oligosaccharides **3**, **5**, **6** and **8** were prepared with site-specific ^13^C-labeling to enable measurements of ^13^C-^13^C spin-couplings across their *O*-glycosidic linkages and/or to simplify the measurements of intra- and inter-residue ^13^C-^1^H spin-couplings. Disaccharide **3** contains a βGlcNAc-(1→4)-βMan linkage devoid of context effects (the reference state). *MA’AT* analyses of the same linkage in **5**, **6**, and **8** provide information on context effects of O3 and O6 substitution of the βMan residue on βGlcNAc-(1→4)-βMan linkage conformation. Insertion of ^13^C in the αMan residues of **6** and **8** allowed assessments of the αMan-(1→3)-βMan linkage when βGlcNAc is present at O4 of βMan through comparisons to corresponding linkage behavior studied previously in **4** and **7** (25). The conformational properties of the αMan-(1→6)-βMan linkages in **5** and **8** will be treated in an upcoming report. The βGlcNAc-(1→4)-βMan linkages in ^13^C-labeled tetrasaccharide **9** and hexasaccharide **10** were also investigated as model systems, since neither is a sub-fragment of **1** or **2**.

**Figure 3:**
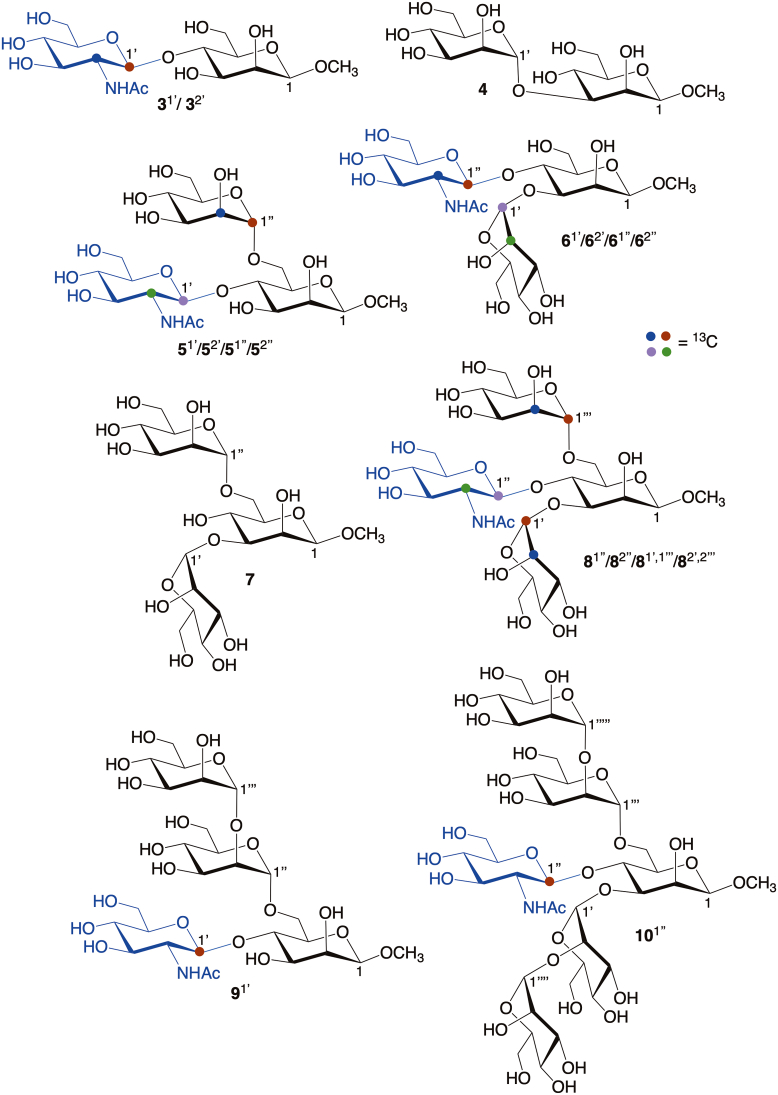
**Singly- and multiply-^13^C-labeled oligosaccharides 3, 5-6 and 8-10 containing βGlcNAc-(1→4)-βMan and αMan-(1→3)-βMan *O*-glycosidic linkages studied by *MA’AT* analysis**. The αMan-(1→2)-αMan and αMan-(1→6)-βMan linkages in these compounds were not studied in this work. Compounds **4** and **7** were investigated previously (25). βGlcNAc residues are highlighted in blue. Anomeric carbon atoms in each residue of **3**–**10** are identified as 1, 1′, 1’’, 1’’’,1’’’’ or 1’’’’’. ^13^C Isotopomers of **3**, **5**-**6** and **8**-**10** are identified by superscripts (*e.g.,***8**^1′,1’’’^ was doubly ^13^C-labeled at C1′ and C1’’’).

## Results

### ^1^H and ^13^C chemical shifts and intra-residue NMR spin-couplings

Corresponding intra-ring ^3^*J*_H1,H2_, ^3^*J*_H2,H3_, ^3^*J*_H3,H4_ and ^3^*J*_H4,H5_ values in the βGlcNAc, αMan and βMan residues of **3**, **5**, **6** and **8** are very similar (differences of ≤ 0.3 Hz) (Table S1, Supporting Information), indicating that ring conformation is minimally affected by structural context and that ^4^*C*_1_ chair forms are highly preferred. Corresponding intra-residue *J*_CC_ values in the βGlcNAc, αMan and βMan residues are also very similar and are consistent with preferred ^4^*C*_1_ chair forms indicated by the ^3^*J*_HH_ values (Table S2, Supporting Information). For example, ^2^*J*_C1,C3_ = +4.5 – +4.7 Hz in βGlcNAc, consistent with a C1–C2–C3 coupling pathway having both terminal oxygen atoms equatorial (29). ^2^*J*_C1,C3_ in αMan is essentially zero, consistent with the same pathway having one terminal oxygen atom axial and the other terminal oxygen atom equatorial (29). ^2^*J*_C1,C5_ is ∼ –2.0 Hz in αMan and essentially zero in βGlcNAc, consistent with the effects of anomeric configuration on ^2^*J*_C1,C5_ magnitude (30). ^2^*J*_C2,C4_ values are +2.1 – +2.4 Hz in βGlcNAc and essentially zero in αMan, consistent with the former C2–C3–C4 pathway bearing two terminal equatorial oxygen atoms and the latter having one axial and one equatorial (29). The consistently smaller ^3^*J*_C1,C6_ values in αMan compared to βGlcNAc reflects the effect of terminal anomeric oxygen atoms on coupling magnitude; an in-plane O1 adds ∼0.7 Hz to the value observed when O1 is out-of-plane (30, 31).

^1^H Chemical shift changes were observed in the βMan residues of **5**, **6**, and **8-10**, relative to the ^1^H shifts of the βMan residue of disaccharide **3** (Tables S3 and S4, Supporting Information). The largest changes (all downfield shifts) were observed, as expected (32), at hydrogens appended to carbons involved in *O*-glycosylation or are on carbons proximal to them. For example, δ_H5_ and δ_H6b_ in the βMan residues of **5** and **9** increase by +0.12 – +0.15 ppm. In **6**, δ_H3_ and δ_H4_ increase by +0.14 and + 0.22 ppm, respectively. In **8**, δ_H3_ and δ_H4_ increase by +0.13 and +0.27 ppm, whereas in **10** these increases are +0.06 and +0.30 ppm, respectively. Substitution effects are probably the dominant structural factor responsible for these changes, but conformational differences may also contribute, especially for δ_H3_ in **6**, **8** and **10** and δ_H6b_ in **5** and **9** (see discussion below).

^13^C Chemical shift changes in the βMan residues of **5**, **6**, and **8-10** were also observed relative to values observed in **3** (Tables S3 and S4, Supporting Information). Significant changes were observed for carbons involved in *O*-glycosylation, and in all cases the shifts were downfield as expected (32). For example, δ_C6_ in the βMan residues in **5** and **8**–**10**, and δ_C3_ in the βMan residues of **6**, **8** and **10** increase by ∼+5.4 ppm. In addition, δ_C4_ in **6**, **8** and **10** decreases significantly even though this carbon experiences no change in substitution, giving progressively larger differences of −3.7, −4.1 and −4.4 ppm, respectively. All of these chemical shift changes, with the possible exception of δ_C3_, appear to be caused mainly by substitution effects based on comparisons of experimental and calculated ^13^C shifts (see the following discussion).

Potential effects of conformation on ^1^H and ^13^C chemical shifts in **5**, **6** and **8**–**10** were investigated by calculating these shifts using CASPER (33, 34, 35) and comparing the calculated and experimental values. The results of these calculations, and a discussion of the results, are found in the Supporting Information (Tables S7–S8 and Figs. S27–S33).

### Qualitative analysis of inter-residue NMR spin-couplings in 3–10

Conventional and non-conventional NMR spin-coupling constants (Fig. 2) sensitive to the *ϕ* and *ψ* torsion angles in βGlcNAc-(1→4)-βMan linkages in **3**, **5-6** and **8-10**, and in the αMan-(1→3)-βMan linkages of **4** and **6**–**8** in aqueous (^2^H_2_O) solution are shown in Tables 1 and 2, respectively.

**Table 1:** Trans-*O*-Glycosidic NMR Spin-Coupling Constants^a^ Sensitive to *ϕ* and *ψ* in the βGlcNAc-(1→4)-βMan Linkages of 3, 5–6, and 8–10

compound	*ϕ-dependent J-couplings*				*ψ-dependent J-couplings*		
	^2^*J*_C1’,C4_	^2^*J*_C2’,H1’_	^3^*J*_C2’,C4_	^3^*J*_C4,H1’_	^3^*J*_C1’,C3_	^3^*J*_C1’,C5_	^3^*J*_C1’,H4_
**βGlcNAc-(1**→**4)**-βManOCH_3_ (**3**)	−2.0	+1.0	3.2	4.2	0	2.7	4.9
**βGlcNAc-(1**→**4)**-[αMan-(1→6)]-βManOCH_3_ (**5**)	−2.0	+0.8	3.1	4.1	0	2.5	4.9
**βGlcNAc-(1**→**4)**-[αMan-(1→3)]-βManOCH_3_ (**6**)	−2.0	+1.1	3.1	3.7	∼0.8	1.0	5.1
**βGlcNAc-(1**→**4)**-[αMan-(1→3;1→6)]-βManOCH_3_ (**8**)	−1.7	+1.0	3.0	3.5	∼0.7	∼0.5	5.2
**βGlcNAc-(1**→**4)**-[αMan-(1→2)-αMan-(1→6)]-βManOCH_3_ (**9**)	−1.9	+0.9	*nm*	4.2	0	2.5	*nm*
**βGlcNAc-(1**→**4)**-[αMan_4_]-βManOCH_3_ (**10**)	−1.8	+0.9	*nm*	3.5	∼0.9	0	5.5

a In Hz ± 0.1 Hz; 22 °C; in ^2^H_2_O; *nm* denotes a value that could not be measured. Values reported as 0 Hz may be 0.5 Hz or less. For each *J*-value, the primed atom resides in the βGlcNAc residue, and the unprimed atom resides in the βMan residue.

**Table 2:** Trans-*O*-Glycosidic NMR Spin-Coupling Constants^a^ Sensitive to *ϕ* and *ψ* in the αMan-(1 → 3)-βMan Linkages of **4** and **6**–**8**

compound	*ϕ-dependent J-couplings*			*ψ-dependent J-couplings*		
	^2^*J*_C1’,C3_	^3^*J*_C2’,C3_	^3^*J*_C3,H1’_	^3^*J*_C1’,C2_	^3^*J*_C1’,C4_	^3^*J*_C1’,H3_
**αMan-(1→3)**-βManOCH_3_ (**4**)^b^	−1.9	3.5	3.7	*br*	1.5	4.9
βGlcNAc-(1→4)-[**αMan-(1→3)**]-βManOCH_3_ (**6**)	−1.8	3.5	3.7	*br*	∼0.5	4.9
**αMan-(1→3)**-[αMan-(1→6)]-βManOCH_3_ (**7**)	−1.8	3.6	3.8	*br*	1.5	4.9
βGlcNAc-(1→4)-[**αMan(1→3**;1→6)]-βManOCH_3_ (**8**)	−1.6	3.5	3.6	*br*	*br*	5.1

a In Hz ± 0.1 Hz; 22 °C; in ^2^H_2_O; *br* denotes a broadened signal (*J* < 0.5 Hz).

b Couplings for **4** and **7** were taken from ref. 25.

The βGlcNAc-(1→4)-βMan linkages in **3**, **5** and **9** give essentially identical *ϕ*-dependent *J*-couplings, ^2^*J*_C1’,C4_, ^2^*J*_C2’,H1’_, ^3^*J*_C4,H1’_ and ^3^*J*_C2’,C4_ (Table 1). The observed differences between corresponding *J*-couplings (±0.1 Hz) lie within the experimental errors of the measurements. Nearly identical corresponding *ψ*-dependent *J*-couplings (^3^*J*_C1’,C4_, ^3^*J*_C1’,C3_ and ^3^*J*_C1’,C5_) are also observed for the βGlcNAc-(1→4)-βMan linkages of **3**, **5** and **9** (Table 1). These observations indicate qualitatively that the conformational properties of the βGlcNAc-(1→4)-βMan linkage are minimally affected by the addition of an αMan residue at O6 of βMan in **3** to give **5** and by the addition of an αMan-(1→2)-αMan disaccharide at O6 of βMan in **3** to give **9**.

α-(1→3)-Mannosylation at O3 of the βMan residue of **3** gives branched trisaccharide **6**. Considering only the βGlcNAc-(1→4)-βMan linkages, corresponding *ϕ*-dependent *J*-couplings ^2^*J*_C2’,H1’_, ^2^*J*_C1′,C4_ and ^3^*J*_C2′,C4_ are similar in **3** and **6**, but ^3^*J*_C4,H1′_ is 0.5 Hz smaller in **6** than in **3**. The *ψ*-dependent *J*-couplings, ^3^*J*_C1′,C3_ and ^3^*J*_C1__'__,C5_, are significantly different in **3** and **6**, with ^3^*J*_C1′,C3_ increasing by ∼0.8 Hz and ^3^*J*_C1′,C5_ decreasing by 1.7 Hz in **6** relative to **3**. These results suggest that α-mannosylation at O3 of the βMan residue of **3** alters the conformation of the βGlcNAc-(1→4)-βMan linkage significantly. Similar changes are observed when α-mannosylation occurs at both O3 and O6 of the βMan residue of **3** to give **8** and **10**.

Corresponding trans-*O*-glycosidic *J*-couplings sensitive to *ϕ* (^2^*J*_C1’,C3_, ^3^*J*_C3,H1’_, ^3^*J*_C2’,C3_) in the αMan-(1→3)-βMan linkages of disaccharide **4** and oligosaccharides **6**–**8** are virtually identical, suggesting that the bisecting βGlcNAc residue does not affect this torsion angle appreciably (Table 2). In contrast, similar but not identical corresponding *ψ*-sensitive *J*-couplings (^3^*J*_C1’,H3_, ^3^*J*_C1’,C2_, ^3^*J*_C1’,__C4_) for the αMan-(1→3)-βMan linkages in **4** and **6**–**8** were observed (Table 2). ^3^*J*_C1′,C4_ is smaller by ∼1.0 Hz in **6**–**8** relative to that in **4**, suggesting that the bisecting βGlcNAc affects *ψ*.

The qualitative treatments discussed above hinge on the assumption that the equations relating trans-*O*-glycosidic *J*-couplings to either *ϕ* or *ψ*, which were derived by density functional theory (DFT) using *in silico* disaccharides **3**^c^ and **4**^c^ for the βGlcNAc-(1→4)-βMan and αMan-(1→3)-βMan linkages, respectively, can be applied to corresponding linkages in **5**–**10**. Previous work has shown that this assumption is valid (22, 25). For example, α-mannosylation at O3 of the βMan residue of **3** to give **6** does not affect the *ϕ*- and *ψ*-sensitive equations for the βGlcNAc-(1→4)-βMan linkage significantly. Thus, a single set of equations was used in *MA’AT* analyses of *ϕ* or *ψ* in the βGlcNAc-(1→4)-βMan linkages of **3**, **5**, **6**, and **8**–**10**, and a single but different set of equations, reported previously (25), was used in *MA’AT* analyses of *ϕ* or *ψ* in the αMan-(1→3)-βMan linkages of **6**–**8**.

### MA’AT modeling of the βGlcNAc-(1 → 4)-βMan linkages in 3, 5-6 and 8-10

**Figure 4:**
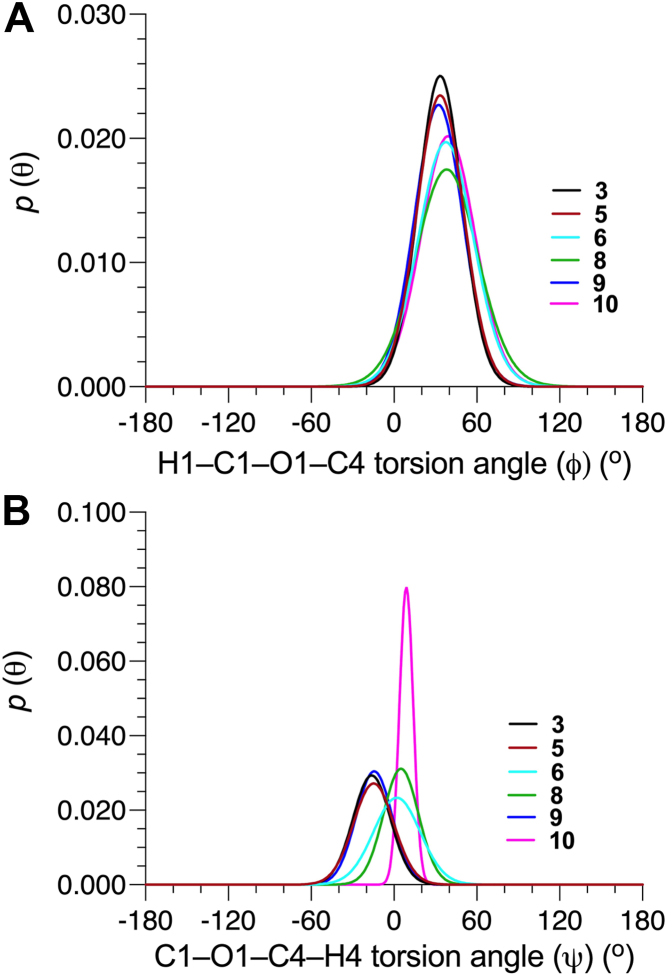
**Single-state probability distributions of *ϕ* and *ψ* for the βGlcNAc-(1→4)-βMan linkages in 3, 5-6 and 8-10 obtained from *MA'AT* analysis**. (*A*) *ϕ*. (*B*) *ψ*.

Parameterized *J*-coupling equations (eqs [S1]–[S6], Supporting Information) and experimental *J*-couplings (Table 1) were used to generate single-state models of *ϕ* and *ψ* in the βGlcNAc-(1→4)-βMan linkages of **3**, **5-6**, and **8-10** (Fig. 4). Mean *O*-glycosidic torsion angles and their circular standard deviations (CSDs), and the RMSDs for these models are shown in Table 3. The relatively small RMSDs (0.3–0.7 Hz) indicate good fits of the experimental *J*-couplings to corresponding *MA’AT* models. As expected from qualitative analyses of the *ϕ*-dependent *J*-couplings discussed above, mean *ϕ* angles (average ± STD: 33.0° ± 0.7°) and mean *ψ* angles (average ± STD: −15.1° ± 0.9°) in **3**, **5** and **9** are very similar, indicating that α-mannosylation at only O6 of the βMan residue of **3** does not significantly affect βGlcNAc-(1→4)-βMan linkage conformation. α-Mannosylation of O3 of **3** to give **6** shifts the mean value of *ϕ* from 33.4° to 37.5° (Δ = 4.1°). On the other hand, the mean value of *ψ* changes significantly from −16.2° to +2.0° for the **3**→**6** conversion, for a Δ of 18.2°. α-Mannosylation at both O3 and O6 of **3** to give **8** causes a change in the mean value of *ϕ* (Δ = 4.7°) comparable to that found for the **3**→**6** conversion and a slightly greater change (Δ = 21.2°) in the mean value of *ψ*. α-(1→2)-Mannosylation of the αMan residues of the 3- and 6-arms of **8** to give **10** produces a small change (Δ = 1.0°) in the mean value of *ϕ* and a modest change (Δ = 3.9°) in the mean value of *ψ* compared to corresponding changes found for the **3**→**8** conversion. Collectively, *MA’AT* analyses show that *ϕ* is mainly controlled by the *exo*-anomeric effect (36, 37, 38, 39, 40, 41) and that α-mannosylation of **3** at O3 has a minimal effect on its behavior. α-Mannosylation at O3, however, significantly affects *ψ*, presumably due to increased steric effects on the proximal βGlcNAc-(1→4)-βMan linkage.

**Figure 5:**
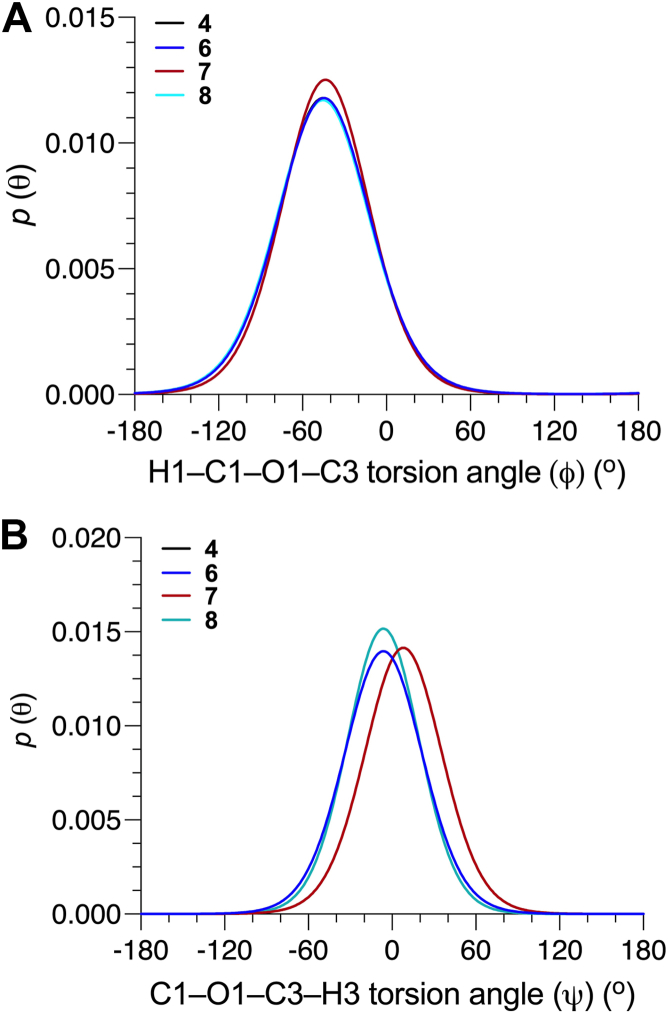
**Single-state probability distributions of *ϕ* and *ψ* for the αMan-(1→3)-βMan linkages in 4 and 6-8 determined from *MA’AT* analysis**. (*A*) *ϕ*. (*B*) *ψ*. In (*A*), the distributions for **4**, **6** and **8** overlap. In (*B*), the distributions for **4** and **7** overlap.

**Figure 6:**
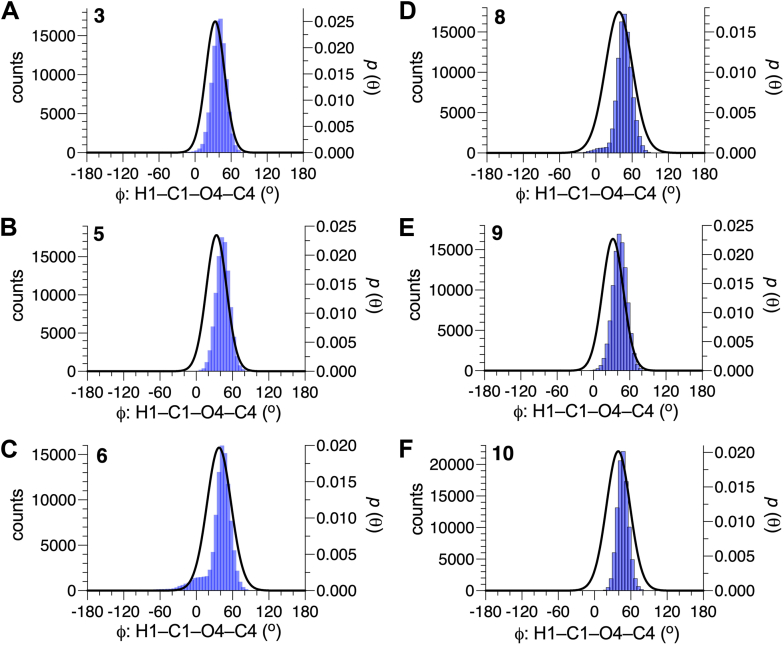
**Probability distributions of *ϕ* for the βGlcNAc-(1→4)-βMan linkages in 3, 5-6 and 8-10 obtained from aqueous 1-μs MD simulations**. MD results (*blue hatched*) are superimposed on corresponding distributions obtained from *MA’AT* analysis (*black curves*). (*A*) **3**. (*B* and *C*) **5** and **6**, (*D*–*F*) **8**-**10**. The results are summarized in Table 4. Unprimed and primed atoms have been omitted in the definitions of *ϕ* on the *x*-axis of each plot for simplicity. MD, molecular dynamics.

**Figure 7:**
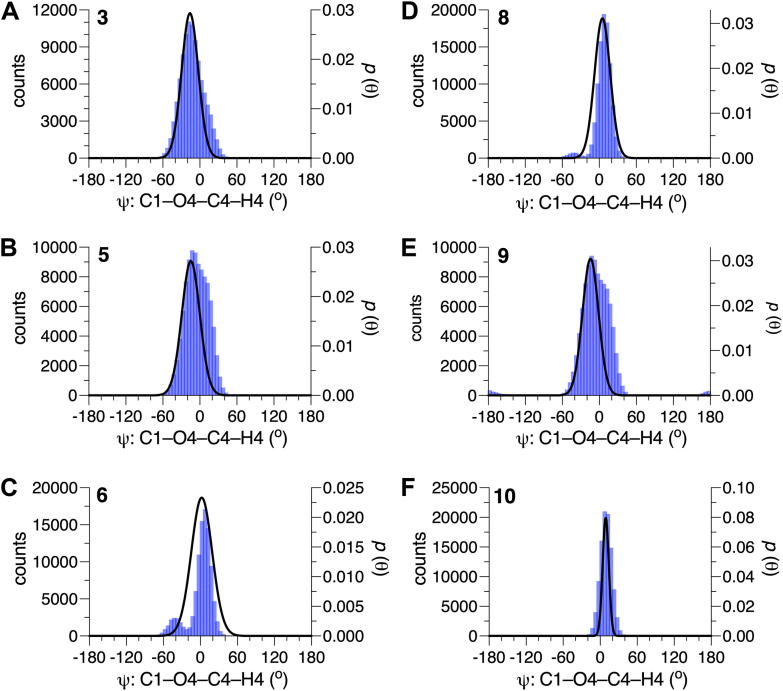
**Probability distributions of *ψ* for the βGlcNAc-(1→4)-βMan linkages in 3, 5-6 and 8-10 obtained from aqueous 1-μs MD simulations**. MD results (*blue hatched*) are superimposed on corresponding distributions obtained from *MA’AT* analysis (*black curves*). (*A*) **3**. (*B* and *C*) **5** and **6**. (*D*–*F*) **8**-**10**. The data are summarized in Table 4. Unprimed and primed atoms have been omitted in the definitions of *ψ* on the *x*-axis of each plot for simplicity. MD, molecular dynamics.

**Table 3:** Single-State von Mises Model Parameters (Mean Angles and CSDs) and RMSDs for *ϕ* and *ψ* in 3–10 Obtained from *MA’AT* Analyses

compd	torsion angle *phi* (*ϕ*)			torsion angle *psi* (*ψ*)		
	mean ± SE^a^ (^o^)	CSD^b^ ± SE (^o^)	RMSD^c^ (Hz)	mean ± SE (^o^)	CSD ± SE (^o^)	RMSD (Hz)
	βGlcNAc-(1 → 4)-βMan linkage					

**3**	33.4 ± 8.2	16.1 ± 14.6	0.48	−16.2 ± 6.4	13.7 ± 14.3	0.27
**5**	33.4 ± 8.3	17.2 ± 14.1	0.59	−14.8 ± 6.6	14.8 ± 13.4	0.33
**6**	37.5 ± 8.5	20.6 ± 14.0	0.52	2.0 ± 7.1	17.3 ± 11.0	0.47
**8**	38.1 ± 9.1	23.3 ± 13.4	0.67	5.0 ± 6.6	13.0 ± 13.4	0.66
**9**	32.2 ± 8.6^d^	17.8 ± 13.4	0.55	−14.4 ± 6.4^e^	13.2 ± 14.5	0.32
**10**	39.1 ± 8.2^d^	20.1 ± 14.7	0.67	8.9 ± 6.0	4.0 ± 24.0	0.69

	αMan-(1→3)-βMan linkage					

**4**	−45.2 ± 12.2	35.8 ± 14.9	0.01	8.0 ± 11.5	28.8 ± 9.3	0.36
**6**	−44.7 ± 12.2	35.8 ± 14.7	0.07	−6.3 ± 12.5	29.2 ± 8.7	0.70
7	−43.6 ± 10.9	33.4 ± 14.3	0.06	8.0 ± 11.5	28.8 ± 9.3	0.36
**8**	−45.4 ± 12.4	36.1 ± 14.5	0.19	−6.3 ± 11.6	26.9 ± 8.7	0.66

a SE = standard error.

b CSD = circular standard deviation.

c RMSD = root mean squared deviation.

d Fits assumed a ^3^*J*_C2’,C4_ of 3.1 Hz since this value could not be measured experimentally.

e Fit assumed a value of 5.0 Hz for ^3^*J*_C1’,H4_.

Librational averaging of *ϕ* and *ψ* was evaluated from the CSDs obtained from *MA’AT* analysis. For the βGlcNAc-(1→4)-βMan linkages, the average CSD for *ϕ* (19.2^o^ ± 2.7°) is larger than for *ψ* (12.7° ± 4.5°). The CSDs for *ϕ* increase and the CSDs for *ψ* decrease upon α-mannosylation at O3 or/and O6 of the βMan residue of **3**. For example, the CSD for *ϕ* in **3** (16.1°) increases to 23.3° in **8** (Δ = 7.2°), indicating greater flexibility in **8**. Increased steric interactions in **8** restricts the librational motion of *ψ*, indicated by a slightly smaller CSD (13.0°) in **8** than in **3**, **5** and **6** (13.7^o^ −17.3^o^). The restriction is more pronounced in **10** which gives a CSD for *ψ* of 4.0°, much smaller than values found for **3**, **5**, **6**, **8** and **9**.

### MA’AT modeling of the αMan-(1→3)-βMan linkages in **4** and **6-8**

The conformational properties of the αMan-(1→3)-βMan linkages in **4** and **7** have been studied by *MA’AT* analysis previously (25). Torsion angle *ϕ* in **4** and **7** were reinvestigated using updated *J*-coupling equations (eqs [S8]–[S10], Supporting Information), which gave smaller RMSDs than reported earlier, indicating better fits to the data (Table 3). The new equations for *ϕ* and those reported previously for *ψ* (25) (eqs [S11]–[S13], Supporting Information) were used with the experimental *J*-couplings (Table 2) to investigate the effect of a bisecting βGlcNAc on the conformational properties of the αMan-(1→3)-βMan linkages in **6** and **8**. Essentially identical mean values of *ϕ* were obtained in **4** and **6** to **8** (average ± STD: −44.7° ± 0.8°) (Fig. 5, Table 3), indicating that the bisecting βGlcNAc-(1→4)-βMan linkage in **6** and **8** exerts little effect on *ϕ*. Similar mean values of *ψ* (−6.3°) were found for the αMan-(1→3)-βMan linkages in **6** and **8**, revealing a 14.3^o^ change from corresponding values found for **4** and **7** (8.0°). These results show that the proximal bisecting βGlcNAc residue alters the conformations of αMan-(1→3)-βMan linkages significantly, and mainly through changes in *ψ*. Librational averaging of *ϕ* and *ψ* in the αMan-(1→3)-βMan linkages is similar in **4** and **6**–**8,** indicating that the bisecting βGlcNAc residue does not alter the flexibility of either linkage angle to any appreciable extent.

**Figure 8:**
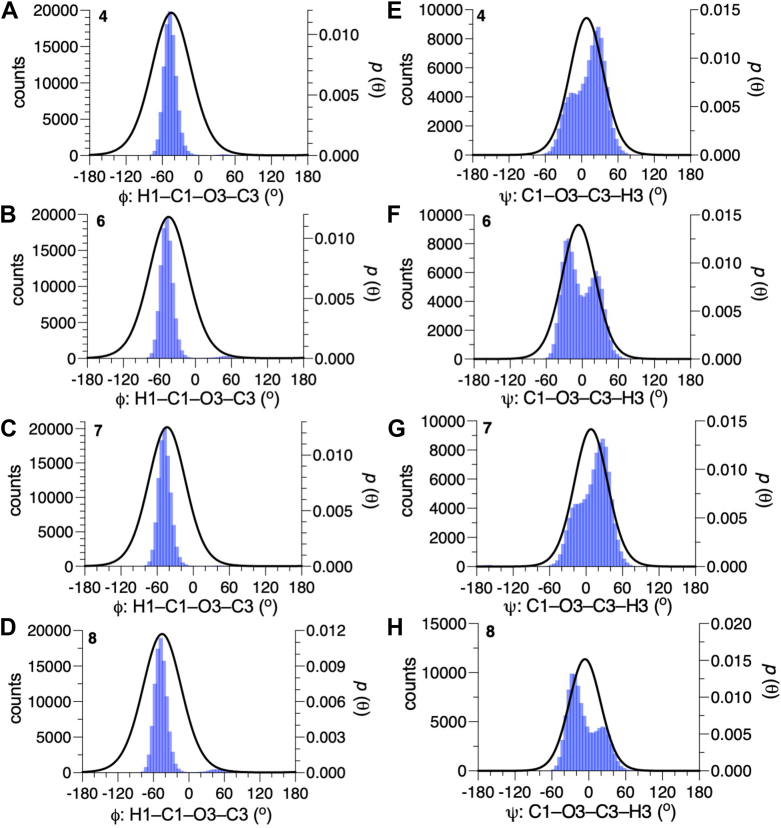
**Probability distributions of *ϕ* and *ψ* for the αMan-(1→3)-βMan linkages in 4 and 6-8 obtained from aqueous 1-μs MD simulations**. MD results (*blue hatched*) are superimposed on corresponding distributions obtained from *MA’AT* analysis (*black curves*). (*A*–*D*) *ϕ* in **4**, **6**, **7** and **8**. (*E*–*H*) *ψ* in **4**, **6**, **7** and **8**. The data are summarized in Table 4. Unprimed and primed atoms have been omitted in the definitions of *ϕ* and *ψ* on the *x*-axis of each plot for simplicity. MD, molecular dynamics.

In this work, unimodal (single-state) von Mises models were used to treat *ϕ* and *ψ*, and two parameters, the mean angle and its CSD, were obtained from these treatments. Parameter space plots generated by the *MA’AT* application (24) were used to determine the uniqueness of fit (Figs. S18 and S19, Supporting Information). Unique solutions were observed for *ϕ* in all compounds and for *ψ* in most compounds. In all cases, the global minimum gave the smallest RMSD in parameter space plots that contained two or three minima. Additional structural constraints may be incorporated in *MA’AT* analysis to reduce and/or eliminate these local minima in future work, for example, by using non-conventional ^2^*J*_C2,H3_, ^2^*J*_C4,H3_ and ^2^*J*_C2,C4_ values (28) as additional constraints on *ψ* for the αMan-(1→3)-βMan linkages where multiple minima were observed in parameter space plots.

### Behavior of linkage torsion angles ϕ and *ψ* in 3-10 determined from aqueous MD simulations

**Figure 9:**
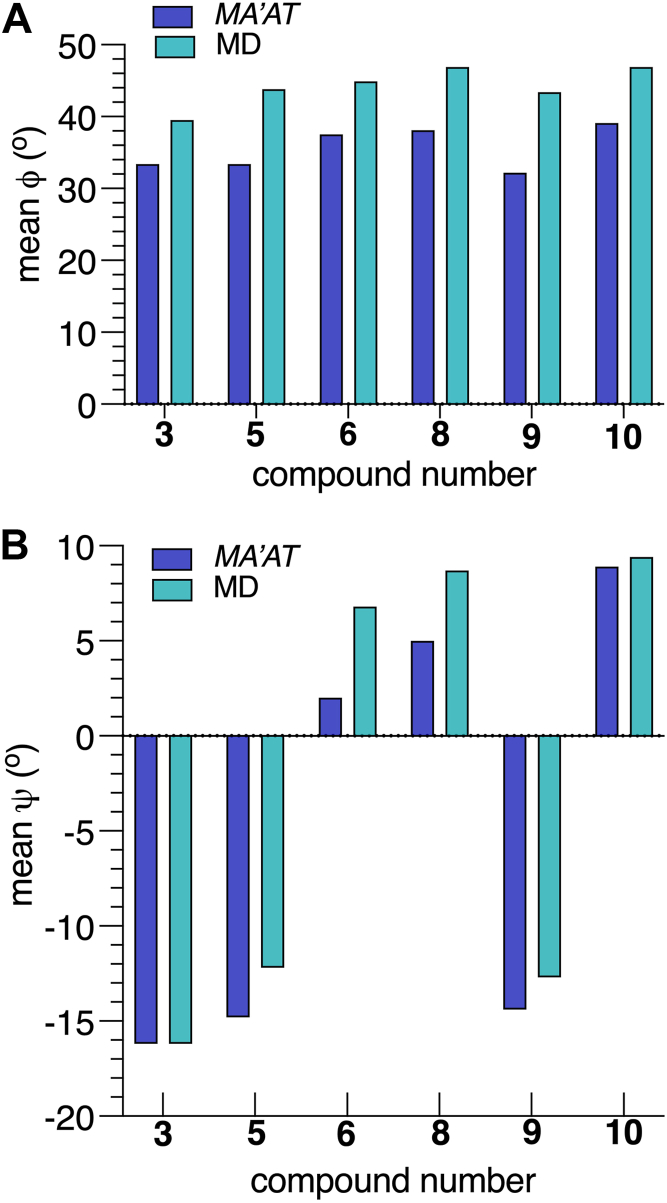
**Comparison of mean values of *ϕ* and *ψ* in the βGlcNAc-(1→4)-βMan linkages of 3, 5-6 and 8-10 determined by *MA’AT* analysis and MD**. (*A*) *ϕ*. (*B*) *ψ*. *Blue*, *MA'AT* analysis. *Green*, MD. Data were taken from Tables 3 and 4. Only MD data for the dominant conformation are shown (Mean 1 in Table 4). MD, molecular dynamics.

The solution behaviors of *ϕ* and *ψ* determined from 1-μs aqueous MD simulations are shown in Figures 6 and 7 for the βGlcNAc-(1→4)-βMan linkages in **3**, **5-6** and **8-10**, and in Figure 8 for the αMan-(1→3)-βMan linkages in **4** and **6** to **8.** The statistics from these simulations are summarized in Table 4. The MD histograms are superimposed on the corresponding probability distributions determined from *MA’AT* analysis. With the exception of **6**, the MD distributions of *ϕ* in the βGlcNAc-(1→4)-βMan linkages are unimodal (>94%), allowing direct comparisons to the probability distributions obtained from *MA’AT* analysis. Mean values of *ϕ* in βGlcNAc-(1→4)-βMan linkages obtained from *MA'AT* analysis differ from those obtained from MD by ∼9^o^, with *MA’AT* analysis giving smaller, less positive angles (Figs. 6 and 9*A*). A similar comparison of the αMan-(1→3)-βMan linkages in **4** and **6-8** (Fig. 8, *A*–*D*) shows better agreement between MD and *MA’AT*, with differences of < ∼3° and *MA’AT* analysis giving slightly less negative angles. Like the βGlcNAc-(1→4)-βMan linkages, MD predicts essentially single-state distributions of *ϕ* for the αMan-(1→3)-βMan linkages, permitting direct comparisons to the *MA’AT* results. CSDs for *ϕ* in both linkages determined by MD are consistently smaller than those determined from *MA’AT* analysis, with the average difference for the βGlcNAc-(1→4)-βMan linkages (∼8°) much smaller than found for the αMan-(1→3)-βMan linkages (∼25°). The addition of nonconventional geminal ^2^*J*_C2’.H1’_ values as additional *MA’AT* constraints on *ϕ* for the βGlcNAc-(1→4)-βMan linkages has been shown to reduce *ϕ* CSDs substantially, bringing them in better alignment with CSDs obtained by MD. The use of this additional constraint is possible due to constrained rotational averaging about the C2–N2 exocyclic bond of the *N*-acetyl side-chains of GlcNAc residues (27, 42). For the αMan-(1→3)-βMan linkages, this additional constraint is not available due to insufficient prior knowledge of the rotational properties of the exocyclic C2–O2 bond in αMan residues in solution, leading to much larger *MA’AT*-determined CSDs that reflect solution behavior inaccurately. In general, the MD results show that structural context exerts a minimal effect on *ϕ*, consistent with *MA’AT* results.

**Figure 10:**
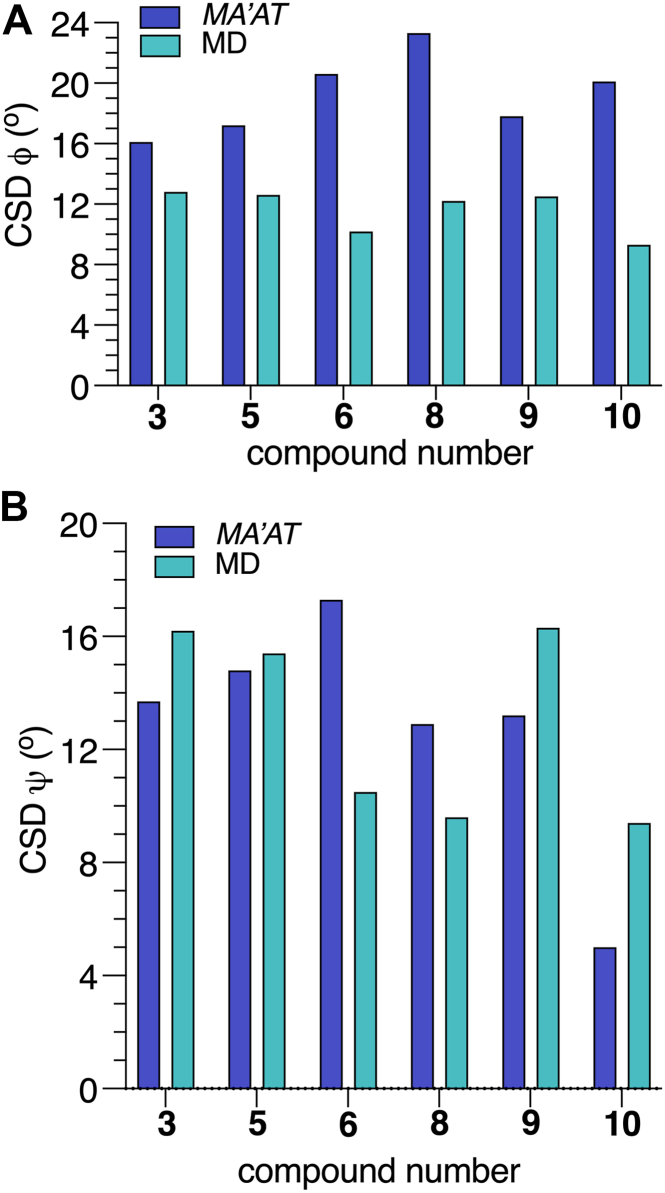
**Comparison of the CSDs for *ϕ* and *ψ* in the βGlcNAc-(1→4)-βMan linkages of 3, 5-6 and 8-10 determined by *MA’AT* analysis and MD simulation.** (*A*) *ϕ*. (*B*) *ψ*. *Blue*, *MA'AT* analysis. *Green*, MD. Data were taken from Tables 3 and 4. Only MD data for the dominant conformation are shown (Mean 1 in Table 4). MD, molecular dynamics.

**Figure 11:**
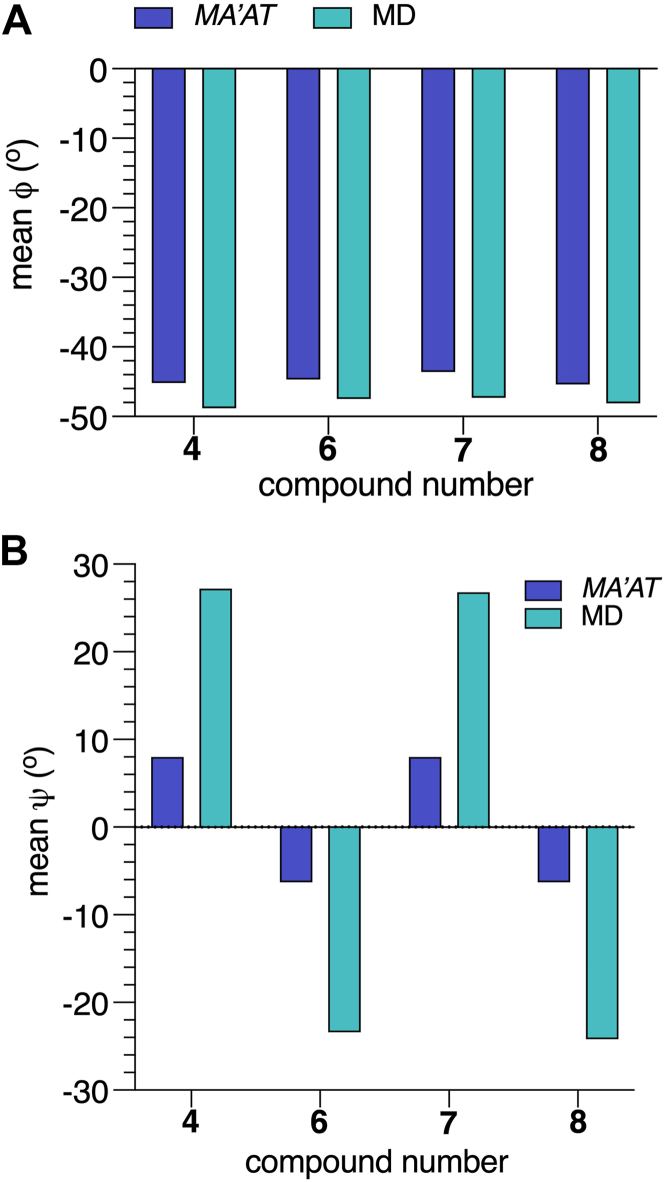
**Comparison of mean values of *ϕ* and *ψ* in the αMan-(1→3)-βMan linkages of 4 and 6-8 determined by *MA’AT* analysis and MD simulation.** (*A*) *ϕ*. (*B*) *ψ*. *Blue*, *MA'AT* analysis. *Green*, MD. Data were taken from Tables 3 and 4. Only MD data for the dominant population are shown (Mean one in Table 4). MD, molecular dynamics.

**Table 4:** Statistics on *O*-Glycosidic Torsion Angles *ϕ* and *ψ* in βGlcNAc-(1→4)-βMan and αMan-(1→3)-βMan Linkages in 3, 5–6 and 8–10 Obtained from Aqueous 1-μs Molecular Dynamics Simulations

compd	*phi (ϕ*)						*psi (ψ*)					
	Mean 1 (^o^)	Mean 2 (^o^)	CSD^a^ 1 (^o^)	CSD 2 (^o^)	Peak 1 (%)	Peak 2 (%)	Mean 1 (^o^)	Mean 2 (^o^)	CSD 1 (^o^)	CSD 2 (^o^)	Peak 1 (%)	Peak 2 (%)
	βGlcNAc-(1→4)-βMan linkage											

**3**	39.5		12.8		100		−16.2	16.3	16.2	10.1	88.3	11.4
**5**	43.8		12.6		100		−12.2	15	15.4	10.2	73.6	26.1
**6**	44.9	16.8	10.2	36.3	77.2	22.8	6.8	−41.3	10.5	10.9	86.7	12.8
**8**	46.9	6.8	12.2	28.1	94.1	5.9	8.7	−41.6	9.6	12.2	95	4.3
**9**	43.4		12.5		100		−12.7	16	16.3	10.4	74.6	24.4
**10**	46.9		9.3		100		9.4		9.4		100	

	αMan-(1→3)-βMan linkage											

**4**	−48.8		10.9		99.0		27.2	−18.3	15.4	16.4	65.8	34.2
**6**	−47.5	45.2	10.6	12.2	98.0	2.0	−23.4	20.9	12.7	16.0	52.4	47.6
**7**	−47.3		10.6		99.1		26.8	−18.5	16.8	15.5	69.5	29.9
**8**	−48.1	45.0	10.5	16.0	96.1	3.9	−24.2	19.3	12.8	17.5	61.1	38.9
**10**	−47.1		10.1		99.1		−22.2	−2.4	10.5	19.0	73.9	26.1

a CSD = circular standard deviation. Peaks with populations < 1% are not shown.

**Figure 12:**
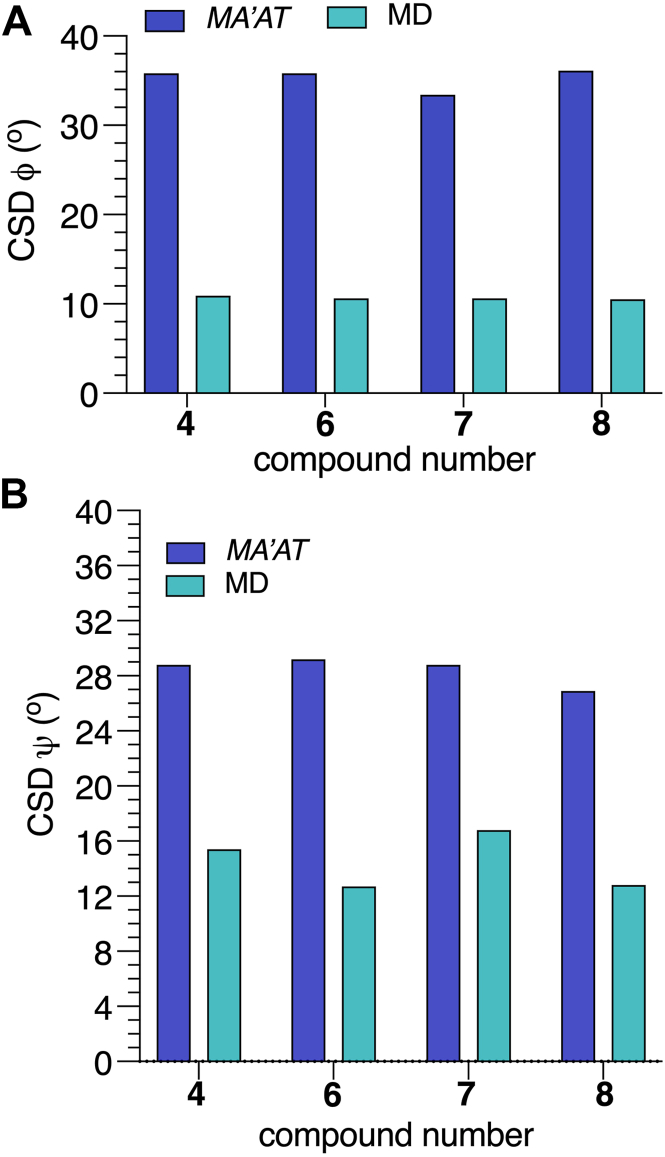
**Comparison of the CSDs for *ϕψ* in the αMan-(1→3)-βMan linkages of 4 and 6-8 determined by *MA’AT* analysis and MD simulation**. (*A*) *ϕ*. (*B*) *ψ*. *Blue*, *MA'AT* analysis. *Green*, MD. Data were taken from Tables 3 and 4. Only MD data for the dominant population are shown (Mean one in Table 4). MD, molecular dynamics.

The distributions of *ψ* in the βGlcNAc-(1→4)-βMan linkages determined by MD simulation (Fig. 7) are not exclusively single-state (*e*.*g*., *ψ* in **5**, **6** and **9**), making comparisons to those determined by *MA’AT* analysis less straightforward. This problem is reduced if comparisons are made between the *MA’AT* model and the MD model for the dominant conformational state. This comparison (Fig. 9*B*) shows that the mean values determined by both methods are similar (within ∼4° or less of each other) and, importantly, exhibit similar trends. A comparison of *MA’AT*- and MD-determined CSDs (Fig. 10*B*) shows that both methods give similar, although not identical, values and trends. The latter agreement is noteworthy for **10** where both *MA’AT* and MD indicate less librational averaging about *ψ* than found for **3**, **5** and **9**. The largest discrepancy between *MA’AT-* and MD-determined CSDs is observed for **6**, as expected, since MD produces a two-state model (Fig. 7*C*) whereas *MA’AT* produces a single-state model that includes both the major and minor MD populations. On the other hand, MD predicts that **6** (major population) and **10** have similar CSDs (Fig. 10*B*), whereas *MA’AT* analysis indicates much less librational averaging in **10** than in **6**. The latter behavior appears more likely than the former.

**Figure 13:**
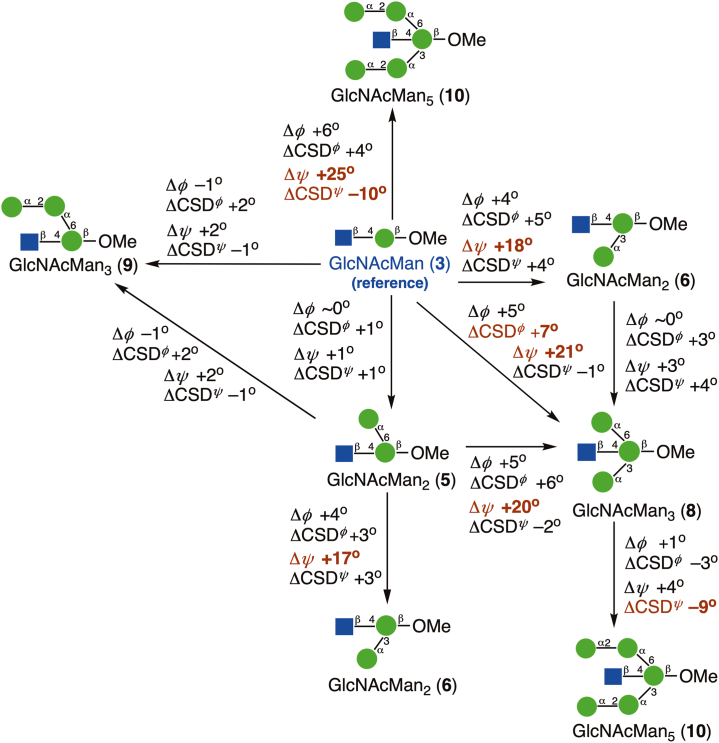
**Summary of context effects on the**β**GlcNAc-(1→4)-**β**Man linkages in oligosaccharides 5, 6, and 8-10 determined by *MA’AT* analysis**. Disaccharide **3** was the reference state (*shown in blue*). The effects are largely confined to mean values and CSDs of the ψ torsion angle. Significant changes are highlighted in *red*.

Comparisons of mean values of *ϕ* and *ψ* in **4** and **6-8** obtained by *MA’AT* analysis and MD for the αMan-(1→3)-βMan linkages are shown in Figure 11. Differences in the values of *ϕ* are small, in contrast to *ψ* where *MA’AT* analysis gives smaller absolute values of this angle. Importantly, however, the relative changes in *ψ* as **4** is converted to **6-8** are maintained in the two data sets. Given the high reliability of *MA’AT* analysis in determining mean values of molecular torsion angles in solution (24), it is likely that the MD results are in error.

**Figure 14:**
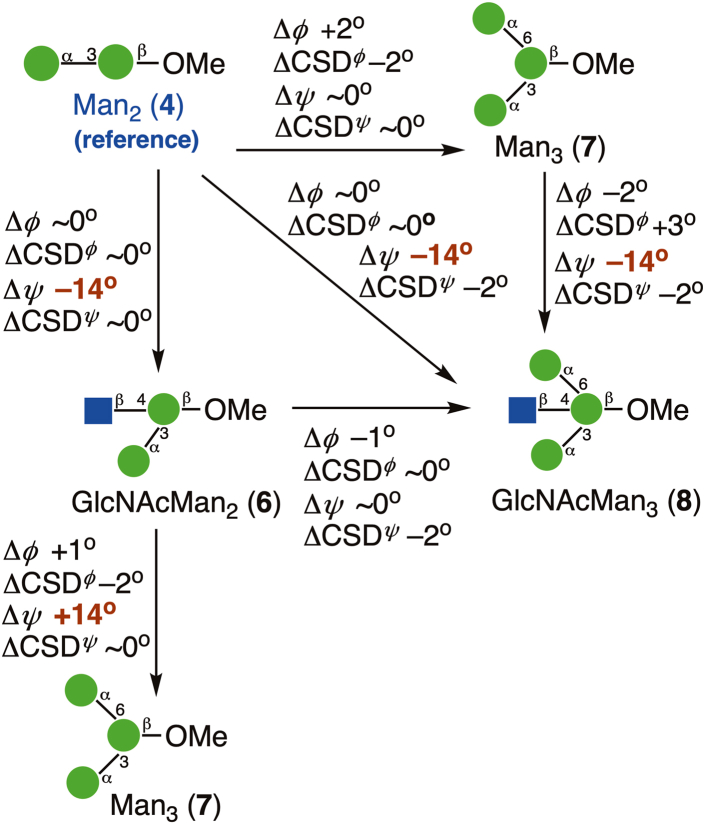
**Summary of context effects on the**α**Man-(1→3)-**β**Man linkages in oligosaccharides 6-8 determined by *MA’AT* analysis**. Disaccharide **4** was the reference state (*shown in blue*). The effects on mean torsion angles and CSDs are small except for that on the mean value of ψ caused by insertion of a proximal βGlcNAc-(1→4)-βMan linkage (values highlighted in *red*).

A comparison of the CSDs for *ϕ* and *ψ* for the αMan-(1→3)-βMan linkages obtained from *MA’AT* analysis and MD reveals significant differences for *ϕ*, with *MA’AT*-determined values much greater than MD values (Fig. 12*A*). This disparity has been explained in recent work (27) and is attributed to the use of a geminal ^2^*J*_COC_ value as a constraint in the analysis. This source of error was avoided in the treatment of *ϕ* in the βGlcNAc-(1→4)-βMan linkages, resulting in better agreement between the CSDs determined by the two methods (Fig. 10*A*). The large discrepancy between the CSDs for *ψ* in these linkages determined by *MA’AT* analysis and MD (Fig. 12*B*) is an artifact caused by the manner in which the CSDs were calculated from the MD data. As shown in Figure 10, *E*–*H*, MD gave bimodal distributions of *ψ* whereas *MA’AT* analysis gave unimodal models that include both MD populations. Since the CSDs determined from the MD data pertain only to the dominant population, they will be smaller than those determined by *MA’AT* analysis.

**Figure 15:**
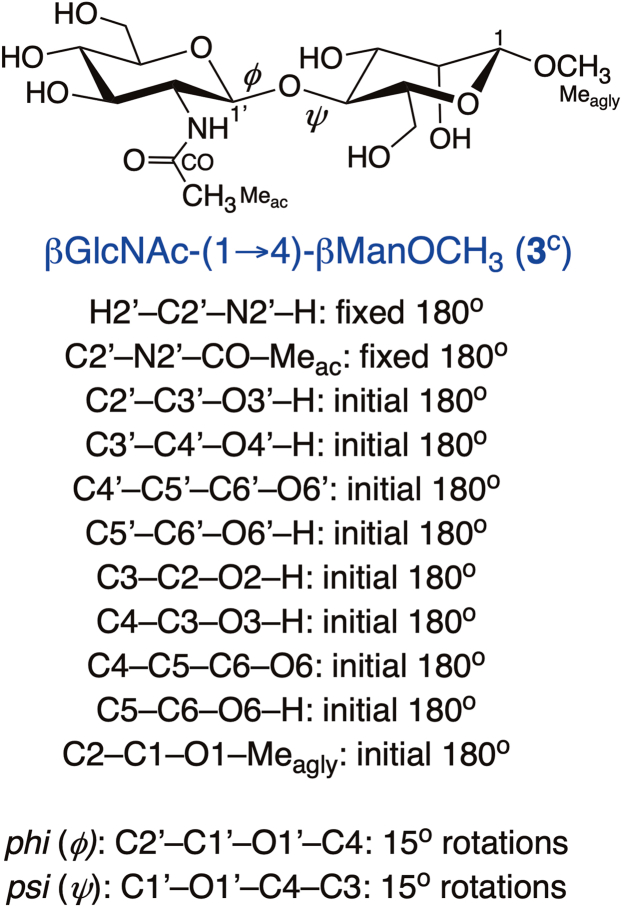
**Structural constraints used in DFT calculations on 3^c^**. Atom numbering (anomeric carbons) and definitions of exocyclic methyl and carbonyl carbons are shown.

In this work, multi-state probability distributions of *ϕ* and *ψ* predicted from MD simulations (*e*.*g*., Fig. 8*F*) could not be validated by *MA'AT* analysis due to the limited number of experimental constraints used in the analyses (Fig. 2), which restricts the analyses to unimodal models. RMSDs back-calculated from multi-state MD models (Tables S5 and S6, Supporting Information) were similar to those determined for unimodal *MA’AT* models, preventing their use in determining which model is more accurate. Additional experimental constraints provided by nonconventional *J*-couplings (27), NOEs (43, 44) and/or residual dipolar couplings (45, 46, 47) may allow multi-state MD models to be tested by *MA'AT* analysis in future work.

## Discussion

Current NMR methods to determine the conformational properties of oligosaccharides in solution involve the measurement and interpretation of ^1^H and ^13^C chemical shifts, ^1^H-^1^H NOEs, trans-glycosidic ^3^*J*_COCH_ values, and/or residual ^13^C-^1^H residual dipolar coupling constants. None of these experimental parameters, either alone or in combination, provide explicit models of *O*-glycosidic linkages in oligosaccharides comparable to those provided by MD simulation. With the exception of ^3^*J*_COCH_ values, these NMR parameters are not C–O bond-specific in that, for a linkage comprised of two C–O bonds, the torsional properties of each cannot be determined separately. For example, changes in inter-residue ^1^H-^1^H internuclear distances can be attributed to rotation of one or both C–O bonds of the linkage. Consequently, structural interpretations of experimental NMR parameters are qualitative or at best semi-quantitative. To overcome this limitation, experimental NMR parameters are tested for consistency with MD models, resulting in models that are strongly biased by MD. Few if any options are available to test systematically whether other MD models are consistent with the experimental data.

This laboratory has been pursuing the development of an NMR approach to *O*-glycosidic linkage conformational analysis that addresses the issues discussed above. *MA’AT* analysis (23, 24) uses multiple, redundant NMR *J*-couplings and circular statistics to calculate probability distributions of molecular torsion angles in solution comparable to those obtained by MD. Based on work to date, this method provides reliable mean values of molecular torsion angles and in many but not all cases, information on their librational properties through the calculation of CSDs. The method is not only applicable to glycosidic linkages but in principle to any conformational element for which multiple redundant *J*-couplings (*i*.*e*., those that have a strong primary dependence on the same torsion angle) are available, are measurable, and can be reliably parameterized. Examples of recent *MA’AT* applications include the characterization of *O*-acetyl side-chain (48), *N*-acetyl side-chain (42), exocyclic hydroxymethyl group side-chain (49), peptide backbone (50), and furanose ring (51) conformation.

As reported here, *MA’AT* analysis independently provides probability distributions of molecular torsion angles in solution that can be superimposed on corresponding distributions obtained by MD. This ability allows determinations of explicit conformational models exclusively from experimental data. *MA’AT* analysis also provides a unique opportunity to test and validate conformational models obtained by MD.

In the context of *O*-glycosidic linkage conformation, access to homonuclear ^13^C-^13^C spin-couplings is integral to *MA’AT* analysis, requiring access to ^13^C-labeled compounds, preferably singly- or multiply-labeled (52). While this requirement might be viewed as a limitation, robust chemical and chemo-enzymatic methods are available to prepare a wide range of saccharide building blocks labeled with ^13^C, ^15^N and/or ^2^H (49), and methods to assemble them into oligosaccharides have improved enormously over the past few decades (53).

The high precision of the *MA’AT* method makes it ideally suited to studies of context effects on conformational preference. As reported here, *MA’AT* analysis detects changes in conformational preference with greater sensitivity than afforded by other NMR-based methods. Small changes in mean molecular torsion angles are detected, as well as the effects of structural context on the librational averaging of these angles. No other experimental method provides this level of structural detail independent of computational inputs.

This work demonstrates the feasibility of inserting ^13^C-labels into relatively large oligosaccharides, in the present case up to hexasaccharides. Attention to relative hydroxyl group reactivity during chemical glycosylation (54, 55), and the location of label insertion in the overall synthetic scheme (22), are important factors when choosing synthetic routes. For example, starting from disaccharide acceptor **I** containing a βGlcNAc-(1→4)-βMan linkage (Scheme S2, Supporting Information), the addition of a single αMan residue at O6 of the βMan residue to give **5**, or the addition of αMan residues at both O3 and O6 of the βMan residue to give **8**, could be achieved in the same reaction mixture. Attempts to introduce a single αMan residue at O3 of the βMan residue of **I** to give **6** were unsuccessful, probably due to interference from potential hydrogen bonding between OH3 of the βMan residue and O5 of the βGlcNAc residue in **I**. To prepare **6**, a separate route was required in which the αMan-(1→3)-βMan linkage was introduced first, followed by the βGlcNAc-(1→4)-βMan linkage (Scheme S3, Supporting Information). The chemical routes (Schemes S1–S4, Supporting Information) were chosen to be compatible with selective ^13^C-labeling from the standpoint of simplicity, reliability, and yield. The selection of the labeled carbons was based on optimizing measurements of *J*_CH_ and *J*_CC_ values across *O*-glycosidic linkages and on cost efficiencies (22).

*MA’AT* analysis of the βGlcNAc-(1→4)-βMan and αMan-(1→3)-βMan linkages in oligosaccharides **3-10** revealed that context effects on the conformational preferences of *ϕ* in both linkages are small, using disaccharides **3** and **4**, respectively, as reference states (reference states contain “isolated” linkages devoid of context effects). The range of mean *ϕ* values in the former was 7^o^ (Fig. 4) and that in the latter was 2^o^ (Fig. 5). These results confirm that *ϕ*, which is dictated in solution largely although not exclusively by stereoelectronic effects (36, 37, 38, 39, 40, 41), is difficult to perturb and minimally affected by proximal structural change. This conclusion based on *MA’AT* analysis is consistent with MD results, the latter showing only small effects on mean *ϕ* values in **5-10**. MD gives absolute mean values of *ϕ* for αMan-(1→3)-βMan linkages in good agreement with those obtained from *MA’AT* analysis, but the absolute mean values of *ϕ* for the βGlcNAc-(1→4)-βMan linkages obtained by MD differ significantly from those obtained by *MA’AT*, suggesting that the *GLYCAM06* force field may require modification to treat these angles accurately.

Context effects on *ψ* are significant for the βGlcNAc-(1→4)-βMan and αMan-(1→3)-βMan linkages, with larger effects on average found for the former in terms of mean angles and CSDs (Figs. 13 and 14). For the latter linkages, mean values of *ψ* are affected when a proximal βGlcNAc-(1→4) linkage is introduced but not when an αMan-(1→6) linkage is introduced (to give **7**) into reference disaccharide **4**, but CSDs are largely context-independent within the group of compounds examined. Adding 3- and 6-arm αMan residues to the βMan residue of **3** significantly affects the mean value of *ψ* in the βGlcNAc-(1→4)-βMan linkage, and the CSD in **10** is notably reduced. Critical to the aims of this work, predictions made from *MA’AT* analyses of *ψ* are in good but not quantitative agreement with those obtained by MD in that similar trends in mean values and librational behaviors are indicated by both methods.

Conformational studies of **7** and **8** have been reported previously, and include an experimental NMR study by Hanashima *et al*. (19) and a molecular dynamics study by Kim *et al*. (56). The former NMR study provided qualitative values of *ϕ* and *ψ* for the βGlcNAc-(1→4)-βMan and/or αMan-(1→3)-βMan linkages in **7** and **8** based largely on ^1^H-^1^H NOEs and trans-*O*-glycosidic ^3^*J*_COCH_ values. For the βGlcNAc-(1→4)-βMan linkage in **8**, values of *ϕ* were reported to be −76 – −83° based on a qualitative analysis of ^3^*J*_H1,C4_ and –90° based on an interpretation of ^1^H-^1^H NOEs, while torsion angle *ψ* was not reported. The reported *ϕ* angles are similar to those reported here (Table 3) after correcting for different definitions of the angles (the former translate into 30° – 44° when the definitions used in this report are applied). For the αMan-(1→3)-βMan linkages, *ϕ* was reported to be +72^o^ in **7** and **8** (or −48°, similar to mean values shown in Table 3), and *ψ* was reported to be either −114° or −96° (6° or 24°, which approximate those shown in Table 3). In general, and importantly, the conformational assignments made in this prior work based on traditional NMR approaches were qualitative in nature and insufficient to draw firm conclusions on preferred linkage conformation in solution and on the effects of structural context.

The aqueous MD study (56) (80-ns simulations using *GLYCAM06*) indicated that the αMan-(1→3)-βMan linkage in **7** preferred a 76° ± 15° angle for *ϕ* (−44°) (values in parentheses are converted values for comparison to this work) and a −107° ± 25° angle for *ψ* (13°), whereas the corresponding angles in **8** were 77° ± 20° (−43°) and −123^o^ ± 22° (–3°) indicating little change in *ϕ* but an ∼16° change in *ψ* upon introduction of the βGlcNAc-(1→4)-βMan linkage. The *ϕ* angles are similar to those reported in Table 4, but those for *ψ* differ, presumably due to the manner in which the bimodal nature of the *ψ* distribution was treated to give average angles. For the βGlcNAc-(1→4)-βMan linkage in **8**, two conformers were detected having *ϕ*/*ψ* values of −115° ± 12°/78° ± 11° (5^o^/−42^o^) and −69° ± 12°/126° ± 8° (51°/6°), with the latter conformer highly dominant. The results for the dominant form are similar to those reported here, where both *ϕ* and *ψ* in **8** are essentially single-state and correspond to a 47°/9° conformer (Table 4).

In this work, the effects of structural context on the αMan-(1→6) linkages in **5** and **7-10** were not examined. This problem involves the characterization of *ω*, the exocyclic C4–C5–C6–O6 torsion angle. Recent work has shown that multi-state modeling of *ω* using *MA’AT* analysis gives reliable unbiased two-state models in the simple monosaccharide, methyl β-D-galactopyranoside (49). However, similar modeling of *ω* in methyl β-D-glucopyranoside was less definitive, possibly because von Mises probability distributions of *ω* for the two dominant states, *gg* and *gt*, do not accurately capture the actual solution behavior of *ω*. Nevertheless, groundwork has been laid for *ω* analysis with *MA’AT* to enable quantitative treatments of (1→6) *O*-glycosidic linkages in future work.

## Experimental procedures

### Preparation and structural characterization of unlabeled and ^13^C-labeled 3, 5-6 and 8-10

Synthetic methods used to prepare unlabeled and ^13^C-labeled **3**, **5**-**6** and **8**-**10**, and their structural characterization by NMR spectroscopy and mass spectrometry, are described in the Supporting Information. Sixteen (16) ^13^C isotopomers of **3**, **5**-**6** and **8**-**10** were prepared, either singly- or doubly-labeled with ^13^C, as summarized in Figure 3 and discussed in detail in the Supporting Information.

### NMR spectroscopy

High-resolution 1D ^1^H and ^13^C{^1^H} NMR spectra were obtained using 5-mm NMR tubes on a 600-MHz FT-NMR spectrometer equipped with a 5-mm ^1^H-^19^ F/^15^N-^31^P AutoX dual broadband probe or on an 800-MHz FT-NMR spectrometer equipped with a 5-mm inverse triple resonance (TCI) ^1^H/^13^ C/^15^N probe. NMR spectra of intermediates were collected in CDCl_3_ at 22 °C. 2D ^1^H-^1^H gCOSY (57) and ^13^C-^1^H gHSQC (58) spectra were used to confirm ^1^H and ^13^C chemical shift assignments for the synthetic intermediates. ^1^H and ^13^C Chemical shifts were referenced internally to CHCl_3_. Representative ^1^H and ^13^C{^1^H} NMR spectra of synthetic intermediates are shown in Figures S1–S10 in the Supporting Information.

NMR spectra of final unlabeled and ^13^C-labeled oligosaccharides were obtained in ^2^H_2_O at 22 °C and FIDs were processed to optimize both resolution and spectral S/N. ^1^H NMR spectra had digital resolutions of ∼0.02 Hz/pt and ^13^C{^1^H} NMR spectra had digital resolutions of ∼0.05 Hz/pt. ^1^H and ^13^C Chemical shifts (in ppm) were referenced externally to sodium 4,4-dimethyl-4-silapentane-1-sulfonate (DSS). 2D ^1^H-^1^H gCOSY (57) and ^13^C-^1^H gHSQC (58) spectra were used to confirm the ^1^H and ^13^C chemical shift assignments. For long-range ^*n*^*J*_CH_ couplings across *O*-glycosidic linkages, 2D ^13^ C-^1^H *J*-HMBC spectra (59) were obtained with scaling factors of 10 to 50, and a two-fold low-pass *J*-filter was applied to suppress ^1^*J*_CH_ values. Non-first-order effects were minimal in 1D ^13^ C{^1^H} NMR spectra of ^13^C-labeled compounds, allowing homonuclear *J*_CC_ values to be measured directly from the observed signal splittings. For measurements of trans-*O*-glycosidic heteronuclear ^3^*J*_COCH_ values, attention was paid to non-first-order effects in 1D ^1^H NMR spectra that might affect their determination directly from signal splittings. ^1^H NMR spectra were collected at different spectrometer frequencies and spectral simulation (Bruker *TopSpin* 3.6.4) (60) was used to simulate the spectra from which accurate ^3^*J*_COCH_ values were obtained. Trans-*O*-glycosidic ^3^*J*_COCH_ values were also obtained from 2D NMR spectra of compounds at natural abundance when signal overlap prevented ^3^*J*_COCH_ measurement from signal splittings in 1D ^1^H spectra.

### Mass spectrometry

High-resolution mass spectra were obtained on a BRUKER micrOTOF-Q II instrument with an ESI source. The dry heater was set at 180 °C and the nebulizer was set at 0.4 Bar. The capillary voltage was 4.5 kV and the end plate offset was −0.5 kV. Full MS scans were collected over a range of 50 to 2500 *m*/*z*.

### Calculational methods

#### Selection and geometric optimization of model compounds

Fully substituted *in silico* structure **3**^c^ (the superscript “c” denotes an *in silico* structure) (Fig. 15) was chosen for theoretical studies of *J*-couplings. DFT calculations were conducted within *Gaussian*16 (61) using the B3LYP functional (62, 63) and 6-31G∗ basis set (64) for geometric optimization. Initial exocyclic torsion angle constraints in **3**^c^ were set as shown in Figure 15. The internal β-(1→4) linkage in **3**^c^ is characterized by two torsion angles, C2′–C1′–O1′–C4 (*ϕ*) and C1′–O1′–C4–C3 (*ψ*), each of which was rotated systematically in 15° increments through 360° to give a 24 x 24 matrix of optimized structures. All remaining geometric parameters were optimized. The calculations included the effects of solvent water, which were treated using the Self-Consistent Reaction Field (SCRF) (65) and the Integral Equation Formalism (polarizable continuum) model (IEFPCM) (66) as implemented in *Gaussian*16.

#### Density functional theory calculations of ^1^H-^1^H, ^13^C-^1^H and ^13^C-^13^C NMR spin-coupling constants in **3**^c^

*J*_HH_, *J*_CH_ and *J*_CC_ values were calculated in **3**^c^ using *Gaussian*16 (58) and DFT (B3LYP functional) (62, 63). The Fermi contact (67, 68, 69), diamagnetic and paramagnetic spin-orbit, and spin-dipole terms (67) were recovered using a [5s2p1d|3s1p] basis set (70, 71), and raw (unscaled) calculated *J*-couplings are reported. The DFT calculations of *J*-couplings included the effects of solvent water, which were treated using the Self-Consistent Reaction Field (SCRF) (65) and the Integral Equation Formalism (polarizable continuum) model (IEFPCM) (66) as implemented in *Gaussian*16.

### Parameterization of NMR spin-coupling equations

All geometrically-optimized conformers of **3**^c^ were inspected to ensure that structurally distorted structures were not used in equation parameterization, including the use of a 10-kcal/mol energy cutoff and an aldohexopyranosyl ring puckering filter. Cremer−Pople puckering parameters (72) were calculated from the DFT-generated Cartesian coordinates of each structure and a *θ* value of 35° was used as the cutoff (22, 25). Equations relating DFT-calculated *J*-couplings to either *ϕ* or *ψ* were parameterized using R. The goodness-of-fit of each equation is reported as a RMSD. To reduce the influence of *ψ* on *ϕ* dependent couplings, equations for *ϕ* were parameterized using a subset of the data wherein only *ψ* conformations indicated by both *MA’AT* analysis and MD simulation were used in the parameterization (26, 27). For βGlcNAc-(1→4)-βMan, the data set for parameterizing equations was limited to *ψ* values of −60° to 45° and for αMan-(1→3)-βMan *ψ* values of −30^o^ to 30^o^. Linkage torsion angles C2′–C1′–O1′–C4 (*ϕ*) and C1′–O1′–C4–C3 (*ψ*), which were rotated in the DFT calculations, were converted to the conventional definitions of *ϕ* (H1′–C1′–O1′–C4) and *ψ* (C1′–O1′–C4–H3) when plots of the *J*-coupling dependencies were generated (Figs. S16 and S17, Supporting Information).

### MA’AT analysis

DFT-Parameterized equations for *J*-couplings that depend on *ϕ* and *ψ* in the αMan-(1→3)-βMan linkages of **4**, **6-8** and **10** were obtained from previous work (25). These equations and those reported herein for *ϕ* and *ψ* in the βGlcNAc-(1→4)-βMan linkage was used in an in-house statistical software package, *MA’AT* (24), with each linkage torsion angle modeled as a von Mises single-state distribution. Monte Carlo methods were used to generate model parameters, and least squares methods were used to minimize the RMSD between the experimental and calculated *J*-couplings. Each analysis gave two fitting parameters, the mean torsion angle and the CSD of the angle, which reflects the degree of librational motion about the mean angle. The current version of *MA’AT* can be accessed online (https://rmeredit.shinyapps.io/maat24/) (last accessed: 11/20/25) and a User’s Manual is available on the software’s web page.

### Aqueous molecular dynamics simulations

Initial structures of **3**^c^–**10**^c^ were built using the Carbohydrate Builder module available at the GLYCAM website (http://www.glycam.org) (73). The GLYCAM06 (74) (version j) force field was employed in all simulations. The saccharides were solvated with TIP3P (75) water using a 12 Å buffer in a cubic box, using the LEaP module in the AMBER14 software package (76) (ambermd.org). Energy minimizations for the solvated di- and oligosaccharides were performed separately under constant volume (500 steps steepest descent, followed by 24,500 steps of conjugate-gradient minimization). Each system was subsequently heated to 300 K over a period of 50 ps, followed by equilibration at 300K for a further 0.5 ns using the nPT condition, with the Berendsen thermostat (77) for temperature control. All covalent bonds involving hydrogen atoms were constrained using the SHAKE algorithm (78), allowing a simulation time step of 2 fs throughout the simulation. After equilibration, production simulations were carried out with the GPU implementation (79) of the PMEMD.MPI module, and trajectory frames were collected every 1 ps for a total of 1 μs. One to four nonbonded interactions were not scaled (80), and a nonbonded cut-off of 8 Å was applied to van der Waals interactions, with long-range electrostatics treated with the particle mesh Ewald approximation. The output of each simulation was imported into *Prism* (81) for visualization.

## Supporting information

This article contains supporting information (61, 76).

## Conflict of interest

The authors declare that they have no conflicts of interest with the contents of this article.
