# Supplementary material for: Bisecting βGlcNAc: MA’AT analysis of 13C-labeled oligosaccharides containing βGlcNAc-(1→4)-βMan O-glycosidic linkages
Source: J Biol Chem. 2025 Dec 23;302(3):111108. doi: 10.1016/j.jbc.2025.111108 (PMC12945514; doi:10.1016/j.jbc.2025.111108)
Supplement: Supplementary Material 1 [file mmc1.pdf]

Supporting Information

Bisecting βGlcNAc: *MA'AT* Analysis of ^{13}C-Labeled Oligosaccharides Containing βGlcNAc-(1→4)-βMan *O*-Glycosidic Linkages

Wenhui Zhang,[1,2] Reagan Meredith,[1,3] Jieye Lin,[1,4] Mi-Kyung Yoon,[1,2] Ian Carmichael,[1,5] and Anthony S. Serianni[1]*

[1]Department of Chemistry and Biochemistry, University of Notre Dame, Notre Dame, IN 46556-5670 USA; [2]Omicron Biochemicals, Inc., South Bend, IN 46617-2701 USA; [3]Texas Biomedical Research Institute, San Antonio, TX 78245-0549 USA; [4]Department of Biological Chemistry, UCLA, Los Angeles, CA 90095-1737 USA; [5]Radiation Laboratory, University of Notre Dame, Notre Dame, IN 46556-5670 USA

Corresponding author: aseriann@nd.edu

Table of Contents

1. Preparation of disaccharide **3** and oligosaccharides **5**, **6**, **8**, **9** and **10** (Schemes S1–S4) S3
2. Table S1. ^{1}H-^{1}H Spin-coupling constants in **3**, **5**, **6** and **8** S14
3. Table S2. ^{13}C-^{13}C Spin-coupling constants in **3**, **5**, **6** and **8** S15
4. Table S3. ^{1}H and ^{13}C Chemical shifts in **3**, **5**, **6** and **8** S16
5. Table S4. ^{1}H and ^{13}C Chemical shifts in **9** and **10** S17
6. Figures S1–S15. Representative ^{1}H and ^{13}C{^{1}H} NMR spectra of synthetic intermediates and products S18
7. Figure S16. Plots of DFT-calculated trans-*O*-glycosidic *J*-couplings in **3**^{c} as a function of either ϕ or ψ S33
8. Figure S17. Plots of DFT-calculated trans-*O*-glycosidic *J*-couplings in **4**^{c} as a function of either ϕ or ψ S34
9. Parameterized equations for ϕ- and ψ-dependent *J*-couplings in **3**^{c} and **4**^{c} S35
10. Figure S18. Parameter space plots for single-state *MA'AT* models of ϕ and ψ in the βGlcNAc-(1→4)-βMan linkages of **3**, **5**–**6** and **8**–**10** S36
11. Figure S19. Parameter space plots for single-state *MA'AT* models of ϕ and ψ in the αMan-(1→3)-βMan linkages of **3** and **5**–**7** S37
12. Figure S20. Population distributions of ϕ and ψ in the βGlcNAc-(1→4)-βMan linkages of **3**, **5**–**6** and **8**–**10** obtained from aqueous 1-µs MD simulations S38
13. Figure S21. Population distributions of ϕ and ψ in the αMan-(1→3)-βMan linkages of **3** and **5**–**7** obtained from aqueous 1-µs MD simulations S39
14. Table S5. Back-calculated *J*-couplings sensitive to ϕ and ψ in the βGlcNAc-(1→4)-βMan Linkages of **3**, **5**–**6** and **8**–**10** obtained from 1-µs aqueous MD simulations S40

15. Table S6. Back-calculated J-couplings sensitive to ϕ and ψ in the αMan-(1→3)-βMan Linkages of **4** and **6**–**8** obtained from 1-μs aqueous MD simulations S40
16. Scheme S5. Conventional ϕ- and ψ-dependent trans-*O*-glycosidiic J-couplings in the seven unique *O*-glycosiide linkages in **1** S41
17. Figures S22–S26. NMR spectra of $\mathbf{6}^{1''}$ and $\mathbf{6}^{2''}$ showing measurements of J_{CH} and J_{CC} values S42
18. Table S7. Experimental and CASPER-calculated ^{1}H chemical shfts in **3**, **5**–**6** and **8**–**10** S47
19. Table S8. Experimental and CASPER-calculated ^{13}C chemical shfts in **3**, **5**–**6** and **8**–**10** S48
20. Figure S27. Comparisons of experimental and CASPER-calculated ^{1}H chemical shifts in the βMan and βGlcNAc residues of **3** S49
21. Figure S28. Comparisons of experimental and CASPER-calculated H5 and H6b chemical shifts in the βMan residues of **3**, **5** and **9** S50
22. Figure S29. Comparisons of experimental and CASPER-calculated H3 and H4 chemical shifts in the βMan residues of **3**, **6**, **8** and **10** S51
23. Figure S30. Comparisons of experimental and CASPER-calculated ^{13}C chemical shifts in the βMan and βGlcNAc residues of **3** S52
24. Figure S31. Comparisons of experimental and CASPER-calculated C6 chemical shifts in the βMan residues of **3**, **5**, **8** and **9** S53
25. Figure S32. Comparisons of experimental and CASPER-calculated C3 chemical shifts in the βMan residues of **3**, **6**, **8** and **10** S54
26. Figure S33. Comparisons of experimental and CASPER-calculated C4 chemical shifts in the βMan residues of **3**, **6**, **8** and **10** S55
27. Discussion of data in Tables S7 and S8, and Figures S27–S33 S56
28. Cartesian coordinates for a DFT-optimized conformer of $\mathbf{3}^{c}$ S57
29. Full literature references 61 and 76 S58
30. Literature references S59

Preparation of Disaccharide 3 and Oligosaccharides 5, 6, 8, 9 and 10

Synopsis of Synthetic Routes

Starting from methyl β-D-mannopyranoside (**A**) (Scheme S1), the 3-OH and 6-OH were selectively benzylated via a stannylene acetal intermediate to give **B** in 66% yield (1–3). The 2-OH of **B** was selectively benzoylated with benzoyl chloride as the limiting reagent to give **C** in 87% yield. Peracetylated *N*-phthalimido glucosamine trichloroacetimidate (**G**) was prepared (Scheme S1) (4, 5) and coupled to acceptor **C** to give disaccharide **H** containing a β-(1 → 4) linkage (Scheme S2) whose formation was promoted by neighboring group participation of the *N*-phthalimido group in **G** (6, 7). Selective debenzylation of **H** gave **I** containing two unprotected hydroxyl groups in the reducing-end residue. Compound **I** was treated with hydrazine hydrate followed by acetylation of the amino and hydroxyl groups. Subsequent selective de-*O*-acetylation gave the **3** in 78% yield.

Trisaccharide **5** and tetrasaccharide **8** were prepared by a one-pot synthesis (Scheme S2). Disaccharide acceptor **I** and 1.6 equivalents of trichloroacetimidate **J** were coupled to give protected trisaccharide **K** in 34% yield and protected tetrasaccharide **L** in 39% yield. No protected trisaccharide **6** was detected in the reaction mixture. The α-Man-(1→6) linkage likely forms first to give **K** and a portion of **K** is subsequently converted to tetrasaccharide **L.** Steric hindrance from sugar residues at O4 and O6 in the reducing-end βMan residue of **I** did not prevent formation of a third *O*-glycosidic linkage at O3. Deprotection of **K** and **L** gave the **5** and **8**, respectively, in high yield.

To prepare trisaccharide **6** (Scheme S3), the 6-OH of disaccharide **I** was initially selectively benzoylated to leave only the 3-OH available for glycosylation. However, the coupling reaction with donor **J** failed to give the protected derivative of **6**. The apparent reduced reactivity of 3-OH is likely caused by potential inter-residue hydrogen bonding between the 3-OH hydrogen of the βMan residue and the ring oxygen of the GlcNAc residue (8–10). Previous studies have indicated that ether solvent stabilizes the oxocarbenium ion intermediate generated from glycosyl donor, resulting in acceptable yields in related coupling reactions (11, 12). However, an attempt to prepare protected **6** in this manner failed.

An alternate route to prepare **6** was pursued (Scheme S3). Disaccharide **N** was obtained by coupling acceptor **M** with donor **J** (13). After converting **N** to **O**, regioselective reductive cleavage of the 4,6-*O*-benzylidene acetal gave acceptor **P** containing a free 4-OH (**14**). The coupling of **P** with donor **G** gave trisaccharide **Q** in 76% yield. After debenzylation, trisaccharide **6** was obtained.

To prepare tetrasaccharide **9** and hexasaccharide **10** (Scheme S4), disaccharide donor **R** was prepared through self-condensation (1, 15, 16), and a one-pot reaction was conducted to prepare **S** and **T**. Significant degradation of donor **R** was observed during the coupling reaction, Consequently, a 4:1 ratio of donor **R** to acceptor **I** was employed to obtain sufficient hexasaccharide **T**. Although neighboring group participation was not possible in this coupling reaction, no detectable oligosaccharides containing β-Man-(1→3) and/or β-

Man-(1→6) linkages formed with acceptor **I**. Deprotection of **S** and **T** gave **9** and **10,** respectively.

A. Preparation of Glycosyl Acceptor **C** and Glycosyl Donor **G** (Scheme S1)

*A1. Methyl 2-O-Benzoyl-3,6-di-O-benzyl-β-D-mannopyranoside (**C**).* Methyl β-D-mannopyranoside (**A**) (5.90 g, 30.4 mmol) and dibutyltin oxide (17.0 g, 68.3 mmol) were added to anhydrous toluene (60 mL). After stirring at 100 °C for 3 h, the reaction mixture was concentrated to 30 mL, and benzyl bromide (20 mL, 168 mmol) and

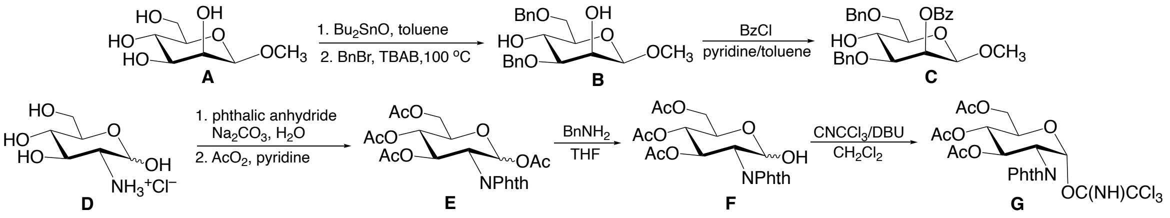

Scheme S1. Preparation of glycosyl acceptor **C** and glycosyl donor **G**.

tetrabutylammonium bromide (5.00 g, 15.5 mmol) were added. The resulting mixture was stirred at 100 °C for an additional 20 h, and then concentrated *in vacuo*. The residue was dissolved in ethyl acetate, washed with distilled water, dried over anhydrous Na_2SO_4, and concentrated to a syrup, which was purified by flash chromatography on silica gel to afford glycoside **B** (7.50 g, 20.1 mmol, 66%) (2). In this and the following steps, flash column chromatography on silica gel (preparative scale) was performed on a Reveleris® X2 flash chromatography system using a mixture of hexanes and ethyl acetate as the eluent. Compound **B** (7.50 g, 20.1 mmol) was dissolved in anhydrous toluene (60 mL) and anhydrous pyridine (8 mL) was added. Benzoyl chloride (2.50 mL, 21.5 mmol) was then added dropwise at 0 °C and the reaction mixture was stirred at 0 °C for 2 h. The mixture was evaporated to dryness and purified by flash chromatography on silica gel, affording product **C** (8.40 g, 17.6 mmol, 87%). **C**: ^{1}H NMR (600 MHz, $CDCl_3$): δ 8.12 (m, 2H), 7.57–7.26 (m, 13H), 5.85 (dd, J = 3.1, 1.0 Hz, H-2, 1H), 4.86 (d, J = 11.3 Hz, PhCH_2, 1H), 4.74 (d, J = 12.0 Hz, PhCH_2, 1H), 4.66 (d, J = 12.0 Hz, PhCH_2, 1H), 4.52 (d, J = 1.0 Hz, H-1, 1H), 4.51 (d, J = 11.3 Hz, PhCH_2, 1H), 4.07 (dd, J = 9.6, 9.3 Hz, H-4, 1H), 3.92 (m, H-6a, H-6b, 2H), 3.57 (dd, J = 9.3, 3.1 Hz, H-3, 1H), 3.55 (m, H-5, 1H), 3.53 (s, OCH_3, 3H), 2.96 (s, OH-4, 1H). ^{13}C NMR (150 MHz, $CDCl_3$): δ 166.1 (Ph*C*O), 138.3, 137.4, 133.1, 130.1, 130.0, 128.6, 128.5, 128.4, 128.2, 128.0, 127.7, 100.3 (C-1), 79.7 (C-3), 75.4 (C-5), 73.8 (Ph*C*H_2), 71.2 (Ph*C*H_2), 70.1 (C-6), 67.9 (C-2), 67.7 (C-4), 57.3 (O*C*H_3). HRMS (ESI-TOF) *m/z* $[M + Na]^+$: calcd for $C_{28}H_{30}O_7Na$, 501.1889; found, 557.1867.

*A2. 2-Deoxy-2-phthalimido-3,4,6-tri-O-acetyl-α-D-glucopyranosyl trichloroacetimidate (**G**).* D-Glucosamine hydrochloride (**D**) (6.33 g, 29.2 mmol), Na_2CO_3 (3.10 g, 29.2 mmol) and phthalic anhydride (4.32 g, 29.2 mmol) were added to distilled

water (38 mL). The mixture was stirred at room temperature (rt) overnight and concentrated to dryness. The residue was dissolved in pyridine (100 mL), and Ac_2O (40 mL, 423 mmol) was added. After stirring at rt for 12 h, the mixture was concentrated *in vacuo*. The residue was dissolved in ethyl acetate, washed with distilled water, dried over anhydrous Na_2SO_4, and concentrated to give compound **E**. Compound **E** was dissolved in THF (100 mL) and

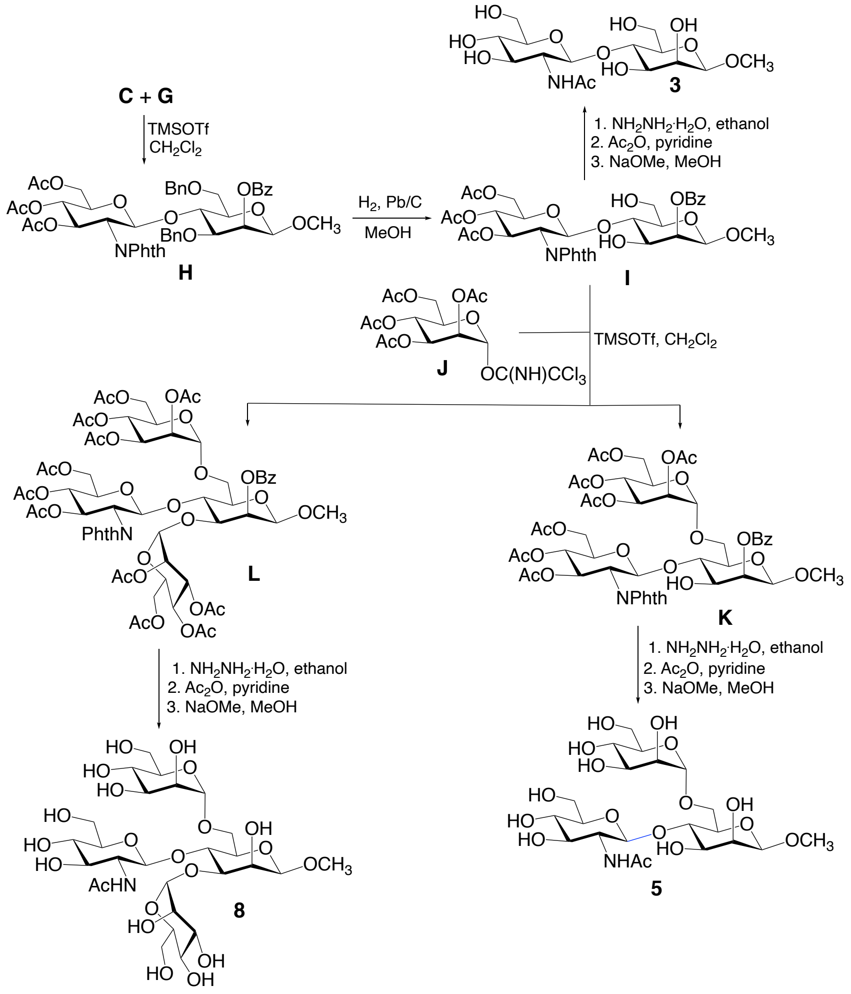

Scheme S2. Preparation of disaccharide **3**, trisaccharide **5** and tetrasaccharide **8** from **C**, **G** and **J**.

benzylamine (3.82

mL, 35.0 mmol) was added at 0 ^{o}C. After stirring for 4 h at rt, the THF was removed *in vacuo*. The residue was dissolved in ethyl acetate, washed with 1 *N* aqueous HCl solution, saturated aqueous $NaHCO_3$ solution, and distilled water sequentially, and then dried over anhydrous Na_2SO_4. After concentration, crystallization from an ethyl acetate/hexane (3:1) mixed solvent afforded pure compound **F** (8.00 g, 18.4 mmol, 63%). Compound **F** (3.00 g, 6.89 mmol) was dissolved in CH_2Cl_2 (30 mL), and trichloroacetonitrile (2.00 mL, 19.9 mmol)

and several drops of 1,8-diazabicyclo[5.4.0]undec-7-ene (DBU) were added. The reaction solution was stirred for 3 h at rt and concentrated *in vacuo*. Flash chromatography on silica gel gave trichloroacetimidate **G** (3.10 g, 5.35 mmol, 78%) (4, 5).

B. Preparation of Disaccharide **3**, Trisaccharide **5** and Tetrasaccharide **8** From **C**, **G** and **J** (Scheme S2)

*B1. Methyl 2-Deoxy-2-phthalimido-3,4,6-tri-O-acetyl-β-D-glucopyranosyl-(1→4)-2-O-benzoyl-3,6-di-O-benzyl-β-D-mannopyranoside (**H**).* Trichloroacetimidate **G** (750 mg, 1.30 mmol) and methyl glycoside **C** (480 mg, 1.00 mmol) were dissolved in anhydrous CH_2Cl_2 (20 mL) after drying under high vacuum, and the solution was treated with molecular sieves (4 Å) (2.0 g). A catalytic amount of TMSOTf (20 μL, 0.11 mmol) was added under a N_2 atmosphere at 0 °C. After 2 h, the reaction was quenched with the addition of a few drops of triethylamine and the molecular sieves were removed by filtration. The solution was concentrated to a syrup *in vacuo*, and the residue was purified by flash chromatography on silica gel to afford disaccharide **H** (790 mg, 0.88 mmol, 88%). **H**: ^{1}H NMR (600 MHz, $CDCl_3$): δ 8.03–7.10 (m, 19H), 5.77 (dd, *J* = 3.2, 0.9 Hz, H-2, 1H), 5.70 (d, *J* = 8.5 Hz, H-1′, 1H), 5.68 (dd, *J* = 10.6, 9.1 Hz, H-3′, 1H), 5.11 (dd, *J* = 10.6, 9.5 Hz, H-4′, 1H), 4.91 (d, *J* = 11.9 Hz, PhC*H*$_2$, 1H), 4.60 (d, *J* = 11.9 Hz, PhC*H*$_2$, 1H), 4.40 (d, *J* = 0.9 Hz, H-1, 1H), 4.36 (d, *J* = 12.0 Hz, PhC*H*$_2$, 1H), 4.30–4.26 (m, H-4, H-2′, 2H) 4.23 (d, *J* = 12.0 Hz, PhC*H*$_2$, 1H), 3.95 (dd, *J* = 12.3, 3.8 Hz, H-6a′, 1H), 3.78 (dd, *J* = 9.0, 3.2 Hz, H-3, 1H), 3.74 (dd, *J* = 12.3, 2.0 Hz, H-6b′, 1H), 3.55 (dd, *J* = 11.5, 4.0 Hz, H-6a, 1H), 3.51 (dd, *J* = 11.5, 1.7 Hz, H-6b, 1H), 3.42 (m, H-5, 1H), 3.40 (s, OCH_3, 3H), 3.35 (m, H-5′, 1H). 1.94 (s, COC*H*$_3$, 3H), 1.92 (s, COC*H*$_3$, 3H), 1.80 (s, COC*H*$_3$, 3H). ^{13}C NMR (150 MHz, $CDCl_3$): δ 170.6 (*C*OCH$_3$), 170.1 (*C*OCH$_3$), 169.4 (*C*OCH$_3$), 165.9 (Ph*C*O), 138.4–126.6, 100.1 (C-1), 98.2 (C-1′), 78.6 (C-3), 75.1 (C-5), 74.2 (C-4), 72.9 (Ph*C*H$_2$), 71.6 (C-5′), 70.8 (C-3′), 70.7 (Ph*C*H$_2$), 68.5 (C-4′), 68.2 (C-2), 68.0 (C-6), 61.3 (C-6′), 57.0 (O*C*H$_3$), 55.3 (C-2′), 20.7 (CO*C*H$_3$), 20.6 (CO*C*H$_3$), 20.4 (CO*C*H$_3$). HRMS (ESI-TOF) *m/z* [M + Na]$^+$: calcd for $C_{48}H_{49}NO_{16}Na$, 918.2944; found, 918.2818.

*B2. Methyl 2-Deoxy-2-phthalimido-3,4,6-tri-O-acetyl-β-D-glucopyranosyl-(1→4)-2-O-benzoyl-β-D-mannopyranoside (**I**).* Compound **H** (750 mg, 0.84 mmol) was dissolved in methanol (20 mL) and treated with Pd/C (10%, 200 mg) and H_2 overnight. The Pd/C catalyst was removed by filtration and the filtrate was concentrated to dryness *in vacuo* to afford **I** (580 mg, 0.81 mmol, 96%). **I**: ^{1}H NMR (600 MHz, $CDCl_3$): δ 8.03–7.39 (m, 9H), 5.79 (dd, *J* = 10.6, 9.0 Hz, H-3′, 1H), 5.68 (dd, *J* = 3.3, 0.9 Hz, H-2, 1H), 5.51 (d, *J* = 8.5 Hz, H-1′, 1H), 5.05 (dd, *J* = 10.2, 9.2 Hz, H-4′, 1H), 4.48 (d, *J* = 0.9 Hz, H-1, 1H), 4.34 (dd, *J* = 10.6, 8.5 Hz, H-2′, 1H), 4.15 (dd, *J* = 12.2, 2.3 Hz, H-6a′, 1H), 4.08 (dd, *J* = 12.2, 7.2 Hz, H-6b′, 1H), 4.00 (ddd, *J* = 10.2, 7.2, 2.2 Hz, H-5′, 1H), 3.96 (dd, *J* = 9.3, 9.1 Hz, H-4, 1H), 3.85 (dd, *J* =

9.1, 3.4 Hz, H-3, 1H), 3.45 (dd, *J* = 12.1, 2.1 Hz, H-6a, 1H), 3.38 (s, OCH_3, 3H), 3.25 (ddd, *J* = 9.3, 3.7, 2.1 Hz, H-5, 1H), 3.20 (dd, *J* = 12.1, 3.7 Hz, H-6b, 1H), 2.00 (s, $COCH_3$, 3H), 1.83 (s, $COCH_3$, 3H), 1.77 (s, $COCH_3$, 3H). ^{13}C NMR (150 MHz, $CDCl_3$): δ 170.7 ($COCH_3$), 170.1 ($COCH_3$), 169.6 ($COCH_3$), 165.8 (Ph*C*O), 133.1, 130.1, 130.0, 128.4, 100.6 (C-1), 99.0 (C-1′), 79.3 (C-4), 74.5 (C-5), 72.0 (C-5′), 71.2 (C-3), 70.6 (C-2), 70.4 (C-3′), 69.0 (C-4′), 62.1 (C-6′), 60.9 (C-6), 57.4 (OCH_3), 54.7 (C-2′), 20.7 ($COCH_3$), 20.5 ($COCH_3$), 20.2 ($COCH_3$). HRMS (ESI-TOF) *m/z* $[M + Na]^+$: calcd for $C_{34}H_{37}NO_{16}Na$, 738.2010; found, 738.1913.

*B3. Methyl 2-Acetamido-2-deoxy-β-D-glucopyranosyl-(1→4)-β-D-mannopyranoside (**3**).* Compound **I** (100 mg, 0.140 mmol) dissolved in ethanol (10 mL) and hydrazine hydrate (1.50 mL) was added. After refluxing for 20 h, the reaction mixture was concentrated *in vacuo* to a syrup, which was dried under high vacuum. The dried residue was dissolved in pyridine (10 mL) and Ac_2O (2.00 mL) was added. The mixture was stirred at rt overnight and concentrated *in vacuo*. The residue was purified by flash chromatography on silica gel to give an acetylated disaccharide, which was treated with sodium methoxide in methanol (20 mL, pH > 10) overnight. Final product (**3**) was dissolved in ~0.5 mL of distilled water, and the solution was applied to a column (2.5 x 100 cm) containing Bio-gel P2 gel-filtration resin (45–90 μm). The column was eluted with distilled water at ~1.5 mL/min, and fractions (~10 mL) were collected. Fractions containing pure product were collected and concentrated at 30 °C *in vacuo* to give **3** as a white solid (43 mg, 0.109 mmol, 78%). Characterization of **2** by ^1H and ^{13}C NMR is found in the Supporting Information. HRMS (ESI-TOF) *m/z* $[M + Na]^+$: calcd for $C_{15}H_{26}NO_{11}Na$, 419.1405; found, 419.1391.

*B4. Methyl 2-Deoxy-2-phthalimido-3,4,6-tri-O-acetyl-β-D-glucopyranosyl-(1→4)-(2,3,4,6-tetra-O-acetyl-α-D-mannopyranosyl-(1→6))-2-O-benzoyl-β-D-mannopyranoside (**K**) and Methyl 2-Deoxy-2-phthalimido-3,4,6-tri-O-acetyl-β-D-glucopyranosyl-(1→4)-(2,3,4,6-tetra-O-acetyl-α-D-mannopyranosyl-(1→3;1→6))-2-O-benzoyl-β-D-mannopyranoside (**L**).* Compound **I** (900 mg, 1.26 mmol) and 2,3,4,6-tetra-*O*-acetyl-α-D-mannopyranosyl trichloroacetimidate **J** (990 mg, 2.01 mmol) were dissolved in anhydrous CH_2Cl_2 (20 mL) after drying under high vacuum, and the solution was treated with molecular sieves (4 Å) (1.0 g). A catalytic amount of TMSOTf (30 μL, 165 μmol) was added under a N_2 atmosphere at 0 °C. After 2 h, the reaction was quenched with the addition of a few drops of triethylamine and the molecular sieves were removed by filtration. The solution was concentrated *in vacuo*, and the residue was purified by flash chromatography on silica gel to afford trisaccharide **K** (450 mg, 0.43 mmol, 34%) and tetrasaccharide **L** (670 mg, 0.49 mmol, 39%). **K**: ^1H NMR (600 MHz, $CDCl_3$): δ 8.01–7.43 (m, 9H), 5.77 (dd, *J* = 10.6, 9.0 Hz, H-3′, 1H), 5.65 (dd, *J* = 3.6, 0.9 Hz, H-2, 1H), 5.44 (d, *J* = 8.5 Hz, H-1′, 1H), 5.27 (dd, *J* = 10.1, 3.3 Hz, H-3″, 1H), 5.19 (dd, *J* = 10.1, 10.0 Hz, H-4″, 1H), 5.10 (dd, *J* = 3.3, 1.7 Hz,

H-2″, 1H), 5.05 (dd, *J* = 10.2, 9.0 Hz, H-4′, 1H), 4.43 (d, *J* = 0.9 Hz, H-1, 1H), 4.33 (dd, *J* = 10.6, 8.5 Hz, H-2′, 1H), 4.29 (d, *J* = 1.7 Hz, H-1″, 1H), 4.19 (dd, *J* = 12.1, 5.0 Hz, H-6a″, 1H), 4.14–4.09 (m, H-6a′, H-6b′, 2H), 4.05–4.00 (m, H-6b″, H-5′, 2H), 3.83–3.80 (m, H-5″, H-3, 2H), 3.70 (dd, *J* = 8.9, 8.9 Hz, H-4, 1H), 3.40–3.32 (m, H-5, H-6a, H-6b, 3H), 3.36 (s, OCH_3, 3H), 2.11 (s, $COCH_3$, 3H), 2.02 (s, $COCH_3$, 3H), 2.01 (s, $COCH_3$, 3H), 1.98 (s, $COCH_3$, 3H), 1.96 (s, $COCH_3$, 3H), 1.81 (s, $COCH_3$, 3H), 1.76 (s, $COCH_3$, 3H). ^{13}C NMR (150 MHz, $CDCl_3$): δ 170.6 ($COCH_3$), 170.5 ($COCH_3$), 170.0 ($COCH_3$), 169.8 ($COCH_3$), 169.7 ($COCH_3$), 169.5 ($COCH_3$), 169.4 ($COCH_3$), 165.8 (Ph*C*O), 134.7, 133.0, 130.4, 129.9, 128.4, 100.3 (C-1), 98.5 (C-1′), 97.1 (C-1″), 79.6 (C-4), 73.8 (C-5), 72.0 (C-5′), 71.0 (C-3), 70.4 (C-2), 70.3 (C-3′), 69.1 (C-3″), 68.9 (C-2″), 68.8 (C-4′), 68.6 (C-5″), 65.9 (C-4″), 65.00 (C-6), 62.2 (C-6″), 61.8 (C-6′), 57.1 (OCH_3), 54.6 (C-2′), 20.9 ($COCH_3$), 20.8 ($COCH_3$), 20.7 ($COCH_3$), 20.6 ($COCH_3$), 20.5 ($COCH_3$), 20.4 ($COCH_3$), 20.1 ($COCH_3$). HRMS (ESI-TOF) *m/z* $[M + Na]^+$: calcd for $C_{48}H_{55}NO_{25}Na$, 1068.2961; found, 1068.2836.

Compound **L**: ^{1}H NMR (600 MHz, $CDCl_3$): δ 8.03–7.42 (m, 9H), 5.74 (dd, *J* = 10.7, 9.0 Hz, H-3″, 1H), 5.65 (dd, *J* = 3.3, 0.8 Hz, H-2, 1H), 5.36–5.32 (m, H-2′, H-1″, H-4‴, 3H), 5.30–5.24 (m, H-4′, H-2‴, H-3‴, 3H), 5.18 (dd, *J* = 10.1, 9.0 Hz, H-4″, 1H), 5.14 (dd, *J* = 10.0, 3.4 Hz, H-3′, 1H), 5.12 (d, *J* = 1.8 Hz, H-1′, 1H), 4.91 (d, *J* = 1.7 Hz, H-1‴, 1H), 4.40 (d, *J* = 0.9 Hz, H-1, 1H), 4.35 (dd, *J* = 12.3, 4.1 Hz, H-6a‴, 1H), 4.32 (ddd, *J* = 10.0, 4.4, 2.2 Hz, H-5′, 1H), 4.28–4.22 (m, H-6a′, H-2″, H-6a″, H-6b‴, 4H), 4.20–4.13 (m, H-4, H-6b′, H-5‴, 3H), 4.07 (dd, *J* = 12.4, 2.5 Hz, H-6b″, 1H), 3.95 (ddd, *J* = 10.0, 7.3, 2.5 Hz, H-5″, 1H), 3.83 (dd, *J* = 9.4, 3.4 Hz, H-3, 1H), 3.70–3.64 (m, H-6a, H-6b, 2H), 3.36 (s, OCH_3, 3H), 3.25 (m, H-5, 1H), 2.21 (s, $COCH_3$, 3H), 2.17 (s, $COCH_3$, 3H), 2.08 (s, $COCH_3$, 3H), 2.07 (s, $COCH_3$, 3H), 2.04 (s, $COCH_3$, 3H), 2.02 (s, $COCH_3$, 3H), 1.96 (s, $COCH_3$, 3H), 1.95 (s, $COCH_3$, 3H), 1.87 (s, $COCH_3$, 3H), 1.80 (s, $COCH_3$, 3H), 1.54 (s, $COCH_3$, 3H). ^{13}C NMR (150 MHz, $CDCl_3$): δ 170.9 ($COCH_3$), 170.8 ($COCH_3$), 170.7 ($COCH_3$), 170.3 ($COCH_3$), 170.1 ($COCH_3$), 169.9 ($COCH_3$), 169.9 ($COCH_3$), 169.7 ($COCH_3$), 169.6 ($COCH_3$), 169.5 ($COCH_3$), 169.2 ($COCH_3$), 166.1 (Ph*C*O), 133.2, 130.0, 129.8, 128.6, 100.0 (C-1), 99.4 (C-1′), 97.8 (C-1‴), 97.4 (C-1″), 75.8 (C-3), 74.6 (C-5), 73.0 (C-4), 72.4 (C-5″), 70.9 (C-2), 70.6 (C-3″), 69.5 (C-4″), 69.5 (C-2′), 69.3 (C-3‴), 69.2 (C-5′), 69.0 (C-2‴), 68.9 (C-5‴), 68.4 (C-3′), 66.4 (C-4′), 65.9 (C-4‴), 65.4 (C-6), 62.6 (C-6′), 62.4 (C-6‴), 62.2 (C-6″), 57.22 (OCH_3), 54.5 (C-2″), 21.1 ($COCH_3$), 21.0 ($COCH_3$), 20.9 ($COCH_3$), 20.9 ($COCH_3$), 20.8 ($COCH_3$), 20.8 ($COCH_3$), 20.7 ($COCH_3$), 20.7 ($COCH_3$), 20.7 ($COCH_3$), 20.6 ($COCH_3$), 20.0 ($COCH_3$). HRMS (ESI-TOF) *m/z* $[M + Na]^+$: calcd for $C_{62}H_{73}NO_{34}Na$, 1398.3912; found, 1398.3785.

*B5. Methyl 2-Acetamido-2-deoxy-β-D-glucopyranosyl-(1→4)-(α-D-mannopyranosyl-(1→6))-β-D-mannopyranoside (**5**).* Using the same protocol as described for the preparation of **3**, compound **K** (220 mg, 0.210 mmol) was converted to trisaccharide **5** as a syrup (80 mg, 0.143 mmol, 68%). Characterization of **5** by ^{1}H and ^{13}C NMR is found in the Supporting

Information. HRMS (ESI-TOF) *m/z* [M + Na]+: calcd for $C_{21}H_{37}NO_{16}Na$, 582.2010; found, 582.1949.

*B6. Methyl 2-Acetamido-2-deoxy-β-D-glucopyranosyl-(1→4)(α-D-mannopyranosyl-(1→3; 1→6))-β-D-mannopyranoside (**8**).* Using the same protocol as described for the preparation of **3**, compound **L** (200 mg, 0.163 mmol) was converted to tetrasaccharide **8** as a syrup (84 mg, 0.116 mmol, 71%). Characterization of **8** by ^{1}H and ^{13}C NMR is found in the Supporting Information. HRMS (ESI-TOF) *m/z* [M + Na]+: calcd for $C_{27}H_{47}NO_{21}Na$, 744.2538; found, 744.2505.

C. Preparation of Trisaccharide **6** from Trichloroacetimidates **G** and **J**, and a 4,6-*O*-Benzylidene βMan Acceptor **M** (Scheme S3).

*C1. 2,3,4,6-Tetra-O-acetyl-α-D-mannopyranosyl-(1→3)-2-O-acetyl-4,6-O-benzylidine-β-D-mannopyranoside (**O**).* Disaccharide **N** was prepared from **M** as described previously (13). Compound **N** (306 mg, 0.500 mmol) was dissolved in pyridine (10 mL) and acetic anhydride (0.20 mL, 2.13 mmol) was added. The reaction solution was stirred for 3 h at rt and concentrated *in vacuo*. Flash chromatography on silica gel gave compound **O** (310 mg, 0.475 mmol, 95%). **O**: ^{1}H NMR (600 MHz, $CDCl_3$): δ 7.41–7.30 (m, 5H), 5.60 (s, PhC*H*, 1H), 5.53 (dd, *J* = 3.2, 1.2 Hz, H-2, 1H), 5.35 (dd, *J* = 3.3, 1.8 Hz, H-2′, 1H), 5.11

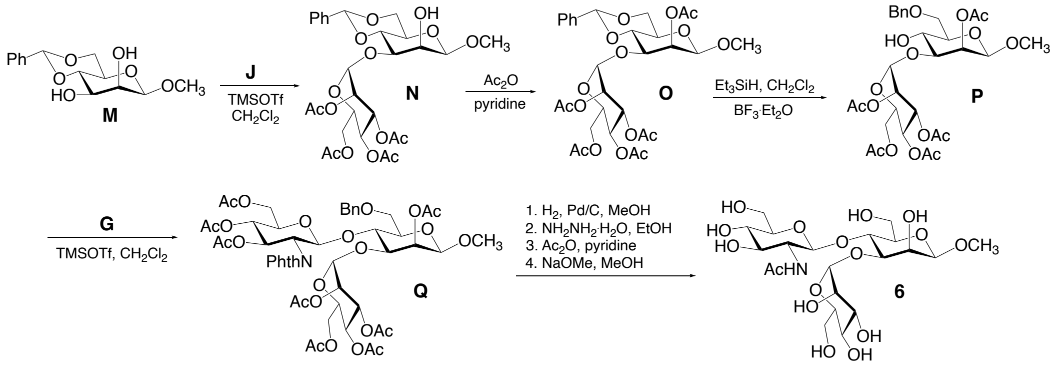

Scheme S3. Preparation of trisaccharide **6** from trichloroacetimidates **G** and **J**, and a 4,6-*O*-benzyidene βMan acceptor **M**.

(dd, *J* = 10.1, 10.1 Hz, H-4′, 1H), 5.20 (d, *J* = 1.8 Hz, H-1′, 1H), 5.16 (dd, *J* = 10.2, 3.2 Hz, H-3′, 1H), 4.57 (d, *J* = 1.2 Hz, H-1, 1H), 4.37 (dd, *J* = 10.5, 5.0 Hz, H-6a, 1H), 4.29 (m, H-6a′, 1H), 4.14–4.10 (m, H-5′, H-6a′, 2H), 4.06–4.01 (m, H-3, H-4, 2H), 3.90 (dd, *J* = 10.5, 10.3 Hz, H-6b, 1H), 3.54 (s, OCH_3, 3H), 3.41 (m, H-5, 1H), 2.26 (s, COC*H*$_3$, 3H), 2.10 (s, COC*H*$_3$, 3H), 2.07 (s, COC*H*$_3$, 3H), 2.06 (s, COC*H*$_3$, 3H), 1.96 (s, COC*H*$_3$, 3H). ^{13}C NMR (150 MHz, $CDCl_3$): δ 170.9 (*C*OCH$_3$), 170.8 (*C*OCH$_3$), 170.0 (*C*OCH$_3$), 170.0 (*C*OCH$_3$), 169.8 (*C*OCH$_3$), 137.0, 129.1, 128.3, 126.0, 101.5 (Ph*C*H), 100.7 (C-1), 98.4 (C-1′), 78.8 (C-4), 73.1 (C-3), 70.2 (C-2), 69.4 (C-5′), 69.2 (C-2′), 69.0 (C-3′), 68.4 (C-6), 66.9 (C-5),

65.6 (C-4′), 62.4 (C-6′), 57.8 (O*C*H_3), 21.0 (CO*C*H_3), 20.9 (CO*C*H_3), 20.9 (CO*C*H_3), 20.9 (CO*C*H_3), 20.8 (CO*C*H_3). HRMS (ESI-TOF) *m/z* [M + Na]$^+$: calcd for $C_{30}H_{38}O_{16}Na$, 677.2058; found, 677.2061.

*C2. 2,3,4,6-Tetra-O-acetyl-α-D-mannopyranosyl-(1→3)-2-O-acetyl-6-O-benzyl-β-D-mannopyranoside (**P**).* Compound **O** (250 mg, 0.38 mmol) was dissolved in anhydrous CH_2Cl_2 (20 mL), and triethylsilane (0.60 mL, 3.80 mmol) and $BF_3{\cdot}Et_2O$ (0.095 mL, 0.760 mmol) were added at 0 °C. The reaction mixture was stirred at rt for 3 h. The mixture was then diluted with CH_2Cl_2 (20 mL) and the resulting solution was washed with aqueous $NaHCO_3$ solution (1 *N*) (20 mL), followed by distilled water (20 mL). The organic phase was dried over Na_2SO_4 and concentrated, and the residue was purified on a silica gel column to afford **P** as white foam (170 mg, 0.26 mmol, 68%). **P**: ^{1}H NMR (600 MHz, $CDCl_3$): δ 7.40–7.29 (m, 5H), 5.41 (dd, *J* = 3.5, 0.9 Hz, H-2, 1H), 5.32 (dd, *J* = 3.3, 1.6 Hz, H-2′, 1H), 5.30 (dd, *J* = 10.1, 10.1 Hz, H-4′, 1H), 5.23 (d, *J* = 1.6 Hz, H-1′, 1H), 5.16 (dd, *J* = 10.2, 3.3 Hz, H-3′, 1H), 4.62 (d, *J* = 11.8 Hz, PhC*H*$_2$, 1H), 4.55 (d, *J* = 11.8 Hz, PhC*H*$_2$, 1H), 4.45 (d, *J* = 0.9 Hz, H-1, 1H), 4.27 (dd, *J* = 12.4, 4.7 Hz, H-6a′, 1H), 4.17–4.12 (m, H-5′, H-6a′, 2H), 3.94 (dd, *J* = 9.5, 9.5 Hz, H-4, 1H), 3.83–3.75 (m, H-6a, H-3, H-6b, 3H), 3.49 (s, OCH_3, 3H), 3.43 (m, H-5, 1H), 2.19 (s, COC*H*$_3$, 3H), 2.12 (s, COC*H*$_3$, 3H), 2.09 (s, COC*H*$_3$, 3H), 2.05 (s, COC*H*$_3$, 3H), 1.96 (s, COC*H*$_3$, 3H). ^{13}C NMR (150 MHz, $CDCl_3$): δ 170.9 (*C*OCH_3), 170.7 (*C*OCH_3), 170.2 (*C*OCH_3), 170.0 (*C*OCH_3), 170.0 (*C*OCH_3), 137.5, 128.6, 128.0, 127.9, 99.9 (C-1), 99.0 (C-1′), 77.3 (C-3), 74.0 (Ph*C*H_2), 73.9 (C-5), 71.0 (C-6), 70.7 (C-4), 70.1 (C-2), 69.4 (C-2′), 69.2 (C-5′), 69.1 (C-3′), 65.6 (C-4′), 62.4 (C-6′), 57.3 (O*C*H_3), 20.9 (CO*C*H_3), 20.9 (CO*C*H_3), 20.9 (CO*C*H_3), 20.9 (CO*C*H_3), 20.8 (CO*C*H_3), 20.7 (CO*C*H_3). HRMS (ESI-TOF) *m/z* [M + Na]$^+$: calcd for $C_{30}H_{40}O_{16}Na$, 679.2214; found, 679.2131.

*C3. Methyl 2,3,4,6-Tetra-O-acetyl-α-D-mannopyranosyl-(1→3)-(2-deoxy-2-phthalimido-3,4,6-tri-O-acetyl-β-D-glucopyranosyl-(1→4))-2-O-acetyl-6-O-benzyl-β-D-mannopyranoside (**Q**).* Acceptor **P** (160 mg, 244 µmol) and donor **G** (180 mg, 310 µmol) were dissolved in anhydrous CH_2Cl_2 (20 mL) after drying under high vacuum, and the solution was treated with molecular sieves (4 Å) (0.5 g). A catalytic amount of TMSOTf (10 µL, 55 µmol) was added under a N_2 atmosphere at 0 °C. After 2 h, the reaction was quenched with the addition of a few drops of triethylamine and the molecular sieves were removed by filtration. The solution was concentrated, and the residue was purified by flash chromatography on silica gel to afford trisaccharide **Q** (200 mg, 186 µmol, 76%). **Q**: ^{1}H NMR (600 MHz, $CDCl_3$): δ 7.85–7.26 (m, 9H), 5.72 (dd, *J* = 10.7, 8.9 Hz, H-3″, 1H), 5.46 (dd, *J* = 3.4, 1.8 Hz, H-2′, 1H), 5.37 (d, *J* = 8.4 Hz, H-1″, 1H), 5.36 (dd, *J* = 3.5, 1.0 Hz, H-2, 1H), 5.33 (dd, *J* = 10.1, 10.1 Hz, H-4′, 1H), 5.21 (dd, *J* = 10.3, 3.3 Hz, H-3′, 1H), 5.15 (dd, *J* = 10.1, 8.9 Hz, H-4″, 1H), 5.14 (d, *J* = 1.8 Hz, H-1′, 1H), 4.61 (s, PhC*H*$_2$, 2H), 4.36 (dd, *J* = 9.7, 9.7 Hz, H-4, 1H), 4.31 (d, *J* = 1.1 Hz, H-1, 1H), 4.26–4.23 (m, H-6a′, H-6a″,

2H), 4.19 (dd, J = 10.7, 8.4 Hz, H-2″, 1H), 4.17 (m, H-5′, 1H), 4.12 (dd, J = 12.2, 2.6 Hz, H-6b′, 1H), 4.09 (dd, J = 12.2, 2.5 Hz, H-6b″, 1H), 3.71 (dd, J = 9.3, 3.4 Hz, H-3, 1H), 3.62 (dd, J = 11.2, 1.4 Hz, H-6a, 1H), 3.54 (dd, J = 11.2, 4.0 Hz, H-6b, 1H), 3.51 (m, H-5″, 1H), 3.41 (s, OCH_3, 3H), 3.16 (ddd, J = 9.8, 4.0, 1.4 Hz, H-5, 1H), 2.25 (s, $COCH_3$, 3H), 2.18 (s, $COCH_3$, 3H), 2.10 (s, $COCH_3$, 3H), 2.04 (s, $COCH_3$, 3H), 2.00 (s, $COCH_3$, 3H), 2.00 (s, $COCH_3$, 3H), 1.96 (s, $COCH_3$, 3H), 1.81 (s, $COCH_3$, 3H). ^{13}C NMR (150 MHz, $CDCl_3$): δ 170.8, 170.8, 170.6, 170.4, 170.1, 169.8, 169.8, 169.6 (8 $COCH_3$), 138.4, 128.4, 127.7, 127.6, 99.9 (C-1), 99.3 (C-1′), 96.5 (C-1″), 75.6 (C-3), 74.9 (C-5), 73.0 ($PhCH_2$), 72.3 (C-5″), 71.8 (C-4), 70.7 (C-3″), 70.5 (C-2), 69.6 (C-4″), 69.51 (C-2′), 69.3 (C-5′), 68.7 (C-3′), 68.4 (C-6), 66.0 (C-4′), 62.6 (C-6″), 62.5 (C-6′), 57.3 (OCH_3), 54.6 (C-2″), 21.1, 20.9, 20.9, 20.9, 20.8, 20.8, 20.6, 20.6 (8 $COCH_3$). HRMS (ESI-TOF) *m/z* $[M + Na]^+$: calcd for $C_{50}H_{59}NO_{25}Na$, 1096.3274; found, 1096.3201.

*C4. Methyl α-D-Mannopyranosyl-(1→3)-(2-acetamido-2-deoxy-β-D-glucopyranosyl-(1→4))-β-D-mannopyranoside (**6**).* Using the same protocol as described for the preparation of compound **2**, compound **Q** (170 mg, 158 µmol) was converted to trisaccharide **6** as a syrup (65 mg, 116 µmol, 73%). Characterization of **6** by ^1H and ^{13}C NMR is found in the Supporting Information. HRMS (ESI-TOF) *m/z* $[M + Na]^+$: calcd for $C_{21}H_{37}NO_{16}Na$, 582.2010; found, 582.1997.

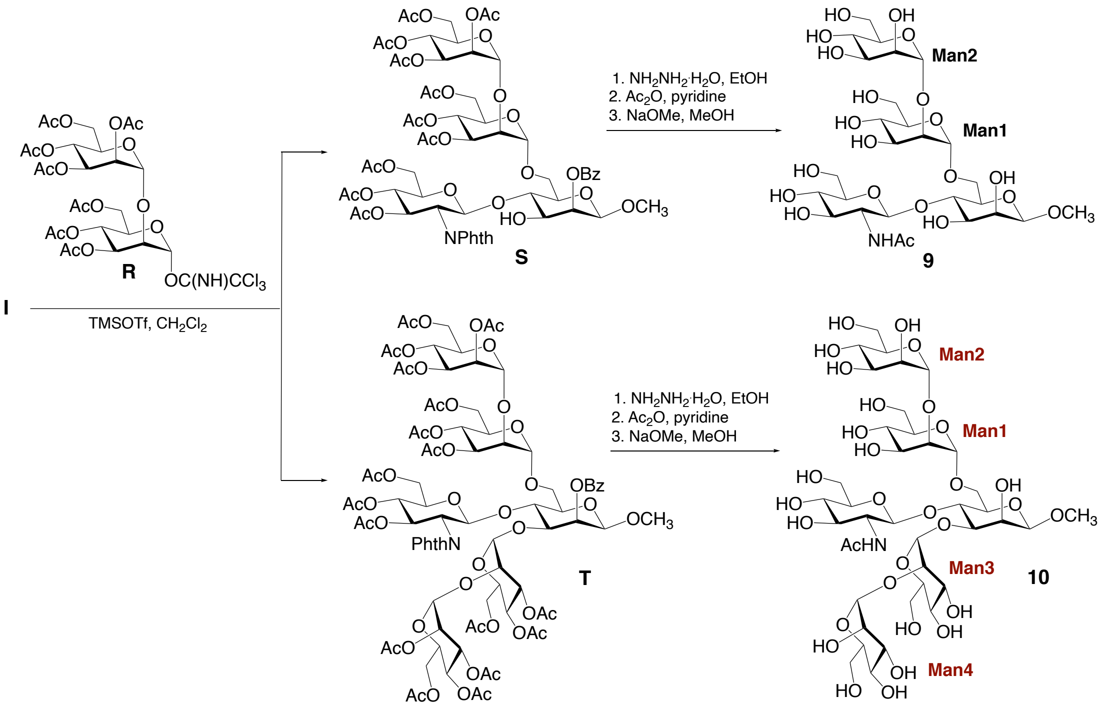

Scheme S4. Preparation of tetrasaccharide **9** and hexasaccharide **10** from disaccharide trichloroacetimidate donor **R** and disaccharide acceptor **I**.

D. Preparation of Tetrasaccharide **9** and Hexasaccharide **10** from Disaccharide Trichloroacetimidate Donor **R** and Disaccharide Acceptor **I** (Scheme S4)

*D1. Methyl 2-Deoxy-2-phthalimido-3,4,6-tri-O-acetyl-β-D-glucopyranosyl-(1→4)-(2,3,4,6-tetra-O-acetyl-α-D-mannopyranosyl-(1→2)-3,4,6-tetra-O-acetyl-α-D-mannopyranosyl-(1→6))-2-O-benzoyl-β-D-mannopyranoside (**S**) and Methyl 2-Deoxy-2-phthalimido-3,4,6-tri-O-acetyl-β-D-glucopyranosyl-(1→4)-(2,3,4,6-tetra-O-acetyl-α-D-mannopyranosyl-(1→2)-3,4,6-tetra-O-acetyl-α-D-mannopyranosyl-(1→3;1→6))-2-O-benzoyl-β-D-mannopyranoside (**T**).* Compound **I** (110 mg, 0.15 mmol) and 2,3,4,6-tetra-*O*-acetyl-α-D-mannopyranosyl-(1→2)-3,4,6-tetra-*O*-acetyl-α-D-mannopyranosyl trichloroacetimidate **R** (460 mg, 0.60 mmol) (1, 15). were dissolved in anhydrous CH_2Cl_2 (15 mL) after drying under high vacuum, and the solution was treated with molecular sieves (4 Å) (0.5 g). TMSOTf (30 μL, 165 μmol) was added under a N_2 atmosphere at 0 °C. After 4 h, the reaction was quenched with the addition of a few drops of triethylamine and the molecular sieves were removed by filtration. The solution was concentrated *in vacuo*, and the residue was purified by flash chromatography on silica gel to afford tetrasaccharide **S** (72 mg, 54 μmol, 36%) and hexasaccharide **T** (61 mg, 31 μmol, 21%). **S**: ^{1}H NMR (600 MHz, $CDCl_3$): δ 7.95–7.36 (m, 9H), 5.79 (dd, *J* = 10.6, 9.1 Hz, 1H), 5.65 (dd, *J* = 3.3, 0.9 Hz,1H), 5.53 (d, *J* = 8.5 Hz, 1H), 5.36-5.28 (m, 3H), 5.19 (dd, *J* = 10.1, 10.1, 1H), 5.10 (dd, *J* = 3.4, 1.7 Hz, 1H), 5.07 (dd, *J* = 10.0, 9.4 Hz, 1H), 4.85 (d, *J* = 1.8 Hz, 1H), 4.67 (d, *J* = 1.7 Hz, 1H), 4.44 (d, *J* = 0.9 Hz, 1H), 4.34 (dd, *J* = 10.6, 8.5 Hz, 1H), 4.19–3.97 (m, 9H), 3.88–3.82 (m, 2H), 3.72 (m, 1H), 3.44–3.32 (m, 3H), 3.37 (s, 3H), 2.10 (s, 3H), 2.09 (s, 3H), 2.08 (s, 3H), 2.04 (s, 3H), 1.99 (s, 3H), 1.98 (s, 3H), 1.95 (s, 3H), 1.90 (s, 3H), 1.82 (s, 3H), 1.79 (s, 3H). ^{13}C NMR (150 MHz, $CDCl_3$): δ 170.9, 170.7, 170.7, 170.5, 170.1, 169.7, 169.6, 169.6, 169.5, 169.4, 165.9, 133.1, 130.3, 129.9, 128.3, 124.0, 100.6, 99.0, 98.8, 98.7, 79.2, 77.1, 74.4, 72.0, 71.0, 70.8, 70.5, 70.4, 69.8, 69.2, 69.0, 68.9, 68.7, 66.1, 65.7, 65.5, 62.0, 61.9, 61.9, 60.4, 57.1, 54.7, 21.1, 21.0, 20.9, 20.9, 20.8, 20.7, 20.7, 20.6, 20.5, 20.3. HRMS (ESI-TOF) *m/z* $[M + Na]^+$: calcd for $C_{62}H_{73}NO_{34}Na$, 1356.3808; found, 1356.3817.

Compound **T**: ^{13}C NMR (150 MHz, methanol-d_4): δ 172.8, 172.4, 172.2, 172.0, 171.8, 171.1, 171.6, 171.5, 171.1, 171.0, 168.4, 167.9, 136.4, 134.3, 132.5, 132.3, 131.5, 131.0, 129.6, 122.8, 120.7, 101.2, 100.9, 100.7, 100.4, 100.1, 100.0, 79.4, 78.9, 75.8, 75.5, 75.4, 73.5, 73.1, 72.1, 71.7, 70.9, 70.6, 70.5, 70.2, 67.8, 67.3, 67.1, 67.0, 66.0, 64.3, 63.4, 63.2, 63.1, 62.9, 56.4, 21.5, 21.2, 21.1, 21.0, 20.9, 20.8, 20.7, 20.5. HRMS (ESI-TOF) *m/z* $[M + Na]^+$: calcd for $C_{86}H_{105}NO_{50}Na$, 1975.7238; found, 1975.7241.

D2. Methyl 2-Acetamido-2-deoxy-β-D-glucopyranosyl-(1→4)(α-D-mannopyranosyl-(1→2)-α-D-mannopyranosyl-(1→6))-β-D-mannopyranoside (**9**). Using the same protocol as described for the preparation of compound **3**, compound **S** (68 mg, 51 μmol) was converted

to tetrasaccharide **9** as a syrup (25 mg, 33 μmol, 65%). Characterization of **9** by ^{1}H and ^{13}C NMR is found in the Supporting Information. HRMS (ESI-TOF) *m/z* [M + Na]$^{+}$: calcd for $C_{27}H_{47}NO_{21}Na$, 744.2538; found, 744.2517.

D3. Methyl 2-Acetamido-2-deoxy-β-D-glucopyranosyl-(1→4)(α-D-mannopyranosyl-(1→2)-α-D-mannopyranosyl-(1→3;1→6))-β-D-mannopyranoside (**10**). Using the same protocol as described for the preparation of compound **3**, compound **T** (50 mg, 26 μmol) was converted to hexasaccharide **10** as a syrup (19 mg, 18 μmol, 69%). Characterization of **10** by ^{1}H and ^{13}C NMR is found in the Supporting Information. HRMS (ESI-TOF) *m/z* [M + Na]$^{+}$: calcd for $C_{39}H_{67}NO_{31}Na$, 1068.9288; found, 1068.9265.

E. Preparation of ^{13}C-Labeled Compounds **3**, **5–6** and **8–10**. Sixteen ^{13}C isotopomers of **3**, **5–6** and **8–10**, either singly- or doubly-labeled, were prepared as summarized in Scheme 3 of the text. These compounds were prepared using D-[1-^{13}C]mannose (13, 17), D-[2-^{13}C]mannose (13, 17), *N*-acetyl-D-[1-^{13}C]glucosamine (18), and *N*-acetyl-D-[2-^{13}C]glucosamine (18) as appropriate in the synthetic routes discussed above; see the indicated references for the preparation of the ^{13}C-labeled monosaccharides (~99-atom% ^{13}C at the labeled carbon; prepared by Omicron Biochemicals, Inc., South Bend, IN). Each ^{13}C-labeled di- and oligosaccharide was prepared in 25–50 mg quantities for subsequent use in *J*-coupling measurements by NMR. A list of the specific ^{13}C-isotopomers follows, with the superscript(s) on each compound number identifying the labeled carbon or carbons (see Scheme 3 in the text for definitions of the unprimed and primed residues in each compound).

Sixteen ^{13}C-Isotopomers of **3**, **5–6** and **8–10** That Were Prepared:

$\mathbf{3}^{1'}$; $\mathbf{3}^{2'}$

$\mathbf{5}^{1'}$; $\mathbf{5}^{2'}$; $\mathbf{5}^{1''}$; $\mathbf{5}^{2''}$

$\mathbf{6}^{1'}$; $\mathbf{6}^{2'}$; $\mathbf{6}^{1''}$; $\mathbf{6}^{2''}$

$\mathbf{8}^{1''}$; $\mathbf{8}^{2''}$; $\mathbf{8}^{1',1'''}$; $\mathbf{8}^{2',2'''}$

$\mathbf{9}^{1'}$

$\mathbf{10}^{1''}$

Table S1. Intra-Residue ^{1}H-^{1}H Spin-Couping Constants[a] in Disaccharide **3**, and Oligosaccharides **5**, **6** and **8**.

cmpd. no. / residue		^{1}H-^{1}H spin-coupling constants (Hz)						
		$^{3}J_{H1,H2}$	$^{3}J_{H2,H3}$	$^{3}J_{H3,H4}$	$^{3}J_{H4,H5}$	$^{3}J_{H5,H6a}$	$^{3}J_{H5,H6b}$	$^{2}J_{H6a,H6b}$
**3**	βMan	1.0	3.2	9.4	9.6	2.2	6.1	–12.1
	βGlcNAc′	8.5	10.4	8.8	9.7	2.3	6.1	–12.4
**5**	βMan	0.9	3.0	9.1	9.4	6.0	2.1	–11.5
	βGlcNAc′	8.4	10.5	8.8	9.9	2.2	6.1	–12.4
	αMan″	1.8	3.4	9.5	10.0	2.1	6.6	–12.0
**6**	βMan	1.0	3.0	9.4	9.4	2.1	5.9	–12.1
	αMan′	1.6	3.4	9.6	9.7	~1.8	6.3	–11.8
	βGlcNAc″	8.3	10.5	8.8	9.9	2.0	7.1	–12.4
**8**	βMan	0.9	3.2	9.5	9.6	*obsc*	*obsc*	*obsc*
	αMan′	1.7	3.4	9.5	9.8	*obsc*	*obsc*	*obsc*
	βGlcNAc″	8.3	10.5	8.6	*obsc*	1.9	*obsc*	–12.3
	αMan‴	1.8	3.4	9.5	10.0	2.1	6.9	–11.9

[a]In Hz ± 0.1 Hz; in D_2O at 22 °C. In each residue, H6a is defined as the less shielded H6 hydrogen; *obsc* denotes values that could not be measured due to signal overlap; $^{2}J_{HH}$ values were assumed to have negative signs. See Scheme 3 in the text for the identification of the unprimed and primed residues.

Table S2. Intra-Residue ^{13}C-^{13}C Spin-Coupling Constants[a] in Disaccharide **3** and Oligosaccharides **5**, **6** and **8**.

cmpd. no. / residue		^{13}C-^{13}C spin-coupling constants (Hz)					
		$^{1}J_{C1,C2}$	$^{2}J_{C1,C3}$	$^{2}J_{C1,C5}$	$^{3}J_{C1,C6}$	$^{1}J_{C2,C3}$	$^{2}J_{C2,C4}$
**3**	βGlcNAc′	45.5	+4.7	~0	4.0	37.1	+2.4
**5**	βGlcNAc′	45.5	+4.6	~0	3.9	37.2	+2.4
	αMan″	47.3	~0[b]	–1.9	3.1	37.8	~0
**6**	αMan′	47.2	~0	–2.0	3.1	~37.8	~0
	βGlcNAc″	45.3	+4.5	~0	4.2	37.2	+2.2
**8**	αMan′	47.2	~0	–1.9	3.0	~37.7	*obsc*
	βGlcNAc″	45.3	+4.5	~0	4.2	37.1	+2.1
	αMan‴	47.3	~0	–1.9	3.1	~37.8	*obsc*

[a]In Hz ± 0.1 Hz; in D_2O at 22 °C. An *obsc* entry denotes values that could not be measured due to signal overlap. See Scheme 3 in the text for the identification of the unprimed and primed residues. Signs of the $^{2}J_{CC}$ values were assumed to be the same as those in either methyl 2-acetamido-2-deoxy-β-D-glucopyranoside (ref. 18) or methyl/ethyl α-D-mannopyran-oside (ref. 19). [b]Values shown as ~ 0 Hz are 0.5 Hz or less.

Table S3. ^{1}H and ^{13}C Chemical Shifts[a] in Disaccharide **3** and Oligosaccarides **5**, **6** and **8**.

**compound/ residue**		^{1}**H chemical shifts (ppm)**								
		H1	H2	H3	H4	H5	H6a	H6b	OCH_3	$COCH_3$
**3**	βMan	4.555	4.007	3.729	3.672	3.417	3.833	3.650	3.514	
	βGlcNAc′	4.524	3.728	3.544	3.441	3.498	3.920	3.729		2.051
**5**	βMan	4.546	4.011	3.737	3.707	3.547	3.824	3.788	3.506	
	βGlcNAc′	4.525	3.728	3.547	3.442	3.492	3.914	3.734		2.040
	αMan″	4.910	3.982	3.797	3.627	3.695	3.934	3.753		
**6**	βMan	4.539	4.050	3.870	3.893	3.373	3.881	3.668	3.500	
	αMan′	5.217	4.131	3.842	3.630	3.693	3.865	3.723		
	βGlcNAc″	4.473	3.672	3.518	3.368	3.414	3.950	3.712		2.032
**8**	βMan	4.516	4.042	3.854	3.938	3.490	~3.82	~3.82	3.475	
	αMan′	5.207	4.120	3.835	3.619	~3.68	~3.84	~3.71		
	βGlcNAc″	4.487	3.669	3.508	3.357	~3.38	3.945	~3.70		2.014
	αMan‴	4.928	3.978	3.765	3.603	~3.67	3.928	3.734		

**compound/ residue**		^{13}**C chemical shifts (ppm)**								
		C1	C2	C3	C4	C5	C6	OCH_3	*C*OCH_3	CO*C*H_3
**3**	βMan	103.57	72.36	74.41	80.18	77.43	63.17	59.50		
	βGlcNAc′	104.27	58.24	76.11	72.45	78.57	63.28		177.27	24.79
**5**	βMan	103.55	72.23	74.31	79.76	76.21	68.72	59.53		
	βGlcNAc′	104.09	58.32	76.06	72.49	78.65	63.30		177.25	24.89
	αMan″	102.80	72.57	73.13	69.49	75.80	63.82			
**6**	βMan	103.45	72.73	79.86	76.52	77.99	62.99	59.50		
	αMan′	104.38	72.46	73.08	69.52	76.05	63.71			
	βGlcNAc″	103.85	58.77	76.17	72.89	78.86	64.19		177.28	24.83
**8**	βMan	103.42	72.51	79.82	76.09	76.59	68.43	59.49	177.27	24.82
	αMan′	104.37	72.47	73.05	69.49	76.01	63.68			
	βGlcNAc″	103.65	58.77	76.08	72.94	79.10	64.16			
	αMan‴	102.83	72.53	73.20	69.50	75.94	63.85			

[a]In D_2O at 22 °C. Chemical shifts are given in ppm relative to external DSS; ± 0.001 ppm for ^{1}H, ± 0.01 ppm for ^{13}C. In each residue, H6a is defined as the less shielded H6 hydrogen. See Scheme 3 in the text for the identification of the unprimed and primed residues.

Table S4. ^{1}H and ^{13}C Chemical Shifts[a] in Oligosaccharides **9** and **10**.

**compound/ residue**		**^{1}H chemical shifts (ppm)**								
		H1	H2	H3	H4	H5	H6a	H6b	OCH_3	$COCH_3$
**9**	βMan	4.544	4.009	~3.72	~3.72	3.534	3.821	3.795	3.508	
	βGlcNAc′	4.518	3.721	3.550	3.440	3.493	3.919	3.734		2.059
	αMan”	5.156	4.021	3.913	~3.67	~3.67	3.932	3.752		
	αMan’’’	5.026	4.046	3.826	3.606	~3.77	3.886	3.711		
**10**	βMan	4.519	4.028	3.791	3.976	3.466	~3.85	~3.83	3.49	
	βGlcNAc′	4.469	3.803	3.505	3.340	3.400	3.976	~3.76		2.030
	αMan’	5.197	4.045	3.656	3.890	3.661	3.954	~3.70		
	αMan’’’	5.030	4.037	3.815	3.584	3.750	3.893	~3.76		
	αMan’’’’	5.628	4.190	3.936	3.648	3.658	3.856	3.725		
	αMan’’’’’	5.044	4.011	3.806	3.611	3.813	3.882	3.784		
**compound/ residue**		**^{13}C chemical shifts (ppm)**								
		C1	C2	C3	C4	C5	C6	OCH_3	*C*OCH_3	CO*C*H_3
**9**	βMan	103.62	72.24	74.31	79.55	76.05	68.69	59.56		
	βGlcNAc′	104.04	58.32	76.01	72.49	78.66	63.29		177.27	24.94
	αMan”	101.13	80.99	72.84	69.63	75.94	63.83			
	αMan’’’	104.83	72.61	72.97	69.53	75.99	63.81			
**10**	βMan	103.55	72.57	79.69	75.82	76.54	68.39	59.53		
	βGlcNAc′	103.98	58.77	76.62	73.17	79.21	63.95		177.47	24.82
	αMan’	101.31	80.77	72.96	69.64	76.19	63.88			
	αMan’’’	104.76	72.59	72.96	69.57	76.05	63.92			
	αMan’’’’	102.45	82.45	72.37	69.86	75.82	63.80			
	αMan’’’’’	105.11	72.88	73.20	69.59	75.44	63.46			

[a]In D_2O at 22 °C. Chemical shifts are given in ppm relative to external DSS; ± 0.001 ppm for ^{1}H, ± 0.01 ppm for ^{13}C. In each residue, H6a is defined as the less shielded H6 hydrogen. See Scheme 3 in the text for the identification of the unprimed and primed residues.

Representative ^{1}H and $^{13}C\{^{1}H\}$ NMR Spectra of Synthetic Intermediates and Products

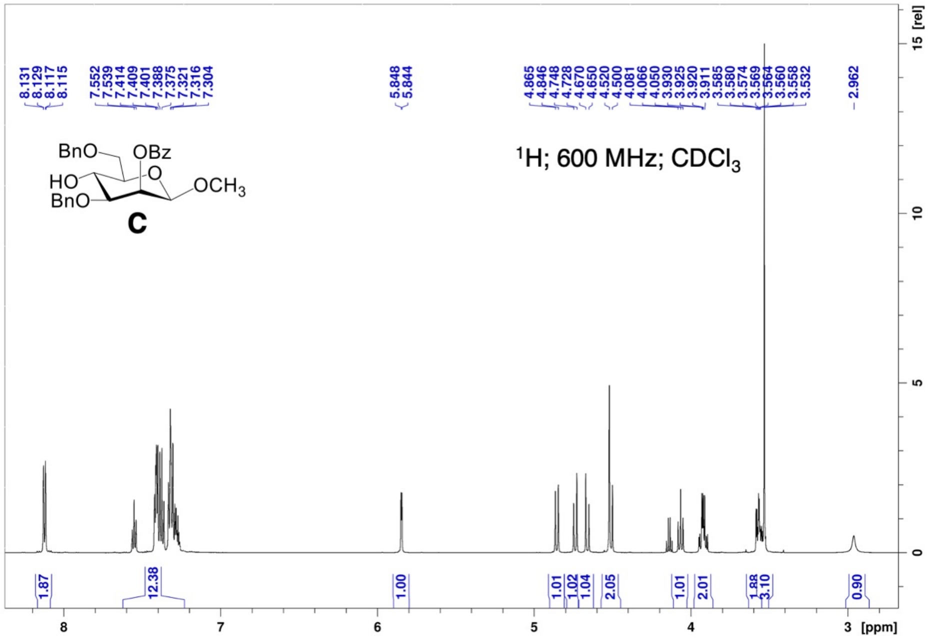

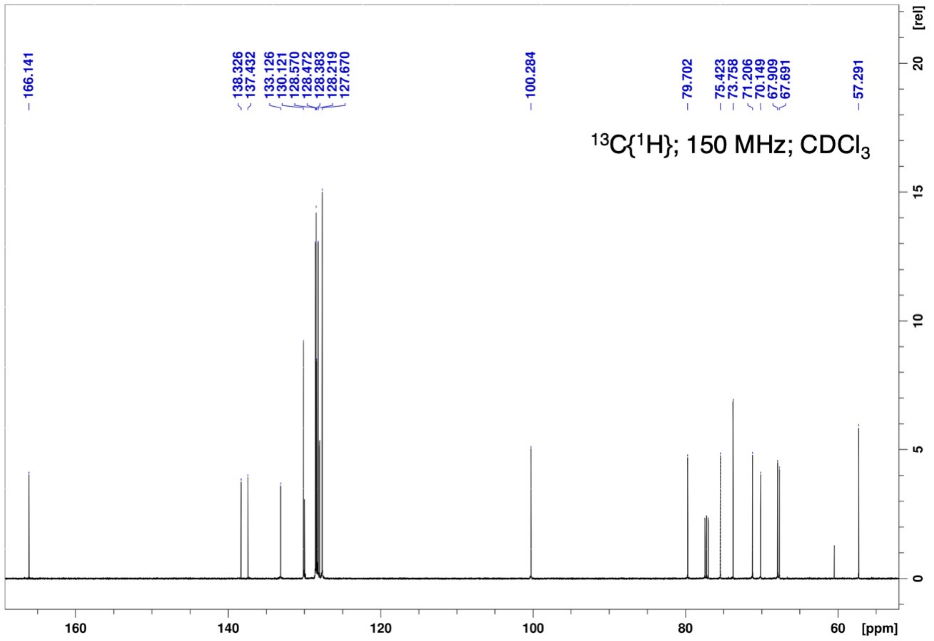

Figure S1. ^{1}H and $^{13}C\{^{1}H\}$ NMR spectra of intermediate **C** in $CDCl_3$.

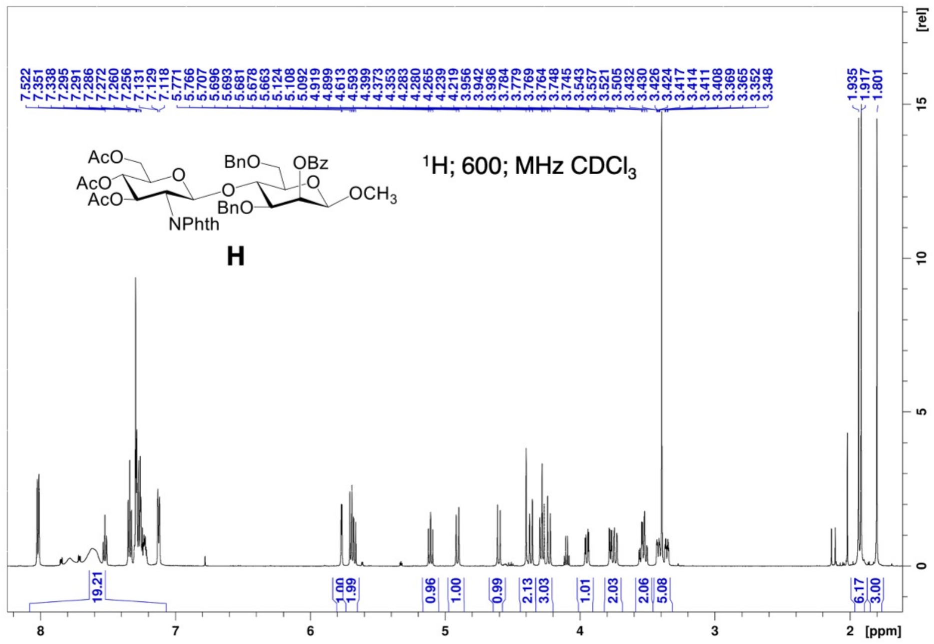

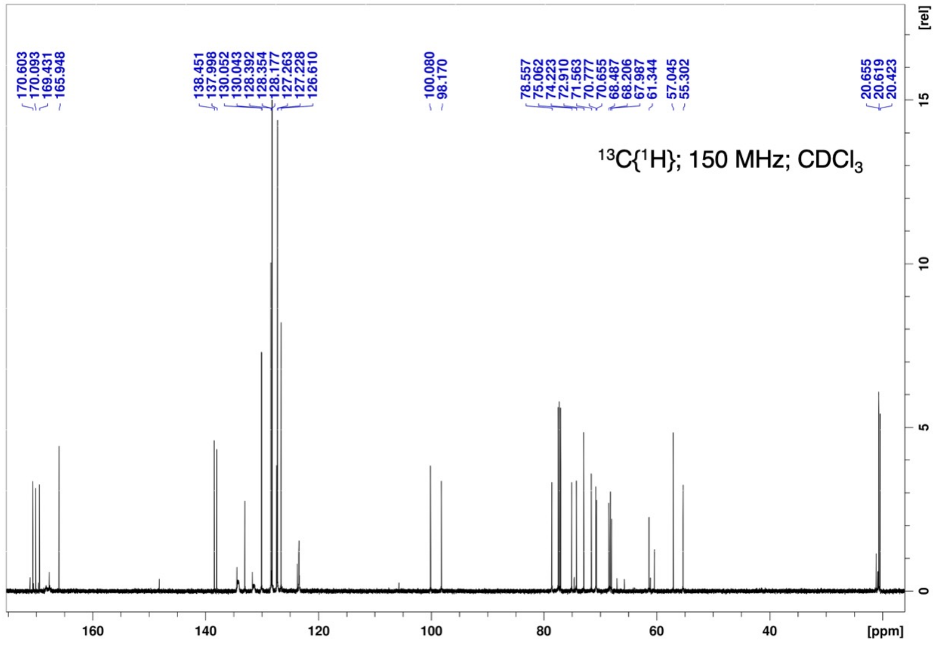

Figure S2. ^{1}H and ^{13}C{^{1}H} NMR spectra of intermediate **H** in CDCl$_3$.

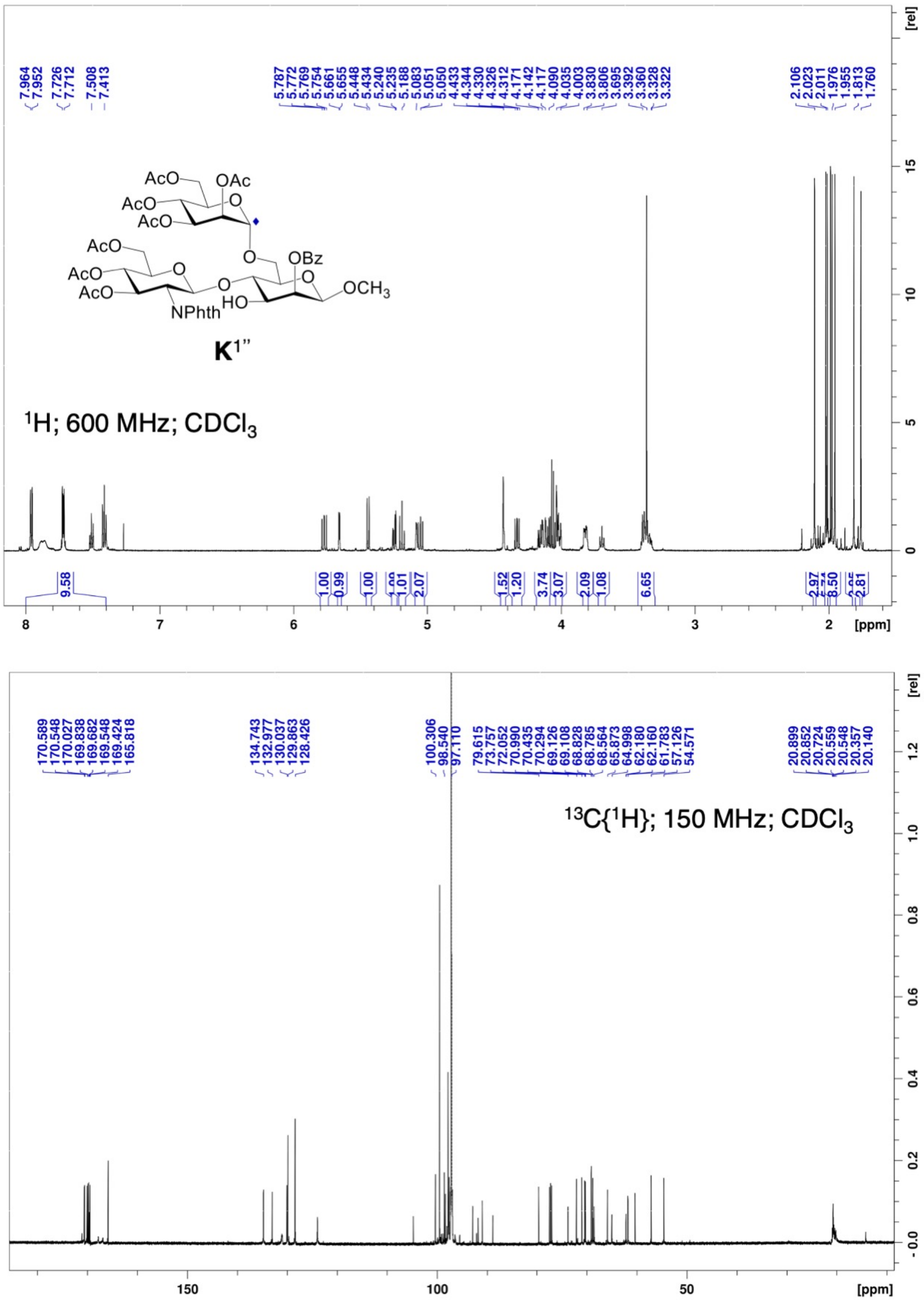

Figure S3. ^{1}H and $^{13}C\{^{1}H\}$ NMR spectra of intermediate **K**$^{1''}$ in $CDCl_3$.

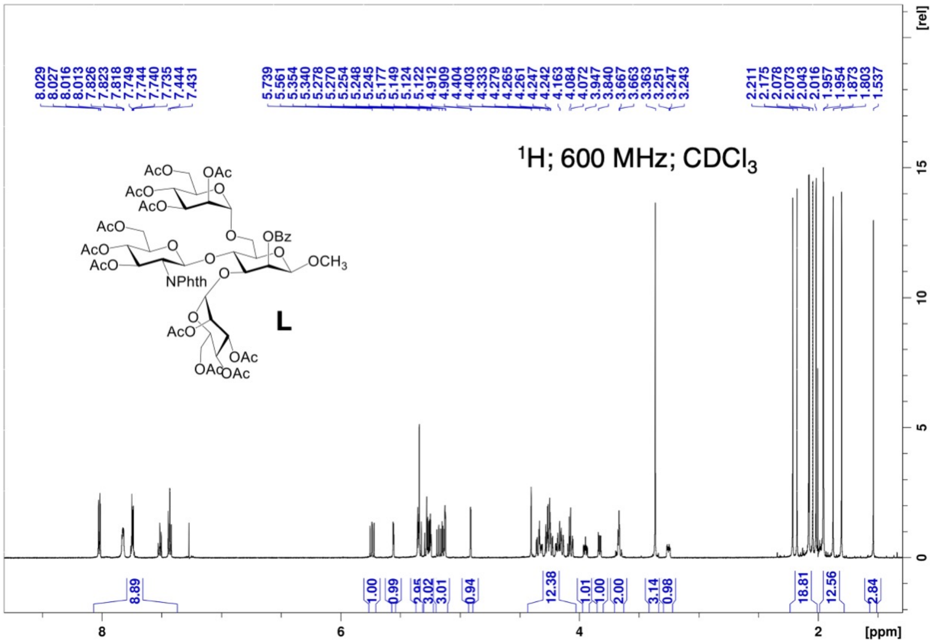

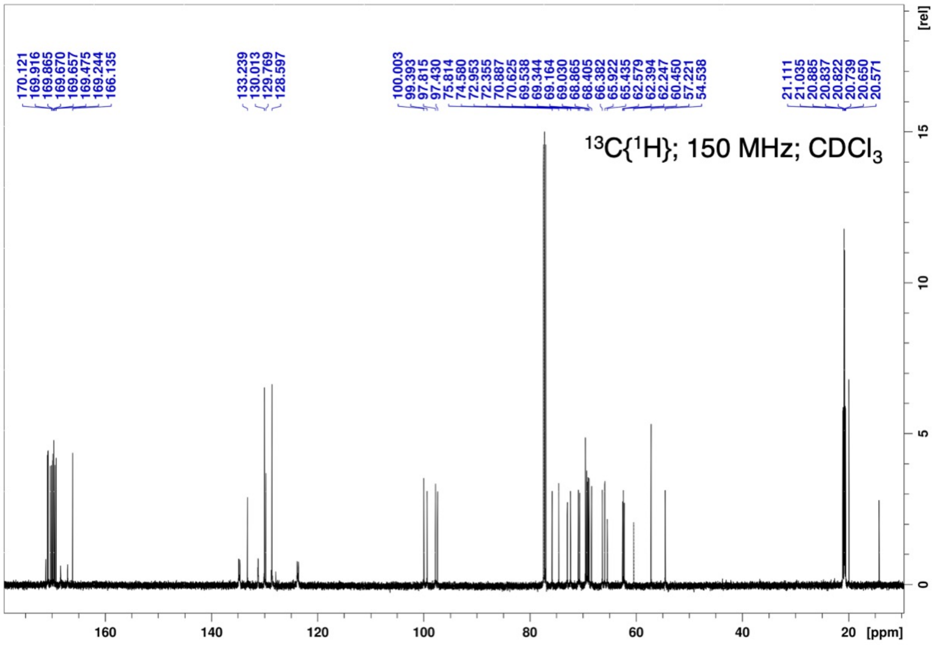

Figure S4. ^{1}H and ^{13}C{^{1}H} NMR spectra of intermediate **L** in $CDCl_3$.

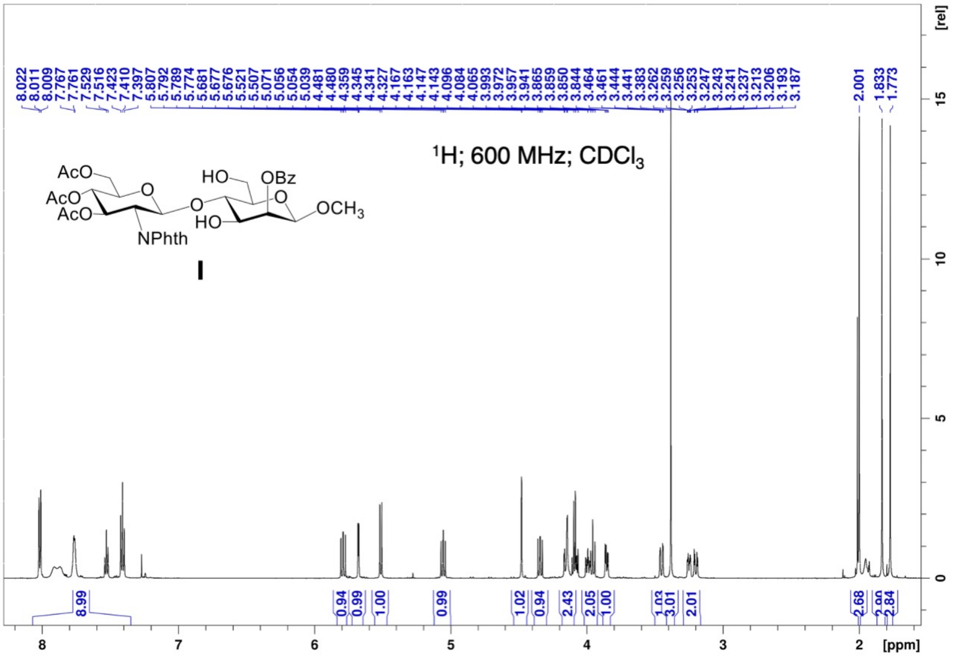

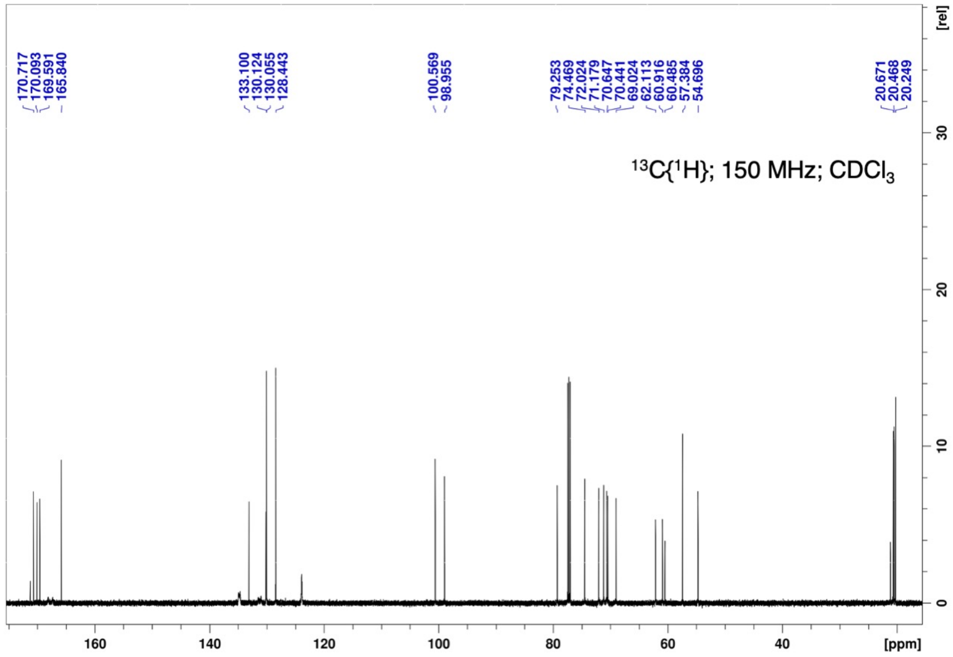

Figure S5. ^{1}H and $^{13}C\{^{1}H\}$ NMR spectra of intermediate **I** in $CDCl_3$.

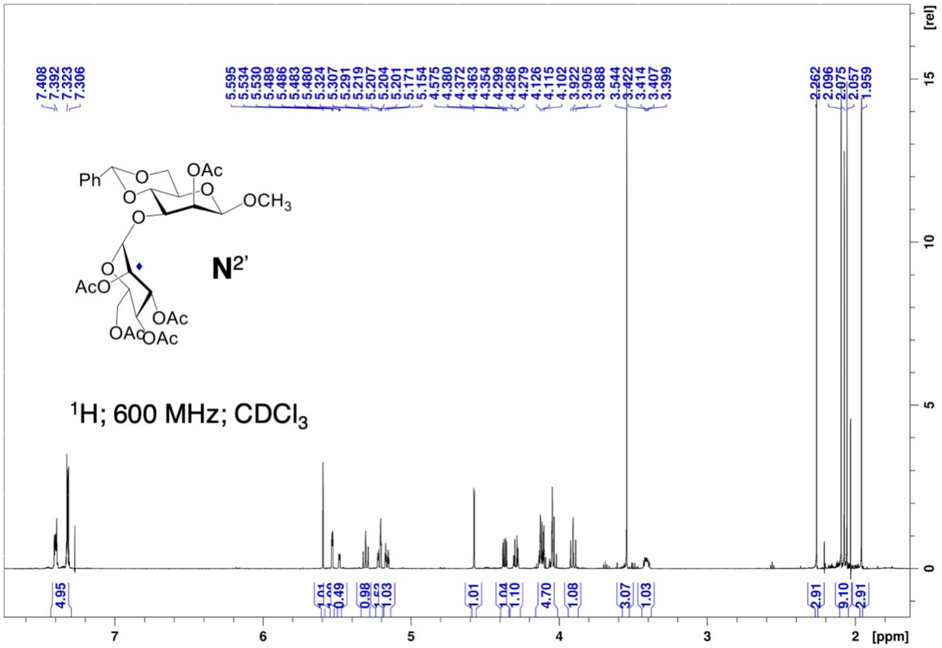

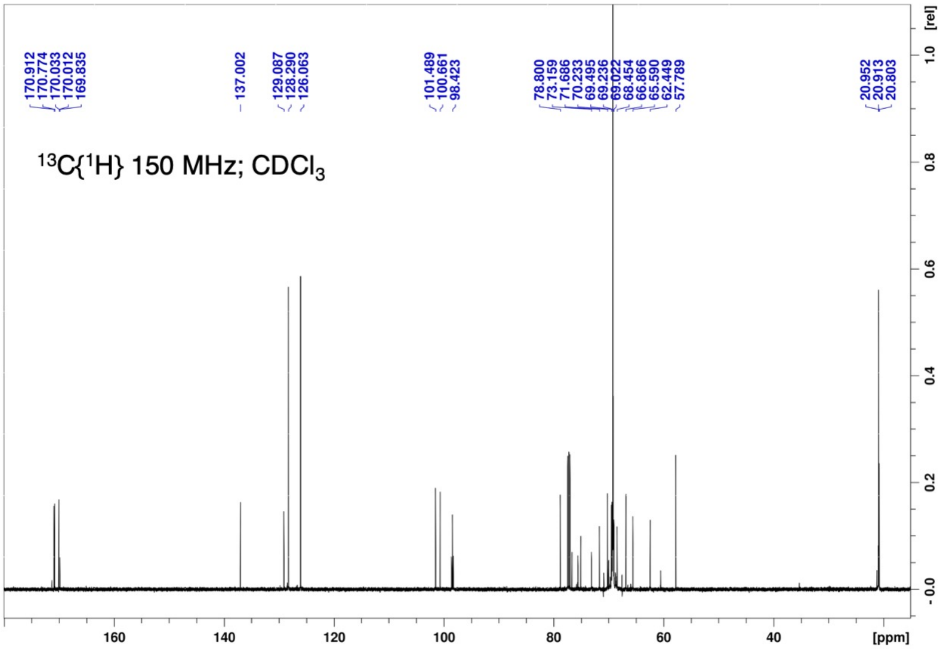

Figure S6. ^{1}H and $^{13}C\{^{1}H\}$ NMR spectra of intermediate $\mathbf{N}^{2'}$ in $CDCl_3$.

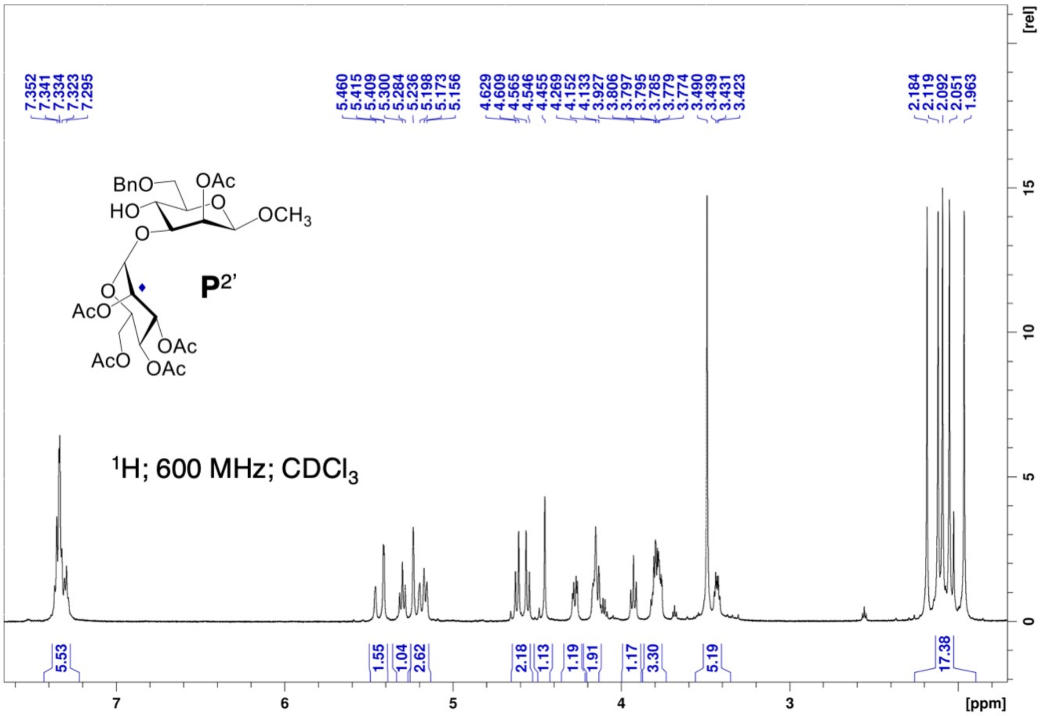

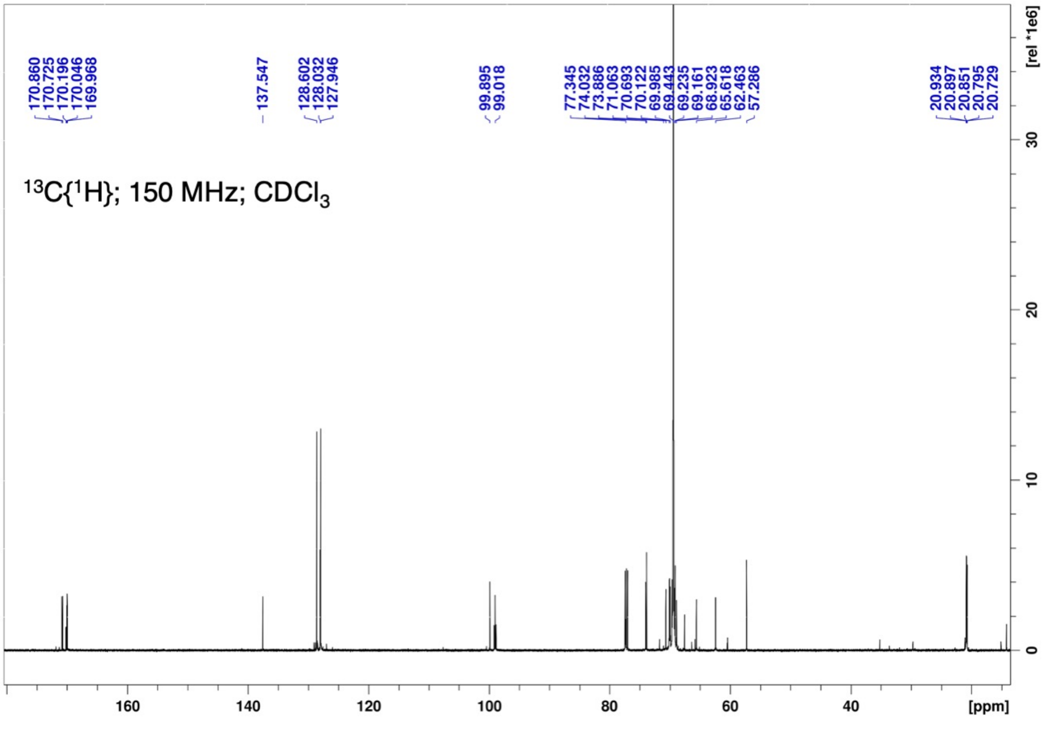

Figure S7. ^{1}H and $^{13}C\{^{1}H\}$ NMR spectra of intermediate $\mathbf{P}^{2'}$ in $CDCl_3$.

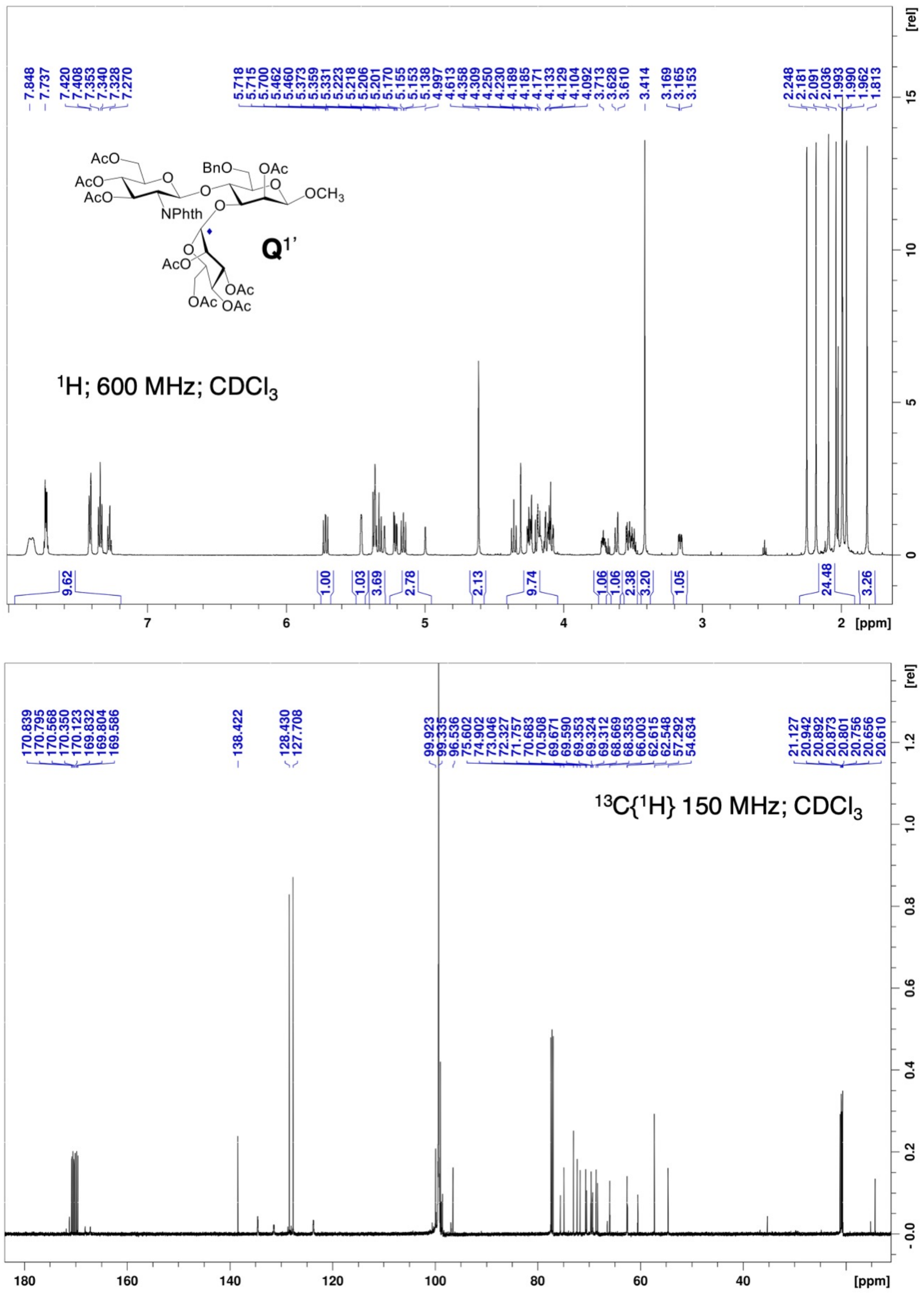

Figure S8. ^{1}H and $^{13}C\{^{1}H\}$ NMR spectra of intermediate $\mathbf{Q}^{1'}$ in $CDCl_3$.

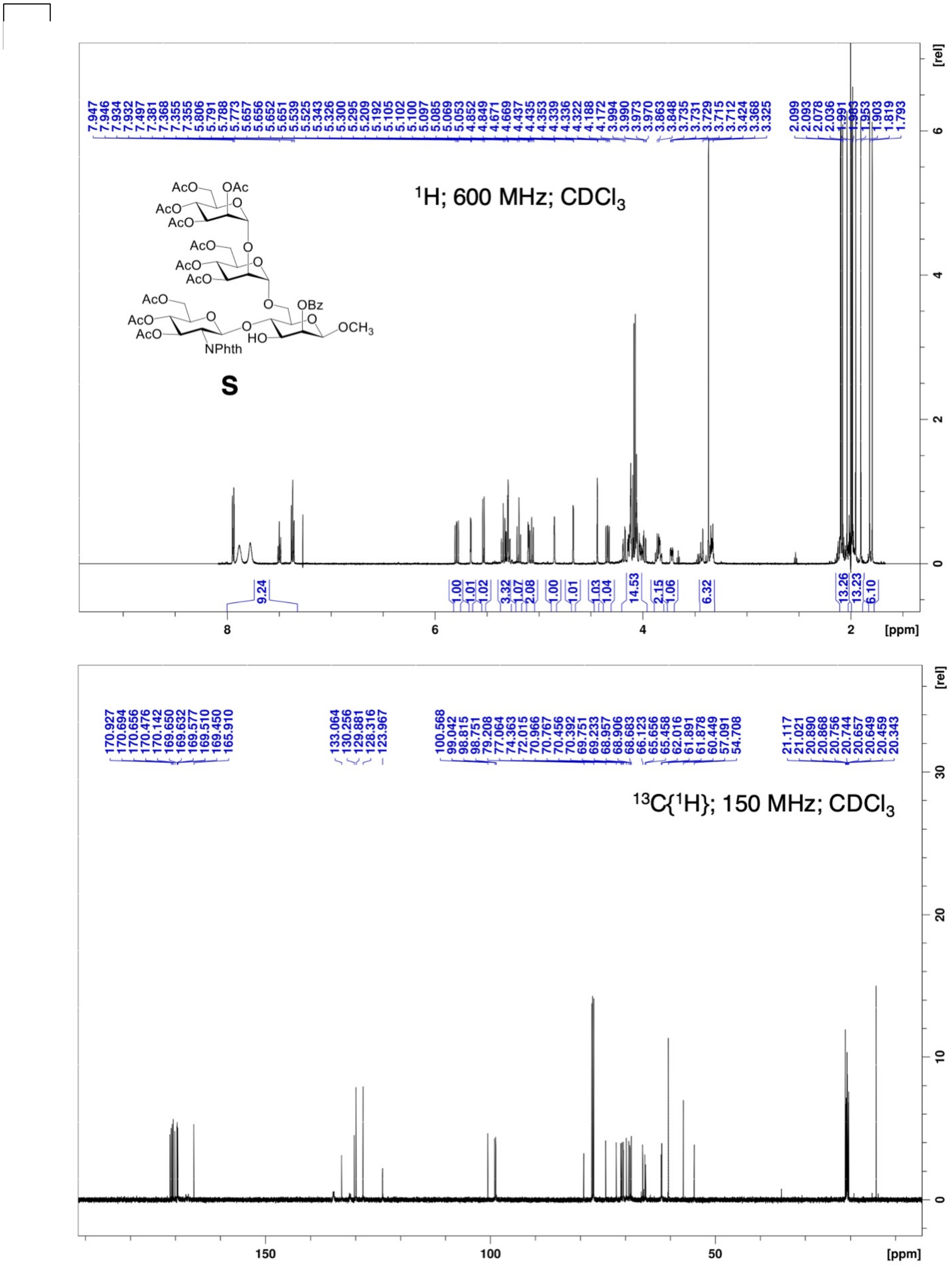

Figure S9. ^{1}H and $^{13}C\{^{1}H\}$ NMR spectra of intermediate **S** in $CDCl_3$.

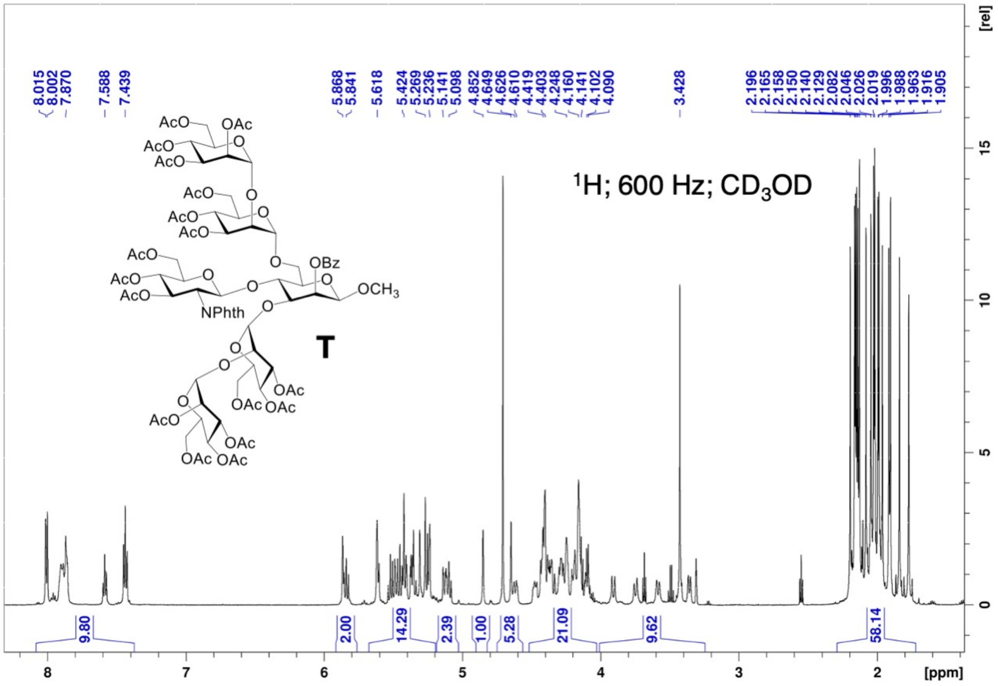

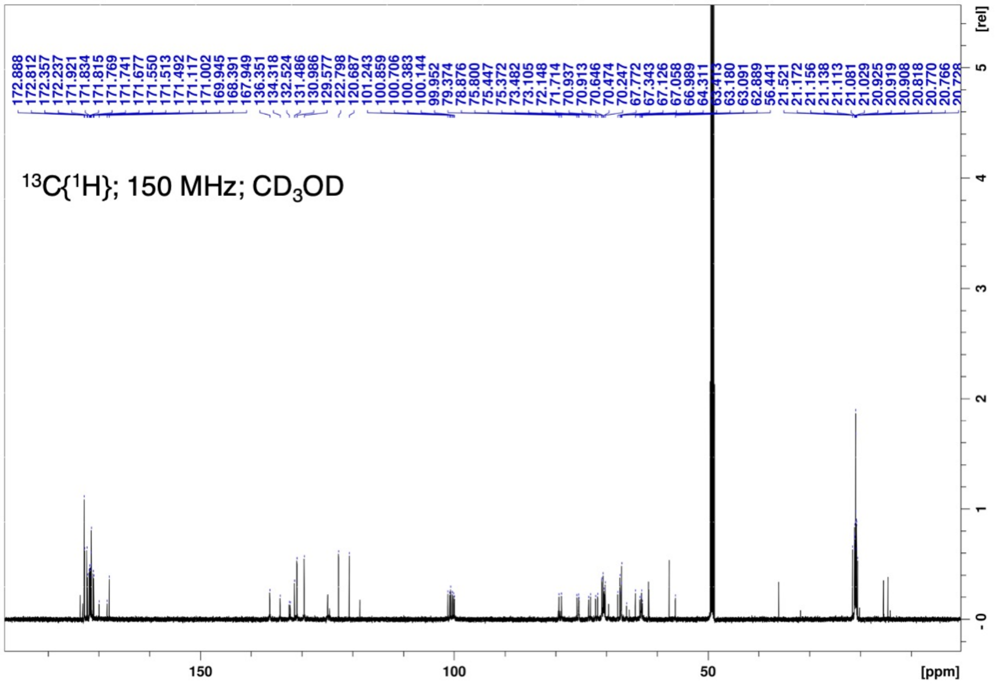

Figure S10. ^{1}H and ^{13}C{^{1}H} NMR spectra of intermediate **T** in $CDCl_3$.

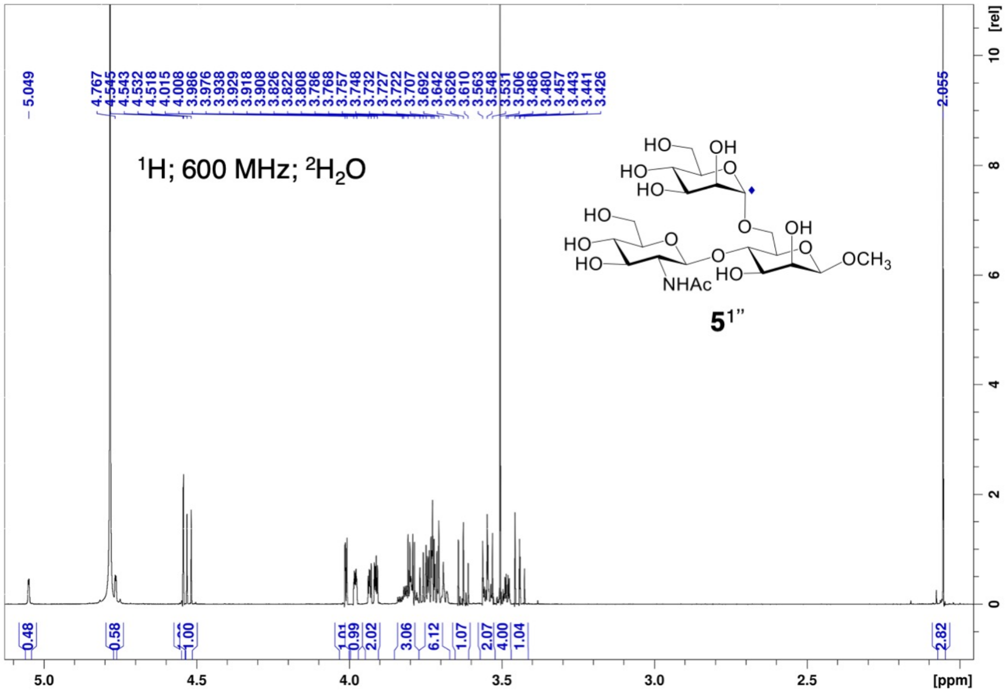

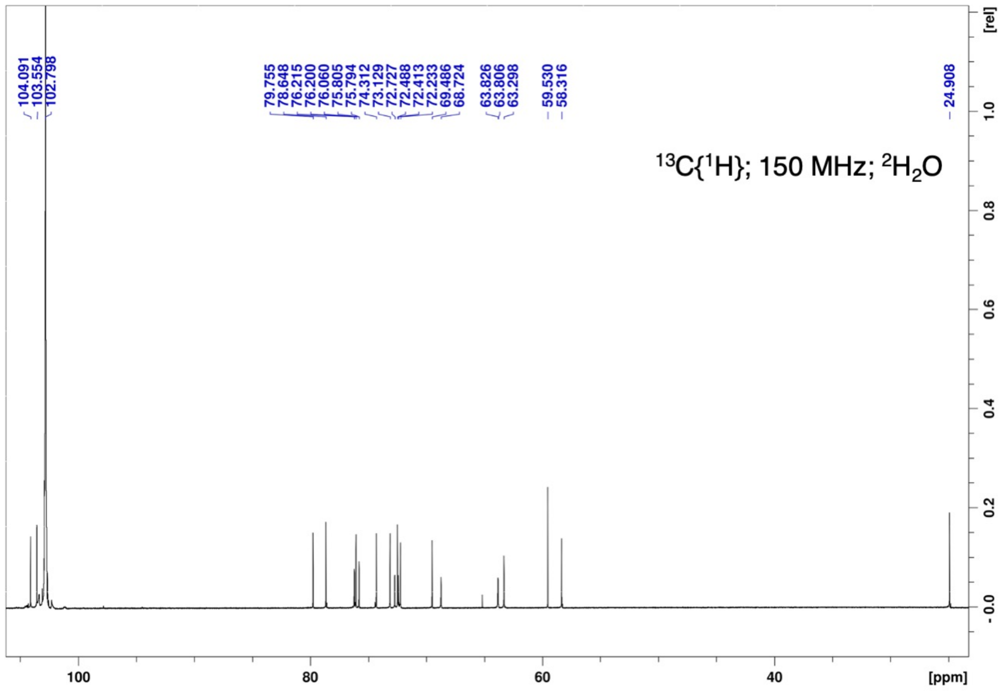

Figure S11. ^1H and $^{13}C\{^1H\}$ NMR spectra of product **5**$^{1''}$ in ^2H_2O.

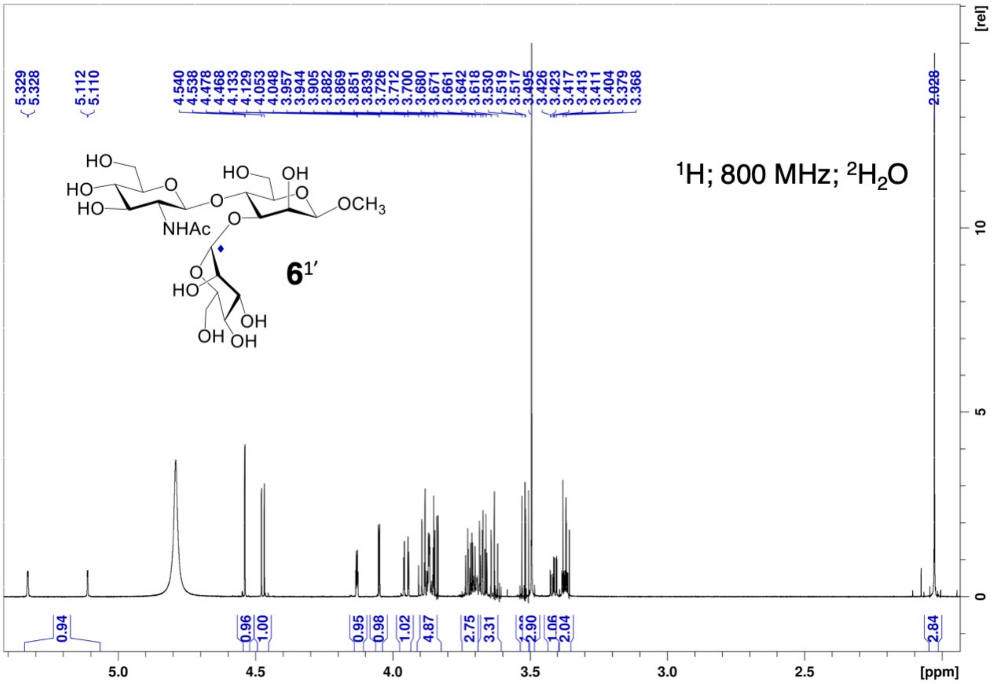

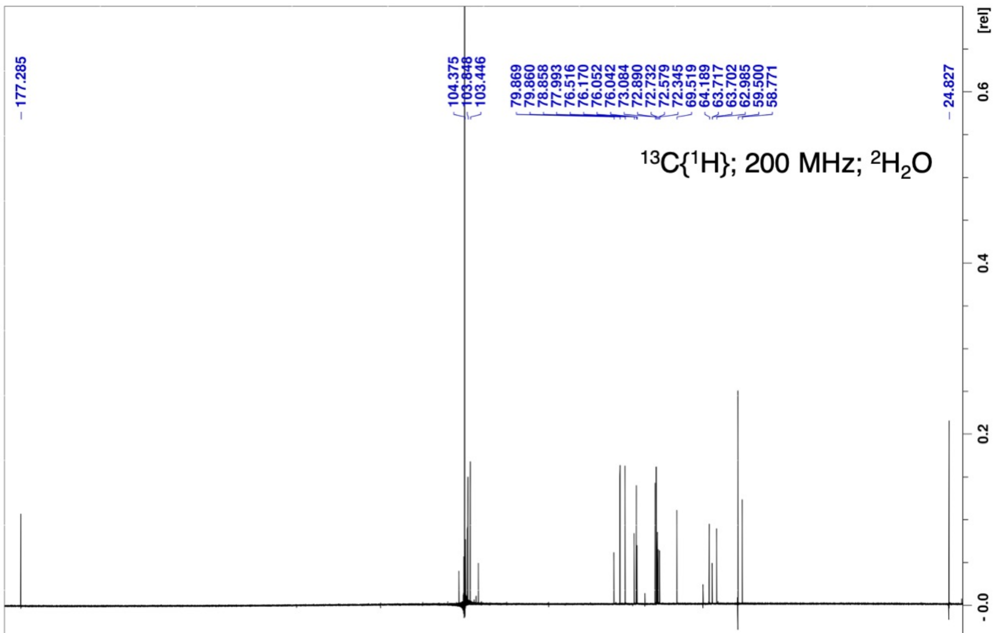

Figure S12. ^1H and $^{13}C\{^1H\}$ NMR spectra of product **6**$^{1'}$ in ^2H_2O.

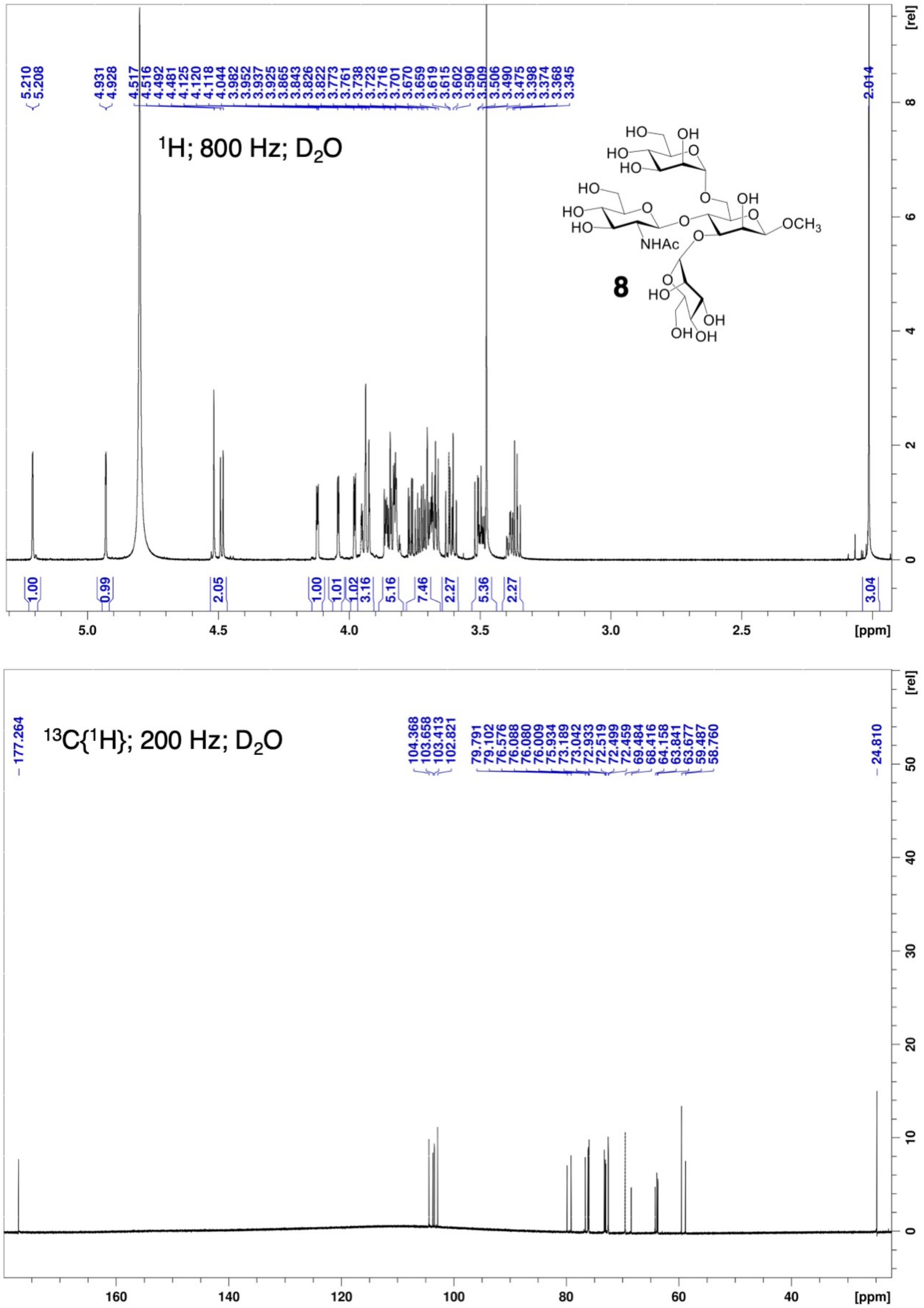

Figure S13. ^{1}H and ^{13}C{^{1}H} NMR spectra of product **8** in $^{2}H_2O$.

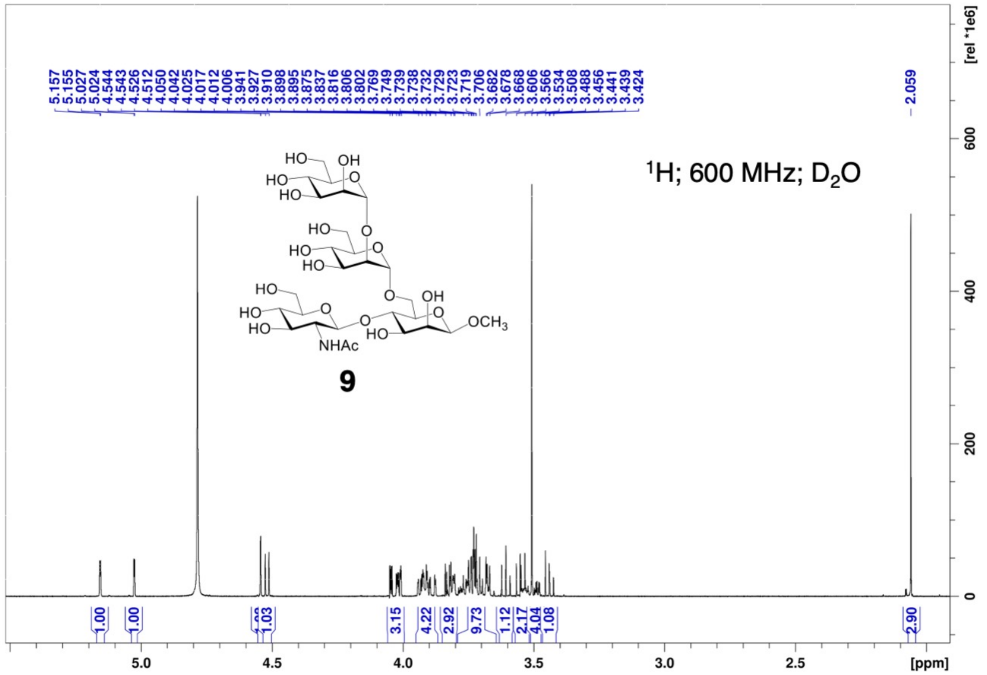

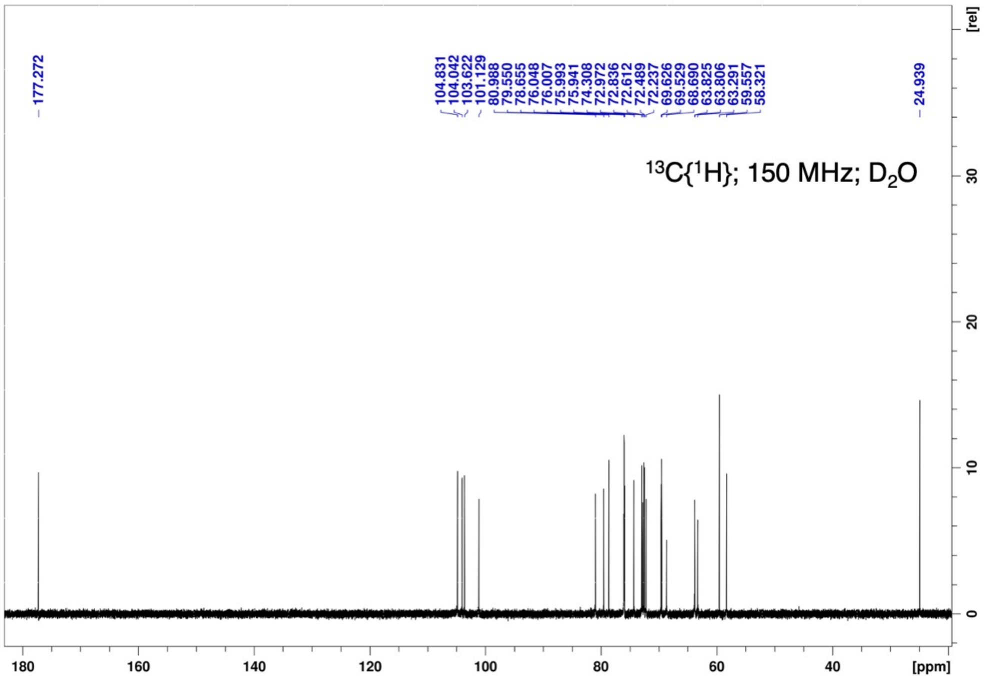

Figure S14. ^{1}H and $^{13}C\{^{1}H\}$ NMR spectra of product **9** in $^{2}H_2O$.

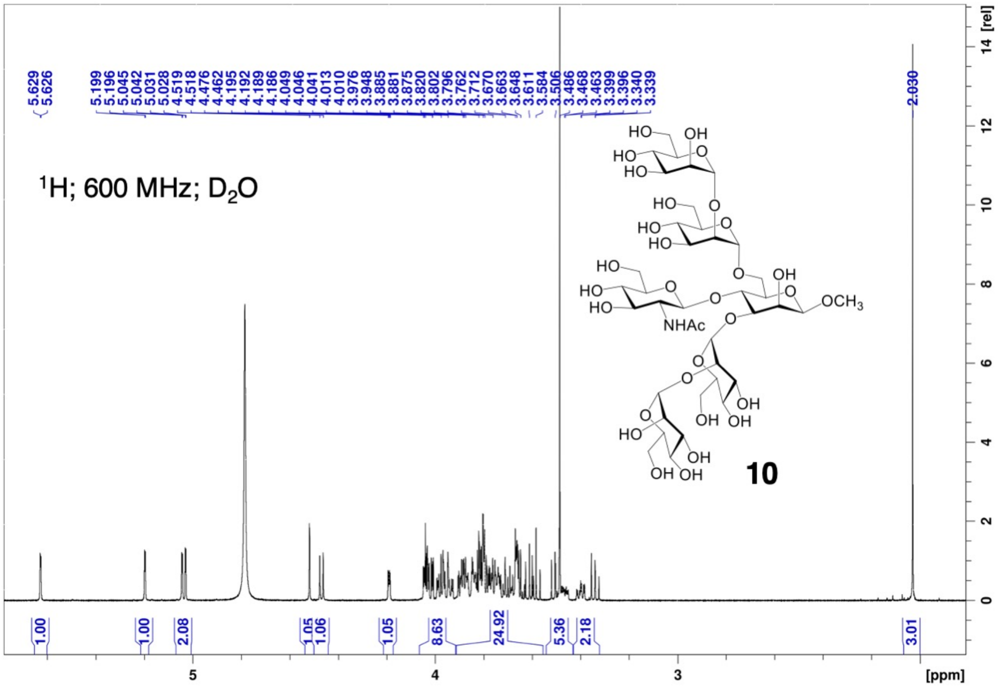

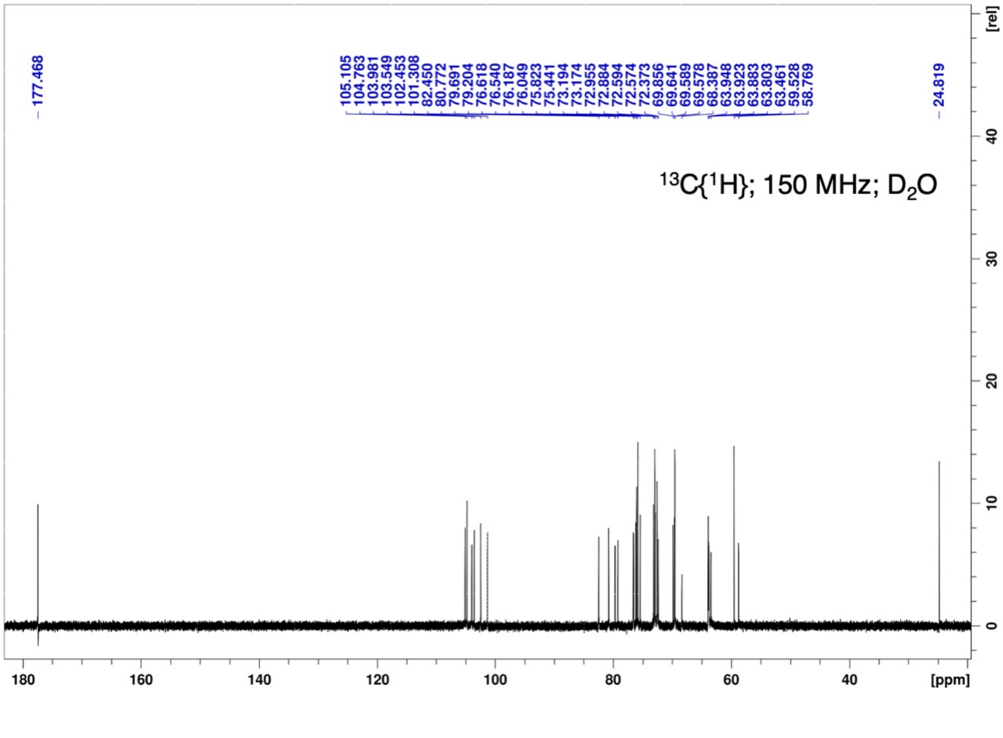

Figure S15. ^{1}H and ^{13}C{^{1}H} NMR spectra of product **10** in ^2H_2O.

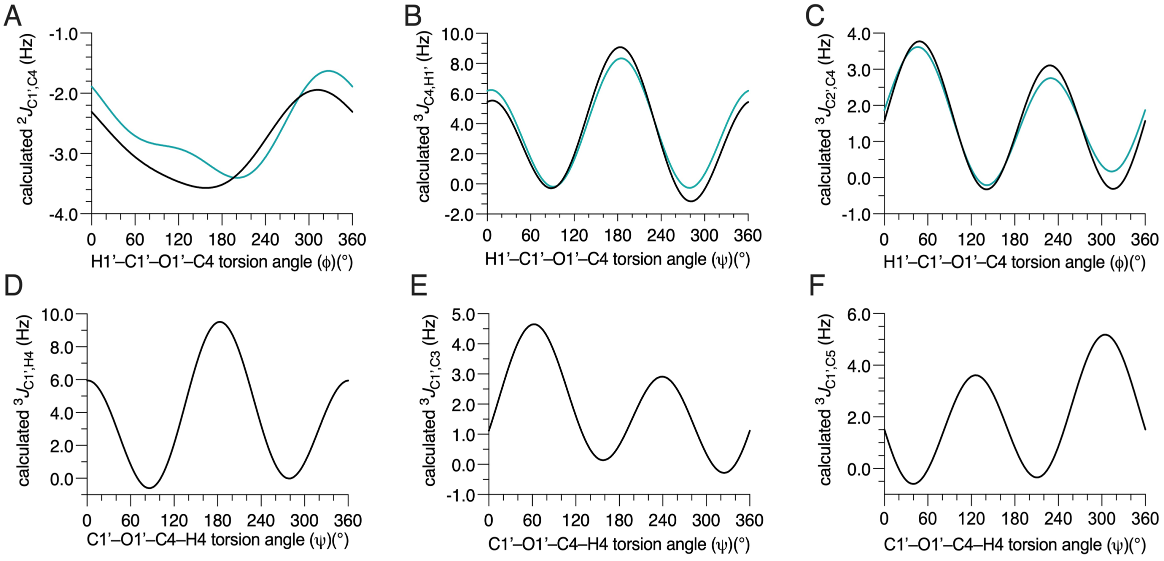

Figure S16. Plots of calculated trans-*O*-glycosidic *J*-couplings in disaccharide **3**^{c} as a function of ϕ (A–C) and ψ (D–F). (A) $^2J_{C1',C4}$. (B) $^3J_{C4,H1'}$. (C) $^3J_{C2',C4}$. (D) $^3J_{C1',H4}$. (E) $^3J_{C1',C3}$. (F) $^3J_{C1',C5}$. In A–C, green = restricted, black = trimmed; see the text for a discussion of restricted and trimmed equations.

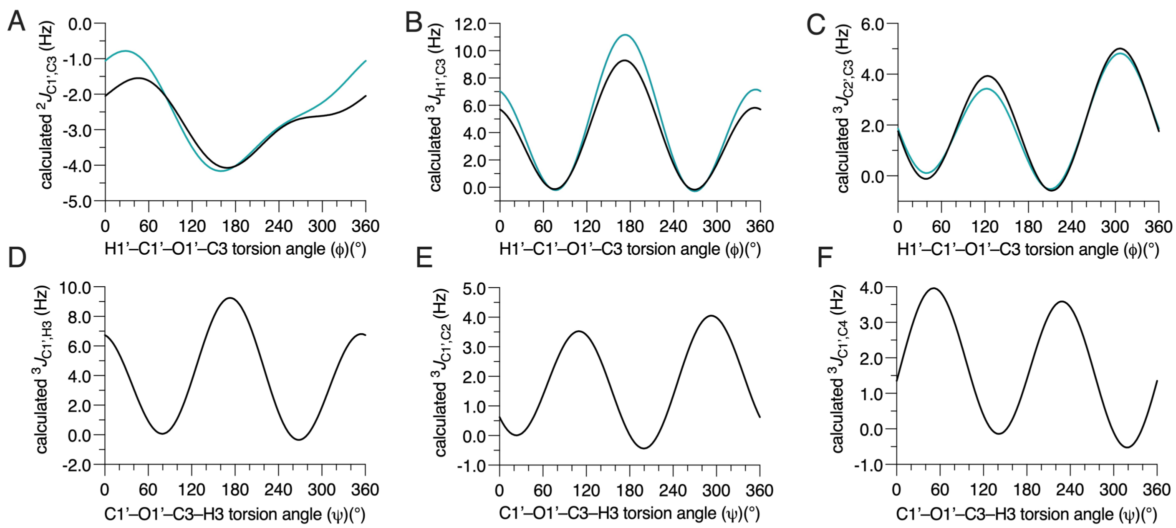

Figure S17. Plots of calculated trans-*O*-glycosidic *J*-couplings in disaccharide **4**[c] as a function of ϕ (A–C) and ψ (D–F). (A) $^{2}J_{C1',C3}$. (B) $^{3}J_{C3,H1'}$. (C) $^{3}J_{C2',C3}$. (D) $^{3}J_{C1',H3}$. (E) $^{3}J_{C1',C2}$. (F) $^{3}J_{C1',C4}$. In A–C, green = restricted, black = trimmed; see the text for a discussion of restricted and trimmed equations.

Parameterized Equations for ϕ- and ψ-Dependent J-Couplings in 3[c] and 4[c]

βGlcNAc-(1→4)-βMan linkage in 3[c]

$^{2}J_{C1',C4}$ (Hz) = $-2.63 + 0.69 \cos \phi - 0.21 \sin \phi - 0.26 \sin 2\phi$
RMSD = 0.31 Hz eq [S1]

$^{2}J_{C2',H1'}$ (Hz) = $2.46 + 0.43 \cos \phi + 0.06 \sin \phi - 1.06 \cos 2\phi - 0.85 \sin 2\phi$
RMSD = 0.37 Hz eq [S2]

$^{3}J_{C2',C4}$ (Hz) = $1.41 + 0.31 \cos \phi + 0.51 \sin \phi + 0.11 \cos 2\phi + 1.80 \sin 2\phi$
RMSD = 0.47 Hz eq [S3]

$^{3}J_{C4,H1'}$ (Hz) = $3.48 - 1.13 \cos \phi + 0.06 \sin \phi + 3.73 \cos 2\phi + 0.77 \sin 2\phi$
RMSD = 0.38 Hz eq [S4]

$^{3}J_{C1',C3}$ (Hz) = $1.88 + 0.24 \cos \psi + 0.86 \sin \psi - 1.00 \cos 2\psi + 1.62 \sin 2\psi$
RMSD = 0.56 Hz eq [S5]

$^{3}J_{C1',C5}$ (Hz) = $1.98 + 0.35 \cos \psi - 0.72 \sin \psi - 0.82 \cos 2\psi - 2.27 \sin 2\psi$
RMSD = 0.61 Hz eq [S6]

$^{3}J_{C1',H4}$ (Hz) = $3.76 - 1.77 \cos \psi - 0.36 \sin \psi + 3.96 \cos 2\psi + 0.28 \sin 2\psi$
RMSD = 0.63 Hz eq [S7]

αMan-(1→3)-βMan linkage in 4[c]

$^{2}J_{C1',C3}$ (Hz) = $-2.50 + 1.48 \cos \phi + 0.12 \sin \phi + 0.45 \sin 2\phi$
RMSD = 0.26 Hz eq [S8]

$^{3}J_{C2',C3}$ (Hz) = $1.97 + 0.65 \cos \phi - 0.39 \sin \phi - 0.76 \cos 2\phi - 2.01 \sin 2\phi$
RMSD = 0.42 Hz eq [S9]

$^{3}J_{C3,H1'}$ (Hz) = $4.51 - 1.98 \cos \phi + 0.28 \sin \phi + 4.51 \cos 2\phi - 1.13 \sin 2\phi$
RMSD = 0.32 Hz eq [S10]

$^{3}J_{C1',C2}$ (Hz) = $1.79 + 0.30 \cos \psi - 0.16 \sin \psi - 1.47 \cos 2\psi - 1.35 \sin 2\psi$
RMSD = 0.49 Hz eq [S11]

$^{3}J_{C1',C4}$ (Hz) = $1.72 + 0.27 \sin \psi - 0.34 \cos 2\psi - 2.02 \sin 2\psi$
RMSD = 0.48 Hz eq [S12]

$^{3}J_{C1',H3}$ (Hz) = $3.97 - 1.19 \cos \psi + 0.34 \sin \psi + 3.96 \cos 2\psi - 0.89 \sin 2\psi$
RMSD = 0.48 Hz eq [S13]

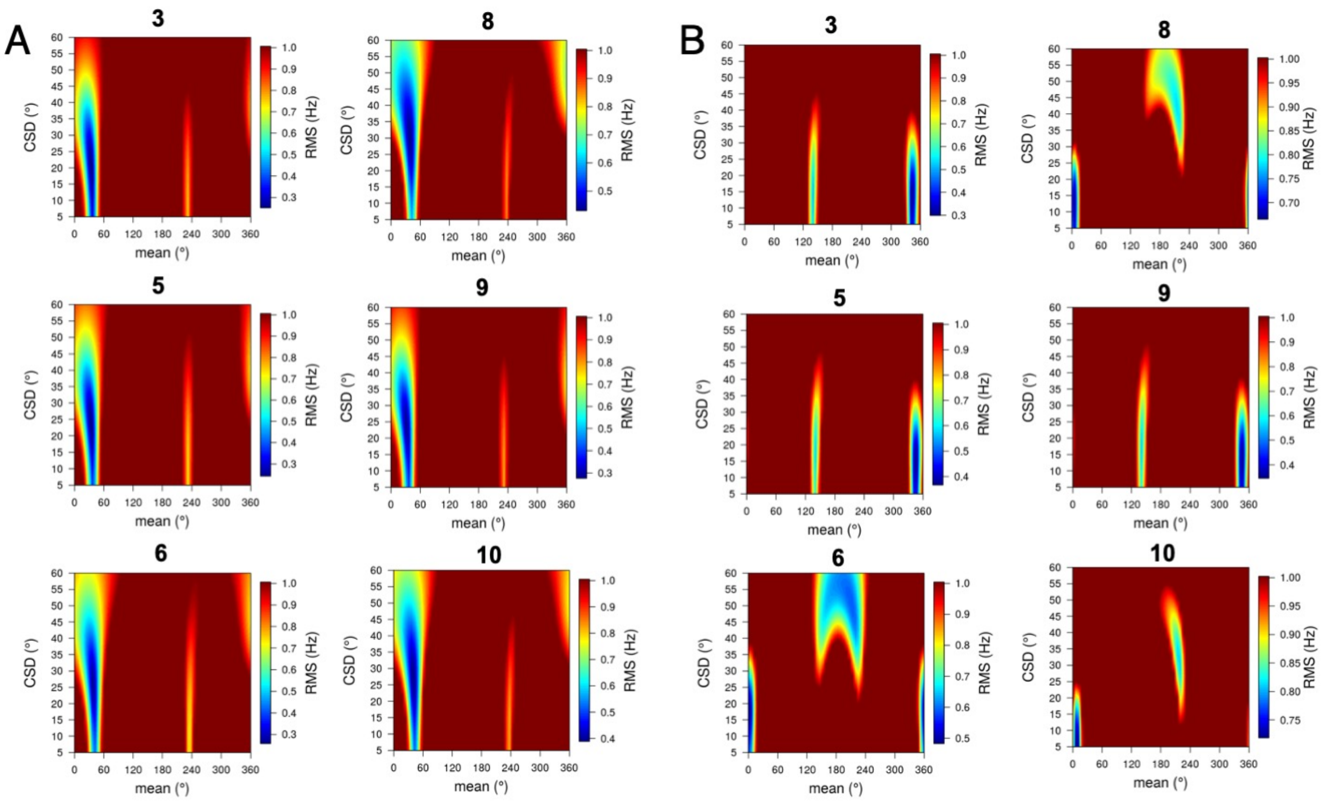

Figure S18. Parameter space plots for single-state models of ϕ (A) and ψ (B) for the βGlcNAc-(1→4)-βMan linkages in disaccharide **3**, trisaccharides **5** and **6**, tetrasaccharides **8** and **9**, and hexasaccharide **10**.

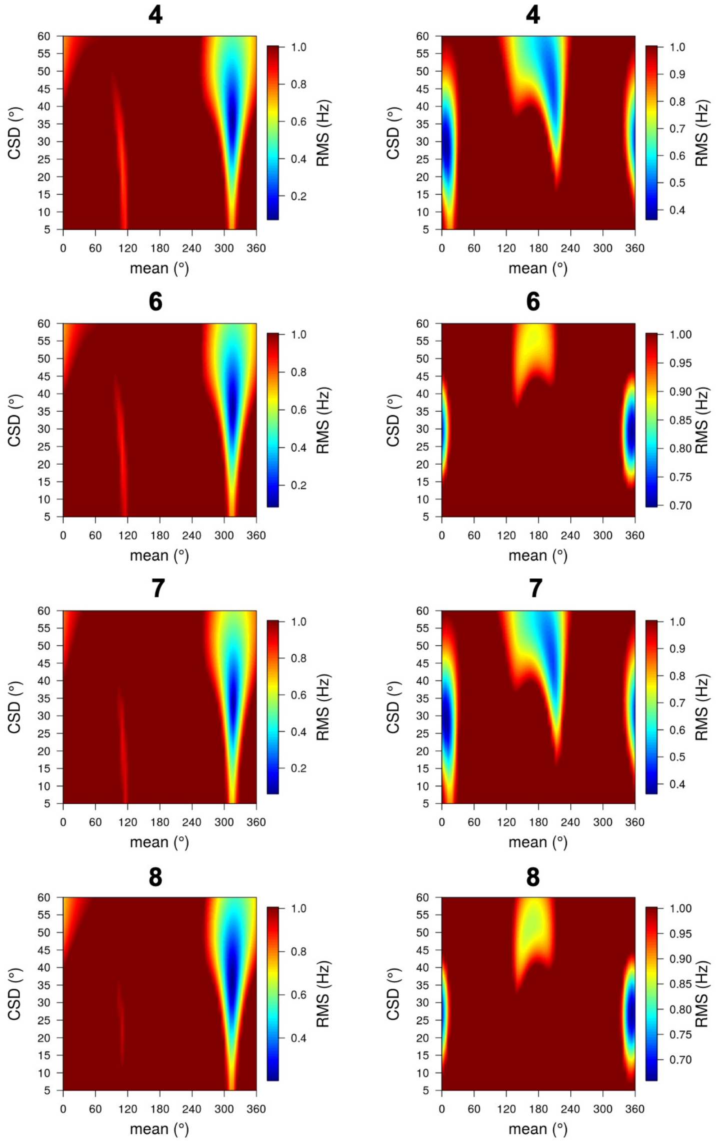

Figure S19. Parameter space plots for single-state models of ϕ and ψ for the αMan-(1→3)-βMan linkages in disaccharide **4**, trisaccharides **6** and **7**, and tetrasaccharide **8**. Plots for ϕ are shown on the left, and plots for ψ are shown on the right.

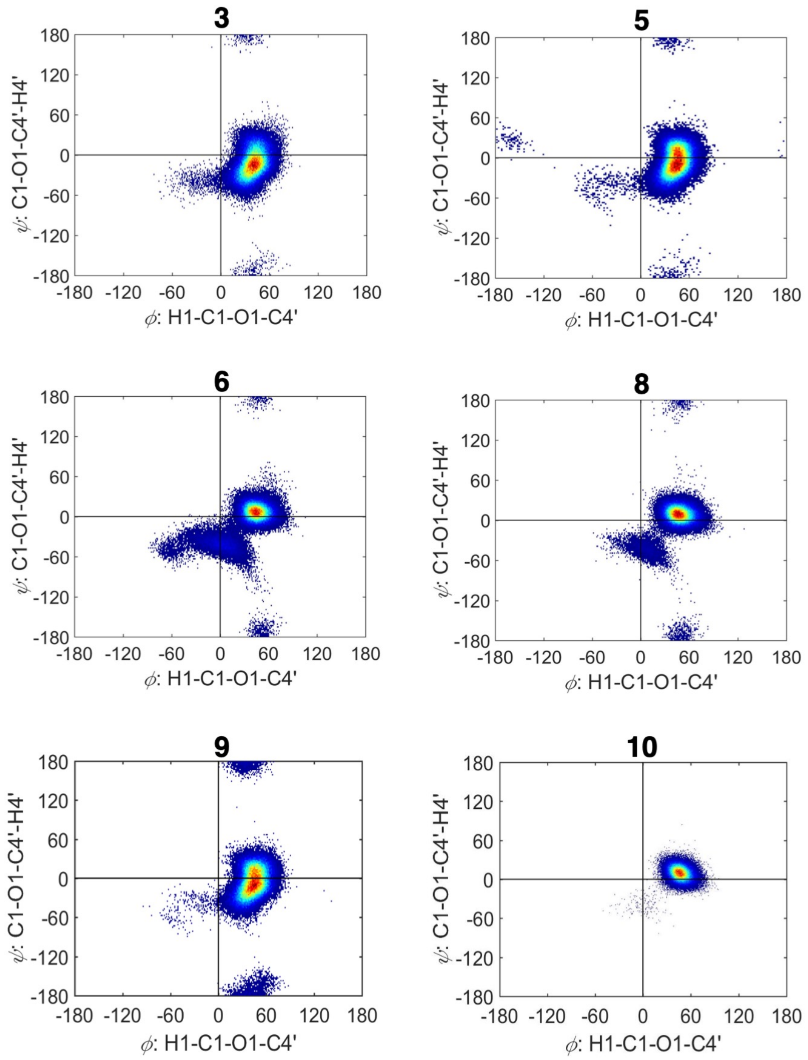

Figure S20. ϕ/ψ Contour plots for the βGlcNAc-(1→4)-βMan linkages obtained from 1-μs aqueous molecular dynamics simulations of disaccharide **3**, trisaccharides **5** and **6**, tetrasaccharides **8** and **9**, and hexasaccharide **10**.

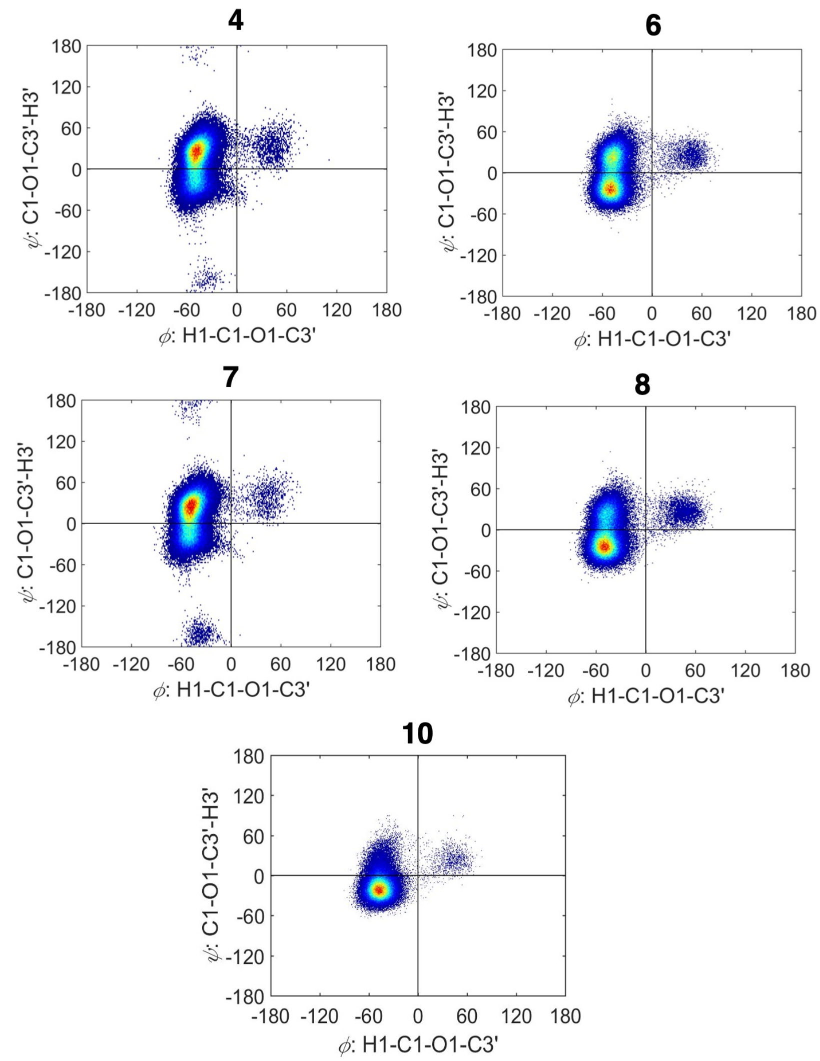

Figure S21. ϕ/ψ Contour plots for the αMan-(1→3)-βMan linkages obtained from 1-μs aqueous molecular dynamics simulations of disaccharide **4**, trisaccharides **6** and **7**, tetrasaccharide **8**, and hexasaccharide **10**.

Table S5. Back-Calculated *J*-Couplings Sensitive to ϕ and ψ in the βGlcNAc-(1→4)-βMan Linkages of **3**, **5**–**6** and **8**–**10** Obtained from 1-μs Aqueous MD Simulations.

compd	ϕ-dependent *J*-couplings (Hz)					ψ-dependent *J*-couplings (Hz)			
	$^{2}J_{C1',C4}$	$^{2}J_{C2',H1'}$	$^{3}J_{C2',C4}$	$^{3}J_{C4,H1'}$	RMSD	$^{3}J_{C1',C3}$	$^{3}J_{C1',C5}$	$^{3}J_{C1',H4}$	RMSD
**3**	–2.48	1.88	3.59	3.97	0.55	0.64	2.63	4.99	0.38
**5**	–2.53	1.99	3.63	3.56	0.75	1.01	2.04	5.23	0.67
**6**	–2.47	2.03	3.37	3.66	0.54	1.41	1.51	5.28	0.47
**8**	–2.55	2.10	3.57	3.21	0.77	1.71	0.99	5.53	0.68
**9**	–2.52	1.98	3.62	3.60	0.74	1.00	2.05	5.24	0.65
**10**	–2.56	2.05	3.68	3.26	0.76	1.85	0.77	5.61	0.71

Table S6. Back-Calculated *J*-Couplings Sensitive to ϕ and ψ in the αMan-(1→3)-βMan Linkages of **4** and **6**–**8** Obtained from 1-μs Aqueous MD Simulations.

compd	ϕ-dependent *J*-couplings (Hz)				ψ-dependent *J*-couplings (Hz)			
	$^{2}J_{C1',C3}$	$^{3}J_{C2',C3}$	$^{3}J_{C3,H1'}$	RMSD	$^{3}J_{C1',C2}$	$^{3}J_{C1',C4}$	$^{3}J_{C1',H3}$	RMSD
**4**	–2.00	4.56	3.73	0.62	0.76	2.14	5.06	0.41
**6**	–2.00	4.54	3.63	0.61	1.22	1.32	5.48	0.71
**7**	–2.01	4.58	3.68	0.58	0.72	2.16	5.11	0.42
**8**	–1.98	4.47	3.64	0.60	1.43	1.02	5.55	0.67

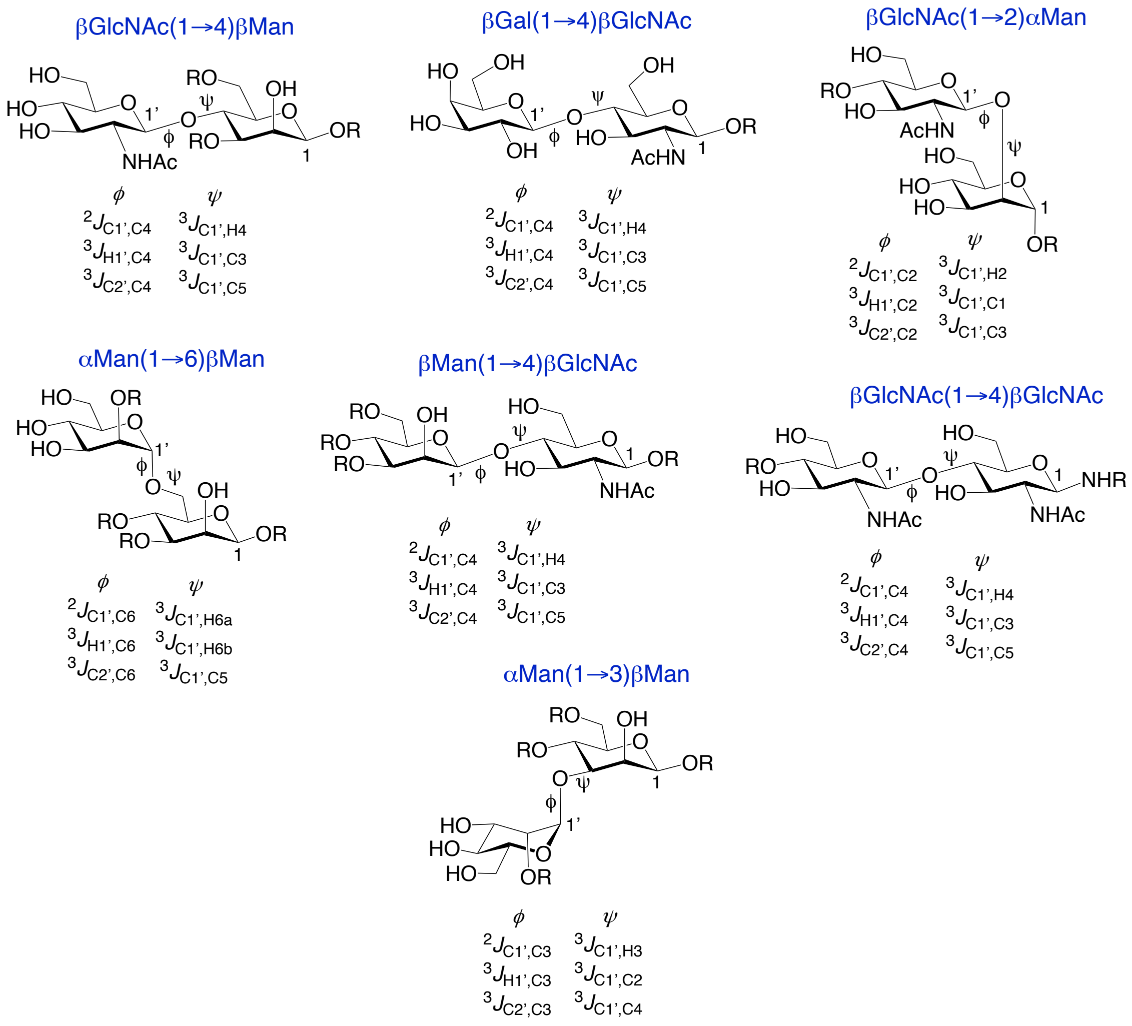

Scheme S5. The unique *O*-glycosidic linkages in oligosaccharide **1** (Scheme 1, text), showing six conventional trans-*O*-glycosidic *J*-couplings that depend on ϕ and ψ in each linkage. C1, C1', ϕ and ψ are identified in each structure.

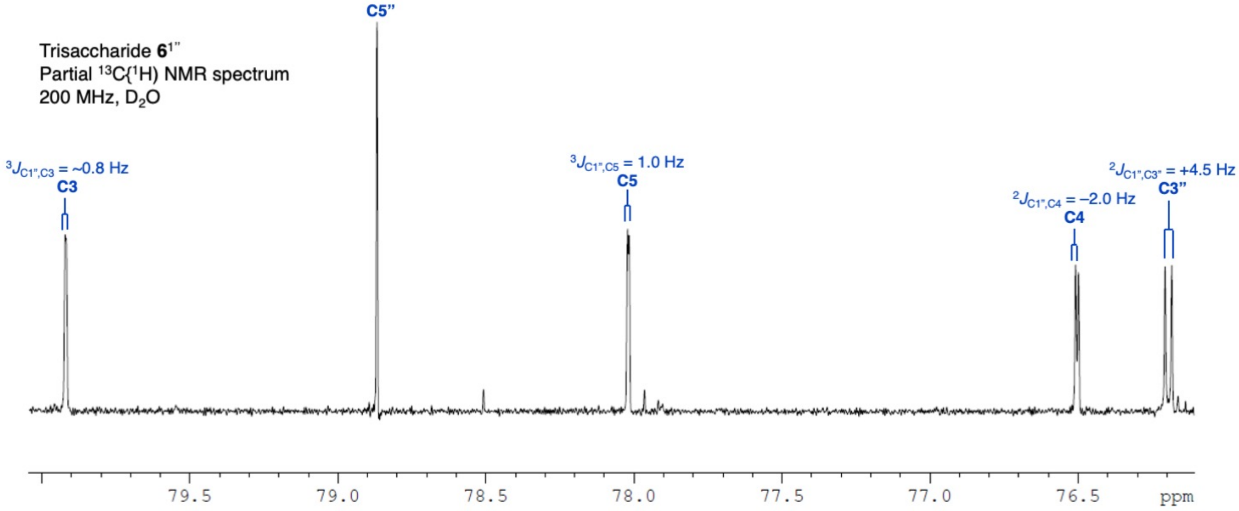

Figure S22. Partial $^{13}C\{^{1}H)$ NMR spectrum (200 MHz) of trisaccharide **6**$^{1''}$ in D_2O, showing splitting of the C3, C4, C5 and C3'' signals. J_{CC} values extracted from the doublets are shown, and coupling signs for the $^{2}J_{CC}$ values are indicated.

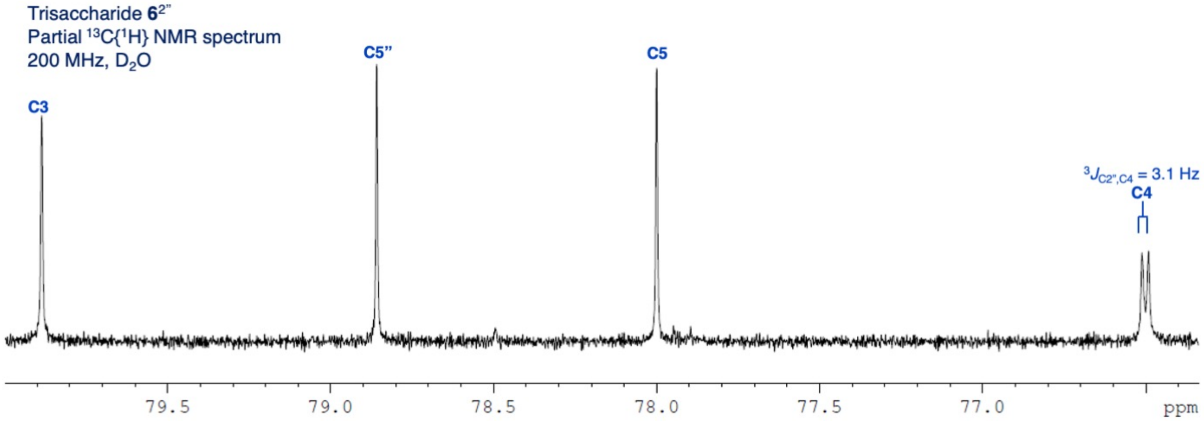

Figure S23. Partial $^{13}C\{^{1}H)$ NMR spectrum (200 MHz) of trisaccharide $\mathbf{6}^{2''}$ in D_2O, showing singlets from the C3, C5 and C5'' and a doublet for C4. The J_{CC} value extracted from the latter doublet is shown.

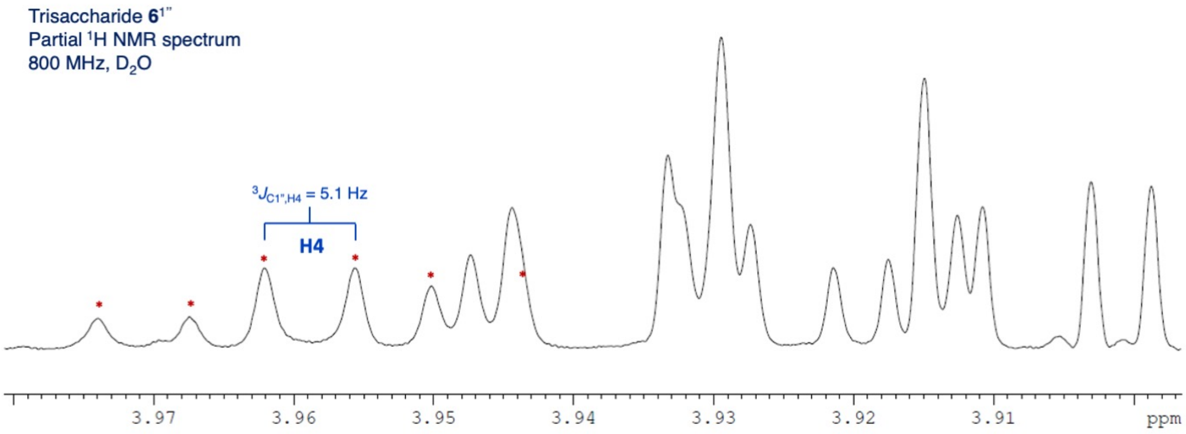

Figure S24. Partial ^{1}H NMR spectrum (800 MHz) of trisaccharide **6**$^{1''}$ in D_2O, showing the H4 multiplet (signals marked with a red asterick), from which a trans-*O*-glycosidic $^3J_{C1'',H4}$ value of 5.1 Hz was extracted.

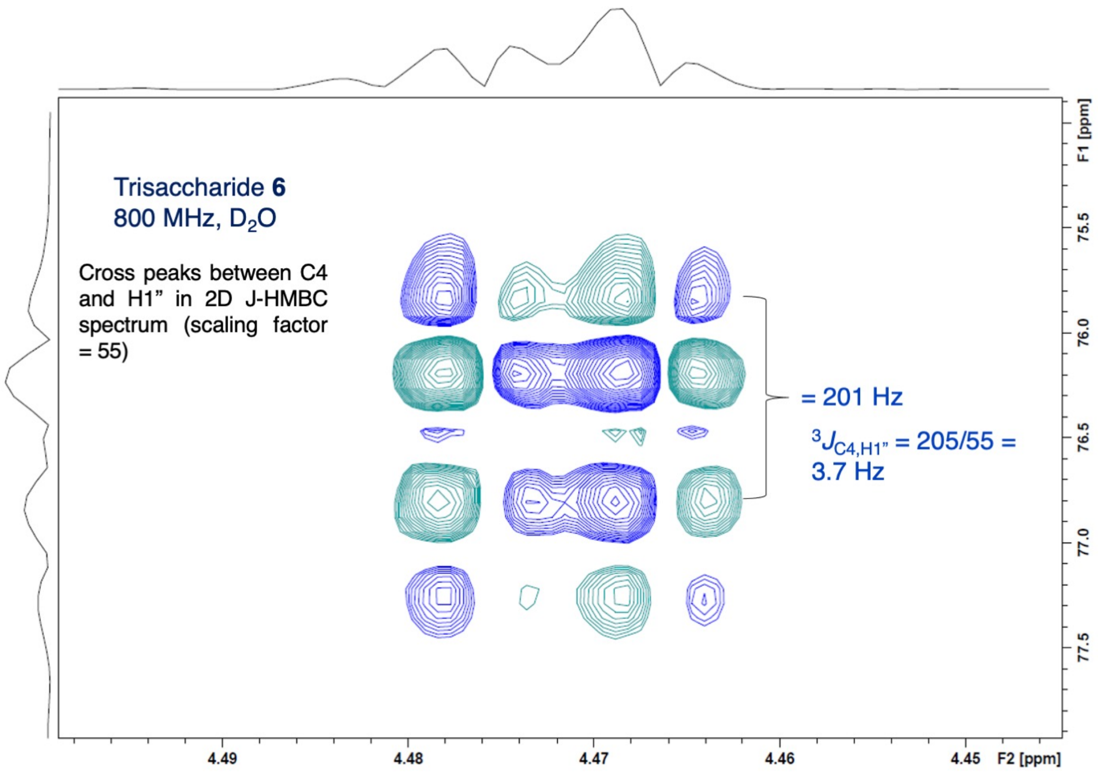

Figure S25. 2D J-HMBC spectrum (800 MHz) of **6** in D_2O showing the C4 and H1'' cross peaks. Using the scaling factor of 55, the trans-*O*-glycosidic $^3J_{C4,H1''}$ value of 3.7 Hz was extracted from the cross peaks.

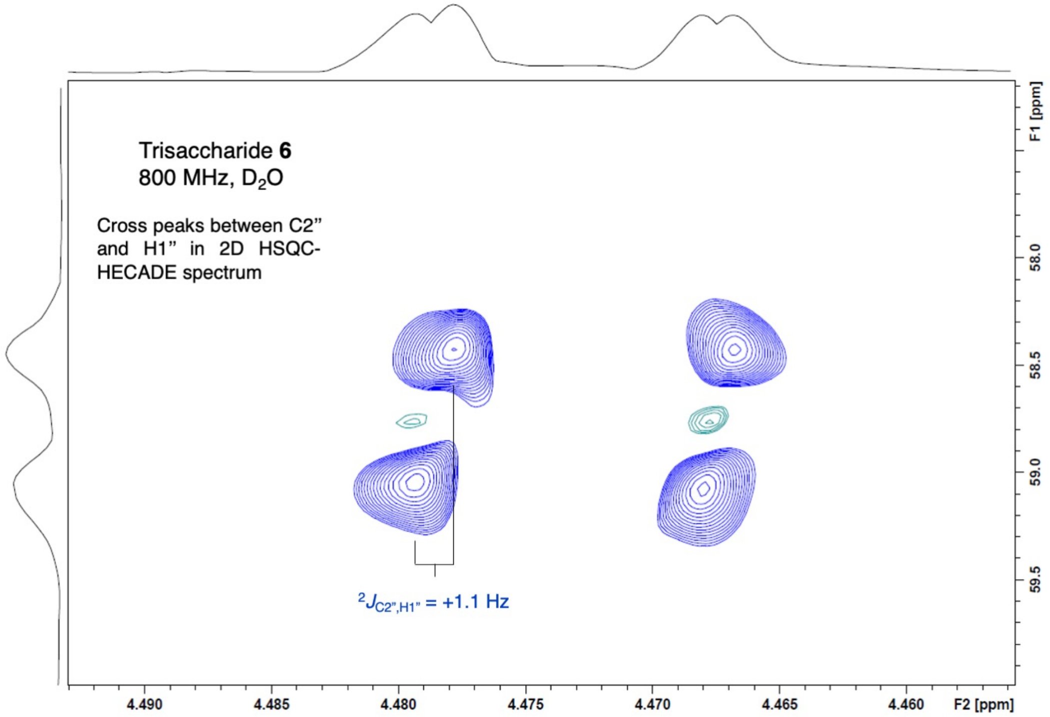

Figure S26. 2D HSQC-HECADE spectrum (800 MHz) of **6** in D_2O showing the C2'' and H1'' cross peaks. Using the observed *x*-axis offset of the contours, the trans-*O*-glycosidic $^2J_{C2'',H1''}$ value of +1.1 Hz was extracted from the cross peaks. The sign was determined by comparisons between the direction of the observed displacement alogthe x-axis and those associated with J_{CH} values having known positive signs ($^1J_{CH}$ and $^3J_{CH}$ values).

Table S7. Experimental (Top) and CASPER-Calculated[a] (Bottom) ^{1}H Chemical Shifts in Compounds **3**, **5**–**6** and **8**–**10**.

compound/ residue		1H chemical shifts (ppm) - experimental								
		H1	H2	H3	H4	H5	H6a	H6b	OCH3	COCH3
3	βMan	4.56	4.01	3.73	3.67	3.42	3.83	3.65	3.51	
	βGlcNAc′	4.52	3.73	3.54	3.44	3.50	3.92	3.73		2.05
5	βMan	4.55	4.01	3.74	3.71	3.55	3.82	3.79	3.51	
	βGlcNAc′	4.53	3.73	3.55	3.44	3.49	3.91	3.73		2.04
	αMan″	4.91	3.98	3.80	3.63	3.70	3.93	3.75		
6	βMan	4.54	4.05	3.87	3.89	3.37	3.88	3.67	3.50	
	αMan′	5.22	4.13	3.84	3.63	3.69	3.87	3.72		
	βGlcNAc″	4.47	3.67	3.52	3.37	3.41	3.95	3.71		2.03
8	βMan	4.52	4.04	3.85	3.94	3.49	3.82	3.82	3.48	
	αMan′	5.21	4.12	3.84	3.62	3.68	3.84	3.71		
	βGlcNAc″	4.49	3.67	3.51	3.36	3.38	3.95	3.70		2.01
	αMan‴	4.93	3.98	3.77	3.60	3.67	3.93	3.73		
9	βMan	4.54	4.01	3.72	3.72	3.53	3.82	3.80	3.51	
	GlcNAc′	4.52	3.72	3.55	3.44	3.49	3.92	3.73		2.06
	αMan1	5.16	4.02	3.91	3.67	3.67	3.93	3.75		
	αMan2	5.03	4.05	3.83	3.61	3.77	3.89	3.71		
10	βMan	4.52	4.03	3.79	3.98	3.47	3.85	3.83	3.79	
	βGlcNAc′	4.47	3.80	3.51	3.34	3.40	3.98	3.76		2.03
	αMan1	5.20	4.05	3.66	3.89	3.66	3.95	3.70		
	αMan2	5.03	4.04	3.82	3.58	3.75	3.89	3.76		
	αMan3	5.63	4.19	3.94	3.65	3.66	3.86	3.73		
	αMan4	5.04	4.01	3.81	3.61	3.81	3.88	3.78		

compound/ residue		1H chemical shifts (ppm) - CASPER (referenced to OMe shift)								
		H1	H2	H3	H4	H5	H6a	H6b	OCH3	COCH3
3	βMan	4.52	4.01	3.74	3.73	3.41	3.84	3.65	3.51	
	βGlcNAc′	4.59	3.73	3.58	3.47	3.50	3.91	3.74		2.06
5	βMan	4.52	4.01	3.72	3.78	3.53	3.86	3.70	3.51	
	βGlcNAc′	4.59	3.73	3.58	3.47	3.50	3.91	3.74		2.06
	αMan″	4.90	3.98	3.84	3.67	3.69	3.88	3.76		
6	βMan	4.58	4.22	3.70	3.90	3.44	3.84	3.66	3.50	
	αMan′	5.00	4.00	3.85	3.66	3.70	3.85	3.73		
	βGlcNAc″	4.53	3.70	3.58	3.33	3.49	3.94	3.69		2.06
8	βMan	4.56	4.20	3.66	3.93	3.54	3.84	3.69	3.48	
	αMan′	4.98	3.98	3.83	3.64	3.68	3.83	3.71		
	βGlcNAc″	4.51	3.68	3.56	3.31	3.47	3.92	3.67		2.04
	αMan‴	4.88	3.96	3.82	3.65	3.67	3.86	3.74		
9	βMan	4.51	4.02	3.71	3.77	3.51	4.00	3.53	3.51	
	GlcNAc′	4.61	3.75	3.60	3.49	3.52	3.93	3.76		2.08
	αMan1	5.12	4.02	3.96	3.69	3.69	3.88	3.76		
	αMan2	5.04	4.07	3.84	3.67	3.75	3.87	3.76		
10	βMan	4.54	4.18	3.62	3.91	3.50	3.96	3.49	3.46	
	βGlcNAc′	4.50	3.67	3.55	3.30	3.46	3.91	3.66		2.03
	αMan1	5.07	3.97	3.91	3.64	3.64	3.83	3.71		
	αMan2	4.99	4.02	3.79	3.62	3.70	3.82	3.71		
	αMan3	5.20	4.02	3.95	3.68	3.68	3.84	3.71		
	αMan4	5.03	4.03	3.81	3.65	3.69	3.82	3.73		

[a]CASPER-calculated values were referenced by assigning the calculated shift of the OCH_3 hydrogens to be identical to the experimental shift.

Table S8. Experimental (Top) and CASPER-Calculated[a] (Bottom) ^{13}C Chemical Shifts in Compounds **3**, **5**–**6** and **8**–**10**.

compound/ residue		13C chemical shifts (ppm) - Experimental								
		C1	C2	C3	C4	C5	C6	OCH3	COCH3	COCH3
3	βMan	103.57	72.36	74.41	80.18	77.43	63.17	59.50		
	βGlcNAc′	104.27	58.24	76.11	72.45	78.57	63.28		177.27	24.79
5	βMan	103.55	72.23	74.31	79.76	76.21	68.72	59.53		
	βGlcNAc′	104.09	58.32	76.06	72.49	78.65	63.30		177.25	24.89
	αMan″	102.80	72.57	73.13	69.49	75.80	63.82			
6	βMan	103.45	72.73	79.86	76.52	77.99	62.99	59.50		
	αMan′	104.38	72.46	73.08	69.52	76.05	63.71			
	βGlcNAc″	103.85	58.77	76.17	72.89	78.86	64.19		177.28	24.83
8	βMan	103.42	72.51	79.82	76.09	76.59	68.43	59.49		
	αMan′	104.37	72.47	73.05	69.49	76.01	63.68			
	βGlcNAc″	103.65	58.77	76.08	72.94	79.10	64.16		177.27	24.82
	αMan‴	102.83	72.53	73.20	69.50	75.94	63.85			
9	βMan	103.62	72.24	74.31	79.55	76.05	68.69	59.56		
	βGlcNAc′	104.04	58.32	76.01	72.49	78.66	63.29		177.27	24.94
	αMan1	101.13	80.99	72.84	69.63	75.94	63.83			
	αMan2	104.83	72.61	72.97	69.53	75.99	63.81			
10	βMan	103.55	72.57	79.69	75.82	76.54	68.39	59.53		
	βGlcNAc′	103.98	58.77	76.62	73.17	79.21	63.95		177.47	24.82
	αMan1	101.31	80.77	72.96	69.64	76.19	63.88			
	αMan2	104.76	72.59	72.96	69.57	76.05	63.92			
	αMan3	102.45	82.45	72.37	69.86	75.82	63.80			
	αMan4	105.11	72.88	73.20	69.59	75.44	63.46			

compound/ residue		13C chemical shifts (ppm) - CASPER (referenced to OMe shift)								
		C1	C2	C3	C4	C5	C6	OCH3	COCH3	COCH3
3	βMan	103.59	72.30	74.31	79.25	77.54	63.18	59.50		
	βGlcNAc′	104.08	58.35	76.24	72.61	78.60	63.39		177.09	24.83
5	βMan	103.74	72.23	74.39	79.13	75.83	68.21	59.53		
	βGlcNAc′	104.11	58.38	76.27	72.64	78.63	63.42		177.12	24.86
	αMan″	102.38	72.68	73.35	69.63	75.43	63.75			
6	βMan	103.37	72.71	81.09	76.12	78.19	63.22	59.50		
	αMan′	105.64	73.01	73.56	69.51	76.30	63.53			
	βGlcNAc″	103.28	58.51	76.14	72.64	78.95	63.40		177.02	24.76
8	βMan	103.48	72.60	81.13	75.96	76.44	68.21	59.49		
	αMan′	105.63	73.00	73.55	69.50	76.29	63.52			
	βGlcNAc″	103.27	58.50	76.13	72.63	78.94	63.39		177.01	24.75
	αMan‴	102.27	72.57	73.24	69.52	75.32	63.64			
9	βMan	103.75	72.16	74.29	79.07	75.73	68.32	59.56		
	βGlcNAc′	104.14	58.41	76.30	72.67	78.66	63.45		177.15	24.89
	αMan1	100.90	80.98	72.92	69.83	75.47	63.75			
	αMan2	104.83	72.71	73.16	69.65	75.89	63.82			
10	βMan	103.48	72.54	81.07	75.95	76.35	68.19	59.53		
	βGlcNAc′	103.32	58.55	76.18	72.68	78.99	63.44		177.06	24.80
	αMan1	100.81	80.89	72.83	69.74	75.38	63.66			
	αMan2	104.74	72.62	73.07	69.56	75.80	63.73			
	αMan3	104.14	81.12	73.30	69.75	76.40	63.70			
	αMan4	104.73	72.66	73.11	69.51	75.84	63.66			

[a]CASPER-calculated values were referenced by assigning the calculated shift of the OCH_3 carbon to be identical to the experimental value.

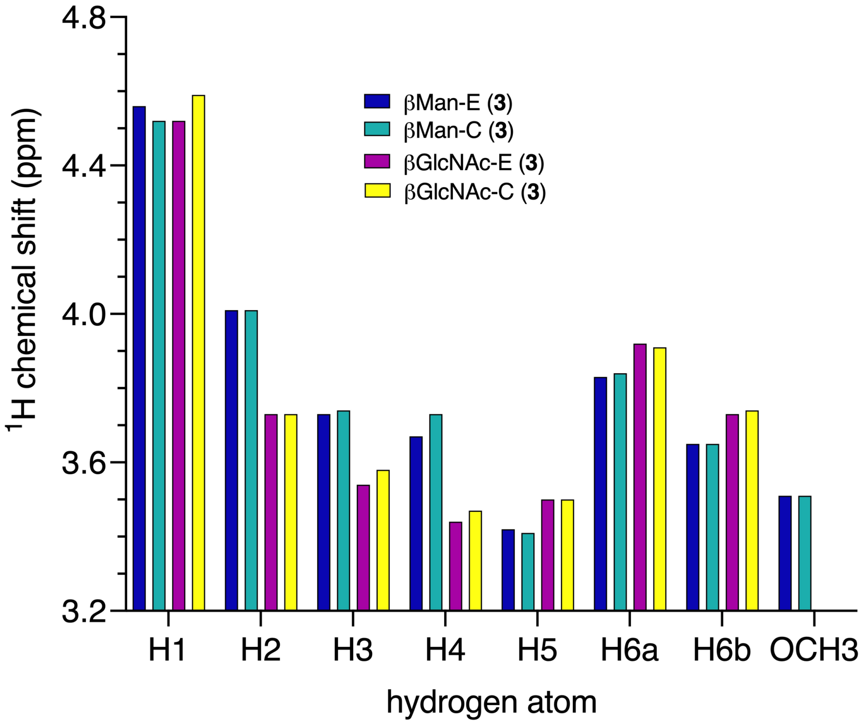

Figure S27. Comparisons of experimental (E) and CASPER-calculated (C) ^{1}H chemical shifts in the βMan (blue and green bars) and βGlcNAc (red and yellow bars) residues of **3**.

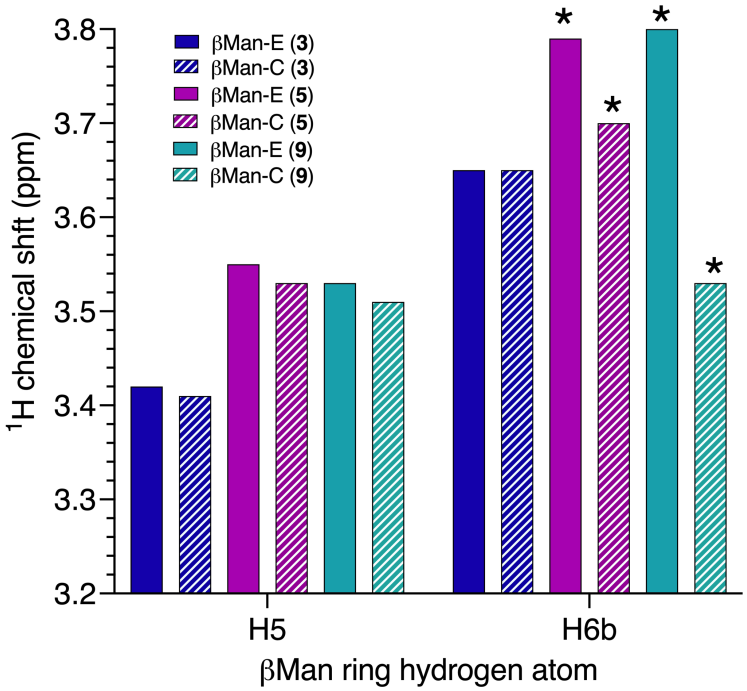

Figure S28. Comparisons of experimental (E; solid bars) and CASPER-calculated (C; hatched bars) H5 and H6b chemical shifts in the βMan residues of **3**, **5** and **9**.

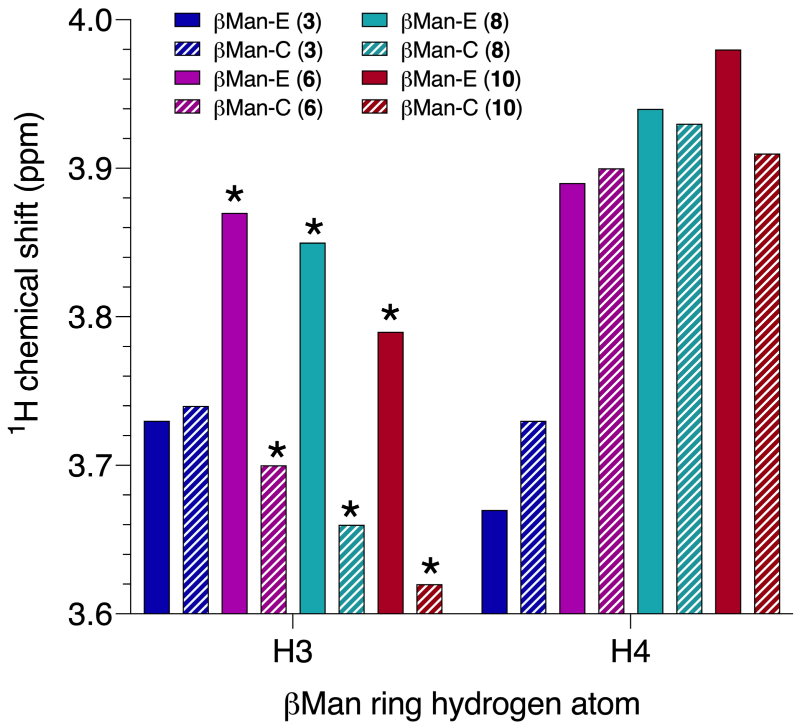

Figure S29. Comparisons of experimental (E; solid bars) and CASPER-calculated (C; hatched bars) H3 and H4 chemical shifts in the βMan residues of **3**, **6**, **8** and **10**.

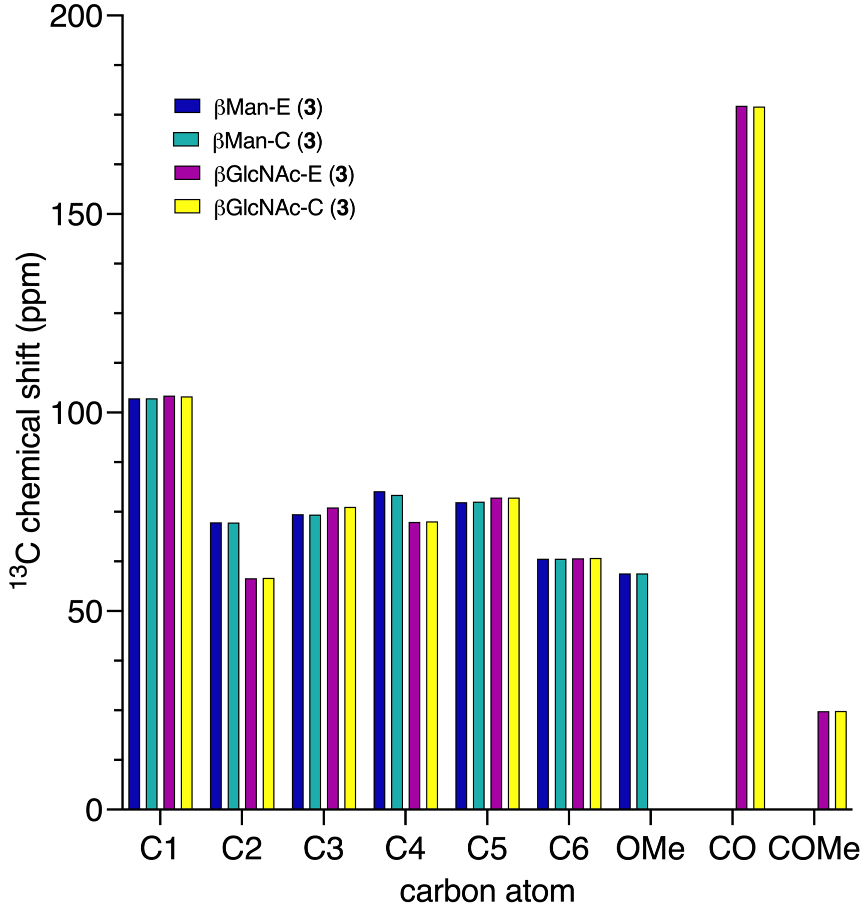

Figure S30. Comparisons of experimental (E) and CASPER-calculated (C) ^{13}C chemical shifts in the βMan (blue and green bars) and βGlcNAc (red and yellow bars) residues of **3**.

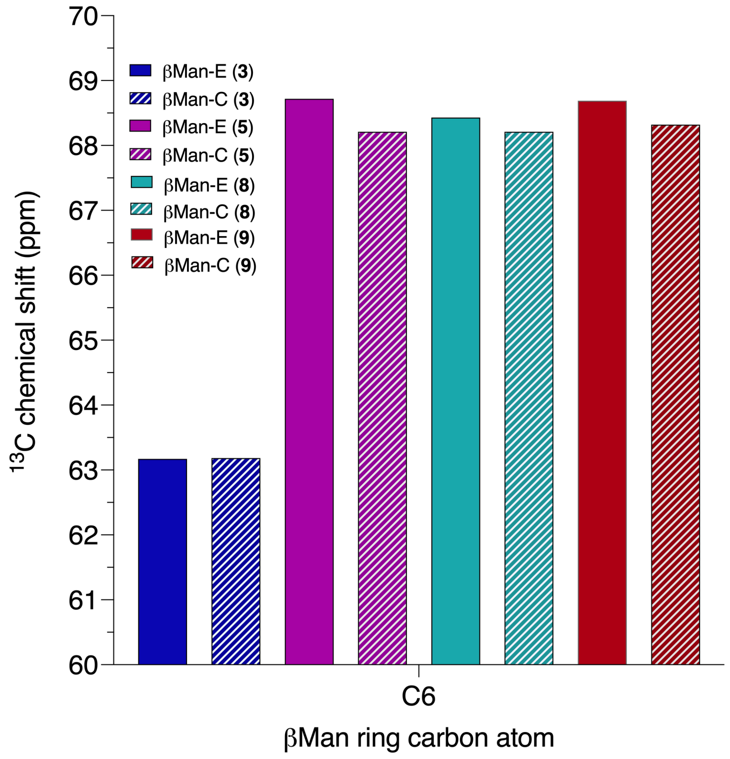

Figure S31. Comparisons of experimental (E; solid bars) and CASPER-calculated (C; hatched bars) C6 chemical shifts in the βMan residues of **3**, **5**, **8** and **9**.

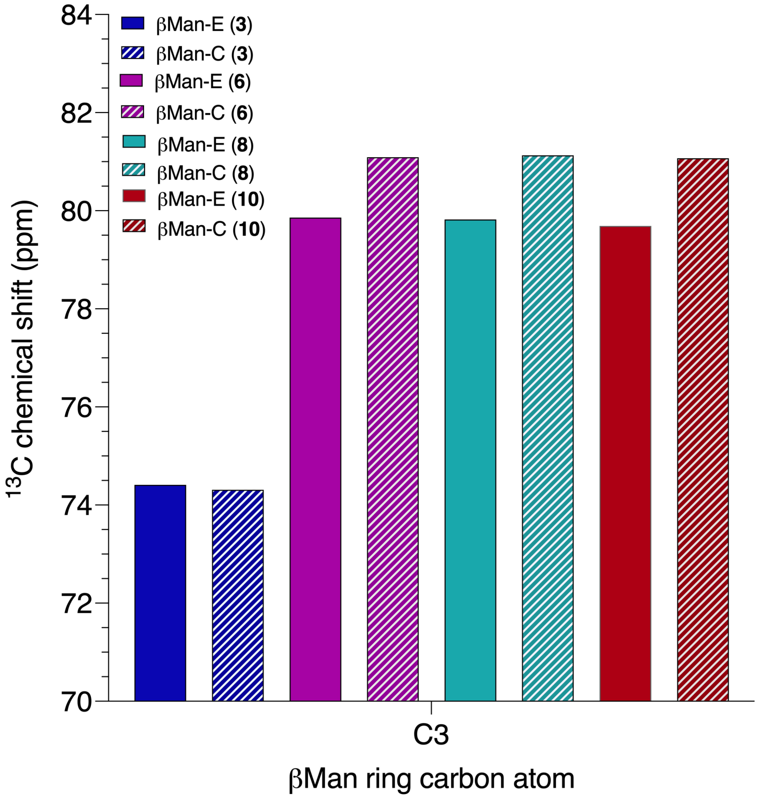

Figure S32. Comparisons of experimental (E; solid bars) and CASPER-calculated (C; hatched bars) C3 chemical shifts in the βMan residues of **3**, **6**, **8** and **10**.

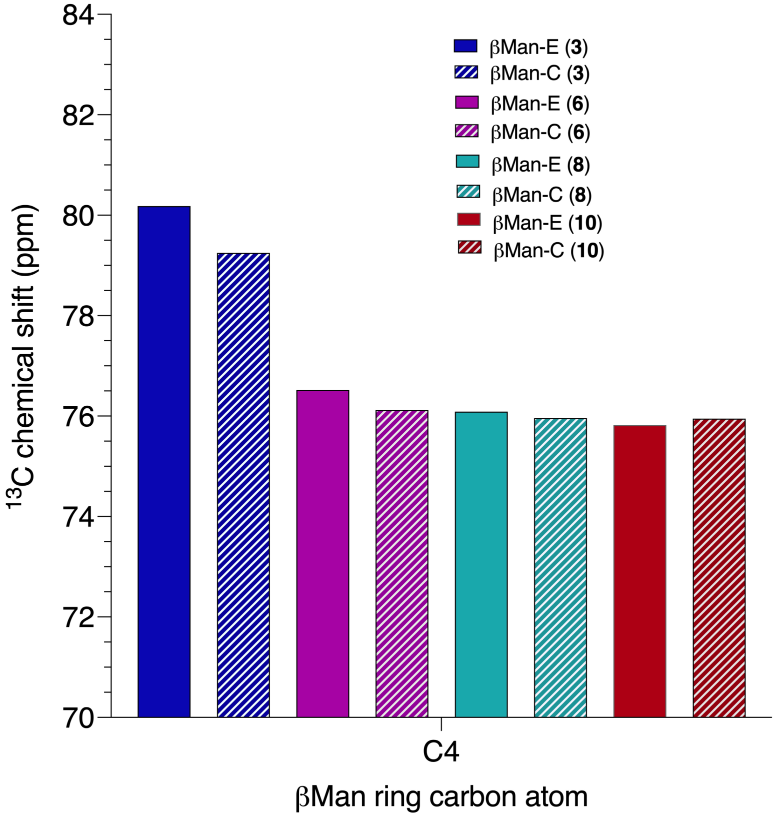

Figure S33. Comparisons of experimental (E; solid bars) and CASPER-calculated (C; hatched bars) C4 chemical shifts in the βMan residues of **3**, **6**, **8** and **10.**

Discussion of Data Presented in Tables S7–S8 and Figures S27–33

Tables S7 and S8 contain ^{1}H and ^{13}C chemical shifts, respectively, in **3**, **5**, **6** and **8–10** that were measured experimentally (hereafter denoted *E* shifts) or calculated (hereafter denoted *C* shifts) by the CASPER method (20–22). To allow comparisons between *E* and *C* shifts, the *C* shifts of the aglycone methyl hydrogens (for the ^{1}H data) or carbon (for the ^{13}C data) in disaccharide **3** were made to be equal to the *E* shifts, with concomitant adjustments of all remaining *C* shifts in **3** and in the remaining compounds.

Calculated chemical shifts were obtained to determine whether differences in chemical shifts of corresponding nuclei in **5**, **6** and **8–10** might be attributed to conformational factors. The underlying assumption is that chemical shifts calculated by CASPER would capture substitution (through-bond) effects mainly, and that conformational contributions to the calculated values are negligible. If this is the case, then large differences between *E* and *C* values might be indicative of conformational differences. The molecular origin of these putative conformational differences, however, would not be assignable, since they could arise from multiple sources such as changes in pyranosyl ring conformation, *O*-glycosidic linkage conformation, and/or exocyclic side-chain conformation (e.g., hydroxyl or hydroxymethyl groups).

The plot shown in Figure S27 compares *E* and *C* ^{1}H shifts for the βMan and βGlcNAc residues of disaccharide **3**. The agreement between corresponding *E* and *C* values is very good as expected, since context effects are small in **3** and CASPER is likely to reliably recapitulate the experimental data.

The plot shown in Figure S28 compares *E* and *C* shifts of H5 and H6b in the βMan residues of **3**, **5** and **9**. Note that for H5, the hatched bars track the solid bars very well, such that the changes in δ_{H5} as **3** converts to **5** and **9** are unlikely to be caused by conformational changes. On the other hand, the results for δ_{H6b} suggest that the different chemical shifts in **5** and **9** relative to **3** may be conformational in origin, since the *C* values deviate significantly from the *E* values in the former. An inspection of Figure S29 leads to the same conclusions for δ_{H3} in **6**, **8** and **10**, all of which show large deviations between *E* and *C* values. In contrast, changes in δ_{H4} as **3** is converted to **6**, **8** and **10** are captured well by CASPER, indicating that these changes are caused mainly by substitution effects.

As shown in Figure S27 for ^{1}H shifts, the results shown in Figure S30 show that CASPER captures the carbon chemical shifts for the βMan and βGlcNAc residues of **3** very well, as expected since context effects in **3** are small and CASPER is likely to reliably recapitulate the experimental values. An inspection of Figure S31 reveals that *C* shifts for the βMan residues in **3**, **5**, **8** and **9** are in good agreement with corresponding *E* shifts, indicating that the downfield shift in δ_{C6} in **5**, **8** and **9** relative to that in **3** is mainly a substitution effect. The same conclusion can be drawn for δ_{C3} data shown in Figure S32, wherein CASPER recapitulates the significant downfield shift of δ_{C3} in the βMan residue of **6**, **8** and **10** relative to that in **3**. However, the calculated δ_{C3} values in **6**, **8** and **10** are consistently larger than corresponding *E* values, possibly indicating a conformational contribution. Data shown in Figure S33 show that CASPER predicts a significant upfield shift in δ_{C4} of the βMan residue as **3** is converted to **6**, **8** and **10**. The good agreement between the *E* and *C* shifts for **6**, **8** and **10** is consistent with the upfield shift being largely a substitution effect.

Cartesian Coordinates for a DFT-Optimized Conformer of 3[c]

(Torsion angles ϕ and ψ are defined as C2'–C1'–O1'–C4 and C1'–O1'–C3–C3, respectively)

ϕ = 150°, ψ = 150°.

C	1.443	-0.147	-0.424
C	2.197	0.964	0.343
C	3.701	0.891	0.050
C	4.239	-0.530	0.234
C	3.416	-1.511	-0.608
C	3.802	-2.974	-0.434
C	-3.775	-0.577	-1.252
C	-3.167	-1.801	-0.556
C	-1.899	-1.362	0.166
C	-0.916	-0.661	-0.785
C	-1.661	0.533	-1.432
C	-0.879	1.342	-2.453
O	0.148	-0.237	0.085
H	1.446	0.054	-1.505
H	2.023	0.802	1.410
H	3.876	1.166	-1.005
H	4.152	-0.803	1.294
H	3.500	-1.245	-1.674
H	4.832	-3.124	-0.770
H	3.744	-3.239	0.634
H	-4.128	0.162	-0.511
H	-3.889	-2.182	0.183
H	-2.190	-0.618	0.927
H	-0.547	-1.362	-1.540
H	-1.964	1.216	-0.622
H	-0.755	0.746	-3.369
H	0.112	1.579	-2.055
H	1.786	2.608	-0.935
H	0.182	5.048	1.103
H	-0.440	4.109	-0.278
H	1.166	4.849	-0.372
N	1.668	2.275	0.014
O	2.046	-1.419	-0.191
O	5.611	-0.485	-0.166
H	2.063	-3.581	-0.998
O	-1.294	-2.484	0.787
O	-2.820	0.037	-2.109
H	5.324	1.612	0.773
C	1.035	3.091	0.910
C	-5.596	-0.019	-2.676
H	6.110	-1.124	0.366

O	-2.850	-2.803	-1.511
O	4.377	1.798	0.906
O	-4.829	-1.042	-2.035
H	-3.585	-2.796	-2.149
O	2.978	-3.822	-1.222
H	-0.424	-2.172	1.093
O	-1.610	2.542	-2.705
C	0.457	4.359	0.303
O	0.915	2.807	2.101
H	-6.414	-0.525	-3.192
H	-4.990	0.536	-3.398
H	-6.007	0.677	-1.933
H	-1.116	3.048	-3.369

Full Literature References 61 and 76

Reference 61

Frisch, M. J., Trucks, G. W., Schlegel, H. B., Scuseria, G. E., Robb, M. A., Cheeseman, J. R., Scalmani, G., Barone, V., Petersson, G. A., Nakatsuji, H., Li, X., Caricato, M., Marenich, A. V., Bloino, J., Janesko, B. G., Gomperts, R., Mennucci, B., Hratchian, H. P., Ortiz, J. V., Izmaylov, A. F., Sonnenberg, J. L., Williams-Young, D., Ding, F., Lipparini, F., Egidi, F., Goings, J., Peng, B., Petrone, A., Henderson, T., Ranasinghe, D., Zakrzewski, V. G., Gao, J., Rega, N., Zheng, G., Liang, W., Hada, M., Ehara, M., Toyota, K., Fukuda, R., Hasegawa, J., Ishida, M., Nakajima, T., Honda, Y., Kitao, O., Nakai, H., Vreven, T., Throssell, K., Montgomery, J. A., Jr., Peralta, J. E., Ogliaro, F., Bearpark, M. J., Heyd, J. J., Brothers, E. N., Kudin, K. N., Staroverov, V. N., Keith, T. A., Kobayashi, R., Normand, J., Raghavachari, K., Rendell, A. P., Burant, J. C., Iyengar, S. S., Tomasi, J., Cossi, M., Millam, J. M., Klene, M., Adamo, C., Cammi, R., Ochterski, J. W., Martin, R. L., Morokuma, K., Farkas, O., Foresman, J. B., and Fox, D. J. *Gaussian* 16, Revision B.01, Gaussian, Inc., Wallingford, CT, 2016.

Reference 76

Case, D. A., Babin, V., Berryman, J. T., Betz, R. M., Cai, Q., Cerutti, D. S., Cheatham, T. E. I., Darden, T. A., Duke, R. E., Gohlke, H., Goetz, A. W., Gusarov, S., Homeyer, N., Janowski, P., Kaus, J., Kolossváry, I., Kovalenko, A., Lee, T. S., LeGrand, S., Luchko, T., Luo, R., Madej, B., Merz, K. M., Paesani, F., Roe, D. R., Roitberg, A., Sagui, C., Salomon-Ferrer, R., Seabra, G., Simmerling, C. L., Smith, W., Swails, J., Walker, R. C., Wang, J., Wolf, R. M., Wu, X., and Kollman, P. A. 2014, AMBER 14, University of California, San Francisco.

Literature References

1. Zhang, W., Pan, Q., and Serianni, A. S. (2016) A chemical synthesis of a multiply ^{13}C-labeled hexasaccharide: A high-mannose *N*-glycan fragment. *J. Labelled Compd. Radiopharm.* **59**, 673–679

2. Qin, H., and Grindley, T. B. (1994) Regioselective formation of di-*O*-benzyl-substituted hexopyranosides via stannylene acetal intermediates. *J. Carbohydr. Chem.* **13,** 475-490

3. Zhou, Y., Li, J., Zhan, Y., Pei, Z., and Dong, H. (2013) Halide promoted organotin-mediated carbohydrate benzylation: Mechanism and application. *Tetrahedron* **69**, 2693–2700

4. Du, Y., Zhang, M., and Kong, F. (2001) Efficient and practical syntheses of three pentasaccharides core structures corresponding to *N*-glycans. *Tetrahedron* **57**, 1757–1763

5. Schmidt, R. R., and Michel, J. (1980) Facile synthesis of α-and β-*O*-glycosyl imidates: Preparation of glycosides and disaccharides. *Angew. Chem., Int. Ed. Engl.* **19,** 731–732

6. Capon, B., and McManus, S. P. Neighboring group participation. Plenum Press: New York: 1976

7. Demchenko, A. V. *Handbook of chemical glycosylation: Advances in stereoselectivity and therapeutic relevance*. John Wiley & Sons: 2008

8. Zhang, W., Zhao, H., Carmichael, I., and Serianni, A. S. (2009) An NMR investigation of putative interresidue H-bonding in methyl α-cellobioside in solution. *Carbohydr. Res.* **344**, 1582–1587

9. Battistel, M. D., Pendrill, R., Widmalm, G., and Freedberg, D. (2013) Direct evidence for hydrogen bonding in glycans: A combined NMR and molecular dynamics study. *J. Phys. Chem. B* **117**, 4860–4869

10. Crich, D., and Dudkin, V. (2001) Why are the hydroxy groups of partially protected *N*-acetylglucosamine derivatives such poor glycosyl acceptors, and what can be done about it? A comparative study of the reactivity of *N*-acetyl-, *N*-phthalimido-, and 2-azido-2-deoxy-glucosamine derivatives in glycosylation. 2-Picolinyl ethers as reactivity-enhancing replacements for benzyl ethers. *J. Am. Chem. Soc.* **123**, 6819–6825

11. Nagasaki, M., Manabe, Y., Minamoto, N., Tanaka, K., Silipo, A., Molinaro, A., and Fukase, K. (2016) Chemical synthesis of a complex-type *N*-glycan containing a core fucose. *J. Org. Chem.* **81**, 10600–10616

12. Manabe, Y., Shomura, H., Minamoto, N., Nagasaki, M., Takakura, Y., Tanaka, K., Silipo, A., Molinaro, A., and Fukase, K. (2018) Convergent synthesis of a bisecting *N*-acetylglucosamine (GlcNAc)-containing *N*-glycan. *Chem. Asian J.* **13**, 1544–1551

13. Zhang, W., Meredith, R., Pan, Q., Wang, X., Woods, R. J., Carmichael, I., and Serianni, A. S. (2019) Use of circular statistics to model αMan-(1→2)-αMan and αMan-(1→3)-α/βMan *O*-glycosidic linkage conformation in ^{13}C-labeled disaccharides and high-mannose oligosaccharides. *Biochemistry* **58**, 546–560

14. Debenham, S. D., and Toone, E. J. (2000) Regioselective reduction of 4,6-*O*-benzylidenes using triethylsilane and $BF_3 \cdot Et_2O$. *Tetrahedron: Asymmetry* **11**, 385–387

15. Zhang, W., Wang, J., Serianni, A. S., and Pan, Q. (2020) A convenient synthesis of short-chain α-(1→2) mannopyranosyl oligosaccharides. *Carbohydr. Res.* **489***,* 107897

16. Wang, W., and Kong, F. (1999) Regio-and stereoselective synthesis of oligosaccharides with acetobromolactose and acetobromomaltose as glycosyl donors via orthoester intermediates. *J. Carbohydr. Chem*. **18**, 451–460

17. Zhang, W., Zhao, S., and Serianni, A. S. (2015) Labeling monosaccharides with stable isotopes. *Methods Enzymol*. **565**, 423–458

18. Zhu, Y., Pan, Q., Thibaudeau, C., Zhao, S., Carmichael, I., and Serianni, A. S. (2006) [^{13}C,^{15}N]2-Acetamido-2-deoxy-D-aldohexoses and their methyl glycosides: Synthesis and NMR investigations of *J*-couplings involving ^{1}H, ^{13}C and ^{15}N. *J. Org. Chem.* **71**, 466–479

19. Bose-Basu, B., Klepach, T., Bondo, G., Bondo, P. B., Zhang, W., Carmichael, I., and Serianni, A. S. (2007) ^{13}C-^{13}C NMR spin-spin coupling constants in saccharides: Structural correlations involving all carbons in aldohexopyranosyl rings. *J. Org. Chem*. **72,** 7511–7522

20. Jansson, P. E.; Stenutz, R.; Widmalm, G. (2006) Sequence determination of oligosaccharides and regular polysaccharides using NMR spectroscopy and a novel web-based version of the computer program CASPER. *Carbohydr. Res*. **341**, 1003–1010

21. Lundborg, M.; Widmalm, G. (2011) Structure analysis of glycans by NMR chemical shift prediction. *Anal. Chem*. **83**, 1514–1517

22. Furevi, A.; Ruda, A.; d'Ortoli, T. A.; Mobarak, H.; Ståhle, J.; Hamark, C.; Fontana, C.; Engstr.m, O.; Apostolica, P.; Widmalm, G. (2022) Complete ^{1}H and ^{13}C NMR chemical shift assignments of mono- to tetrasaccharides as basis for NMR chemical shift predictions of oligo- and polysaccharides using the computer program CASPER. *Carbohydr. Res*. **513**, No. 108528
